# Pcyt2 deficiency causes age-dependant development of nonalcoholic steatohepatitis and insulin resistance that could be attenuated with phosphonoethylamine

**DOI:** 10.1038/s41598-022-05140-y

**Published:** 2022-01-20

**Authors:** Sophie Grapentine, Rathnesh K. Singh, Poulami Basu, Sugashan Sivanesan, Gabriela Mattos, Oreoluwa Oresajo, Jasmine Cheema, Wendwesen Demeke, Vernon W. Dolinsky, Marica Bakovic

**Affiliations:** 1 Department of Human Health and Nutritional Sciences, University of Guelph, 50 Stone Rd E, Guelph, N1G2W1 Canada; 2 Department of Pharmacology and Therapeutics, University of Manitoba, Winnipeg, Canada

**Keywords:** Physiology, Diseases, Endocrinology, Gastroenterology, Pathogenesis

## Abstract

The mechanisms of NASH development in the context of age and genetics are not fully elucidated. This study investigates the age-dependent liver defects during NASH development in mice with heterozygous deletion of Pcyt2 (*Pcyt2*^+*/−*^)*,* the rate limiting enzyme in phosphatidylethanolamine (PE) synthesis. Further, the therapeutic potential of the artificial Pcyt2 substrate, phosphonoethylamine (PEA), is examined. *Pcyt2*^+*/−*^ were investigated at 2 and 6–8 months (mo) of age and in addition, 6-mo old *Pcyt2*^+*/−*^ with developed NASH were supplemented with PEA for 8 weeks and glucose and fatty acid metabolism, insulin signaling, and inflammation were examined. Heterozygous ablation of Pcyt2 causes changes in liver metabolic regulators from young age, prior to the development of liver disease which does not occur until adulthood. Only older *Pcyt2*^+*/−*^ experiences perturbed glucose and fatty acid metabolism. Older *Pcyt2*^+*/−*^ liver develops NASH characterized by increased glucose production, accumulation of TAG and glycogen, and increased inflammation. Supplementation with PEA reverses *Pcyt2*^+*/−*^ steatosis, inflammation, and other aspects of NASH, showing that was directly caused by *Pcyt2* deficiency. Pcyt2 deficiency is a novel mechanism of metabolic dysregulation due to reduced membrane ethanolamine phospholipid synthesis, and the artificial Pcyt2 substrate PEA offers therapeutic potential for NASH reversion.

## Introduction

The progression of liver disease from simple steatosis to a dangerous state that includes inflammation and fibrosis, known as nonalcoholic steatohepatitis (NASH), is incompletely understood. NASH is tightly associated with obesity, diabetes and metabolic syndrome and can further advance to cirrhosis, hepatocellular carcinoma, and death^1^. It is predicted that by year 2030 NASH prevalence will have increased by 63% and liver related deaths by 178% from 2015^2^. Currently, therapy is restricted to lifestyle interventions as there is no pharmacological treatment for NASH^1^, highlighting the urgency for improved insight into NASH development.

Phosphatidylethanolamine (PE) is an important lipid component of cellular membranes where it is involved with essential processes including cell division, cell signalling, membrane fusion, autophagy, and apoptosis^3^. The major pathway for PE synthesis is via the CDP-ethanolamine Kennedy pathway^4^. First, ethanolamine kinase (EK) phosphorylates ethanolamine (Etn) to phosphoethanolamine (PEtn). In the second and rate limiting step of the Kennedy pathway, CTP-phosphoethanolamine cytidylyltransferase (Pcyt2) transfers CTP to PEtn to form CDP-Etn and pyrophosphate. Lastly, CDP-ethanolamine:1,2-diacylglycerol ethanolamine-phosphotransferase (EPT) catalyzes the condensation of CDP-Etn and diacylglycerol (DAG) to produce PE and CMP^4^. The production of PE can be modulated by availability of substrates ethanolamine and DAG^5^, however under most physiological conditions Pcyt2 is the predominant regulator of PE synthesis^3^. Pcyt2 protein exists as three isoforms encoded by a single gene: the catalytically active *Pcyt2α* and *Pcyt2β*, generated via exon skipping mechanism, and the third isoform, intron retention variant *Pcyt2γ* which functions as a negative regulator of enzyme activity^6^. Human PCYT2 and mouse *Pcyt2* genes are similarly regulated with LXR, EGR1, and NFkB transcription factors showing their conserved role in lipid metabolism, cell growth, and inflammation^7^. In addition, Pcyt2’s activity is stimulated by nutrient deficiency and PKCα/β phosphorylation at αSer^215^ and αSer^223^/βSer^205^ residues^8^.

Mounting evidence suggests that disruptions in PE homeostasis are linked to obesity and the development of diabetes and fatty liver diseases^9–12^. Recent human studies have negatively correlated the expression of PCYT2 with BMI in obese/overweight cancer patients^10^ and in an obese cohort^11^. On the other hand, the expression of PE degrading genes, PE methyltransferase (PEMT) and phosphatidylserine (PS) synthase 2 (PSS2), positively correlate with BMI^11^. Moreover, comparison of adipocytes to human visceral adipose tissue from obese subjects have suggested the negative association of PCYT2 expression with type 2 diabetes and insulin resistance^12^. Previously, we established that the enzymatic activity of Pcyt2 is drastically reduced through the induction of mutations at PKC phosphorylation sites^8^ and two catalytic sites^13^. Severe consequences of PCYT2 mutation and resultant diminished enzymatic activity we recently demonstrated in patients with multiple mutations that caused autosomal spastic paraplegia and profound lipid abnormalities^14,15^. According to dbSNP database PCYT2 is heavily mutated, with over 4400 single nucleotide polymorphisms identified and 60 allele mutations within the gene coding region^16^. Together this suggests the candidacy of PCYT2 gene and importance of establishing the significance of reduced PCYT2 activity in metabolic diseases.

The Pcyt2 heterozygous *Pcyt2*^+*/−*^ mouse is an attractive model to study the contribution of membrane phospholipid metabolism to NASH as it exhibits similar features to humans, importantly, the association of gradual disease onset and decreased PE synthesis with age^17–19^, since early disease pathologies are not well established.

We have shown that complete deletion of *Pcyt2* gene in mice is embryonically lethal^20^ and that heterozygous *Pcyt2*^+*/−*^ show reduced PE turnover, and increased DAG and TAG accumulation leading to the development of obesity and insulin resistance at an older age^13,20,21^. Decreased PE content associates with age in the brain and mitochondria of liver and muscle^17,18^, and TAG accumulation in muscle^18^, suggesting the potential for age-related alterations in Pcyt2 activity and PE homeostasis. Previous mouse models used to study NASH have induced the disease via choline/methionine deficient and high-fat diets, however these models exhibit meaningful differences in their etiology and pathogenic changes^22^, whereas *Pcyt2*^+*/−*^ develop NASH on a normal diet. Moreover, *Pcyt2*^+*/−*^ mice are a unique model for representing NASH in humans because, unlike previous models^23,24^, they exhibit both histological and inflammatory features of NASH as well as associated metabolic physiology^19,20^. In this study, we characterize the age-dependent development of liver defects with a focus on the main pathways that contribute to the development and sustainment of *Pcyt2*^+*/−*^ NASH. We further hypothesize that supplementation with the artificial Pcyt2 substrate,phosphonoethylamine (PEA), will reverse the liver pathological state in *Pcyt2*^+*/−*^. PEA is a phosphonate analog of PEtn (the natural Pcyt2 substrate), in which the phosphorus group is linked by a C-P bond. PEA is a biogenic compound that is present in a range of organisms, including human and other mammalian tissues^25–27^. Multiple metabolism studies have shown PEA to be incorporated through the Kennedy pathway into the phosphonate analog of PE, including in the rat liver, showing PEA as a substrate for Pcyt2^25,26^. Therefore, we hypothesize that PEA supplementation will stimulate flux through the depressed CDP-Etn Kennedy pathway to ameliorate the consequences of Pcyt2 deficiency.

## Methods

### Animals and treatments

Heterozygous *Pcyt2* mice (*Pcyt2*^+*/−*^) were generated and genotyped as previously described^20^. All procedures were approved by the University of Guelph's Animal Care Committee and were in accordance with guidelines of the Canadian Council on Animal Care (CCAC). We also followed the ARRIVE guidelines for reporting results. Mice were housed in a temperature-controlled facticity and exposed to a 12 h light/12 h dark cycle beginning with light at 7:00 a.m. Mice were fed a standardized chow diet (Harlan Teklad S-2335) and had free access to water. Mice that were supplemented with PEA (PEA, Sigma-Aldrich 268674) were done so through free access to water containing 1 mg/mL of PEA. Dosage of PEA was calculated based on physiological levels of Etn (10–75 µM)^28^ and previously determined water intake^29^. Wild type littermates (*Pcyt2*^+*/*+^) and *Pcyt2*^+*/−*^ mice (n = 6–10) were euthanized under both fasted and fed conditions at 2 and 6–8 mo. *Pcyt2*^+*/−*^ mice that were supplemented with PEA (*Pcyt2*^+*/−*^ + PEA) were euthanized under fed conditions at 6–8 mo (n = 6–12 per group). The treatment period lasted 8 weeks. No differences were observed between age-matched males and females and thus, both sexes were used for final analysis.

### Immunoblotting

Liver samples (~ 150 mg; n = 3–4 per group) were immediately frozen in liquid nitrogen and stored at −80 °C. Frozen tissues were homogenized in 50 mM HEPES [pH 7.4], 1% TritonX-100, 50 mM sodium pyrophosphate, 0.1 M sodium fluoride, 10 mM EDTA, 10 mM sodium orthovanadate, 10 μg/ml aprotinin, 10 μg/ml leupeptin, 2 mM benzamidine and 2 mM PMSF using a polytron homogenizer. The protein content was determined using BCA protein assay kit (Pierce). Proteins were resolved on a 5% and 10% denaturing SDS-PAGE gels and semi-dry transferred to PVDF membranes. Following transfer, proteins were visualized using Ponceau S staining to ensure proper transfer and equal loading. Membranes from Figure XXX4 (Pcyt2α, Ctl1), Figure XXX5 (Pkcα, Pkcβ1, p-Foxo1, β-Actin, Sirt1, Pgc1α, pAmpkα, Ampkα, p-p70 S6K, mTorc1) and Figure XXX6 (Traf6, NfκB, NfκB nuclear, Stat3, Stat3 nuclear, Keap1, β-Tubulin, Nrf2, Nrf2 nuclear, p-Eif2α, Eif2α, β-Actin, p-Erk1/2, Erk1/2, p-Jnk1/2, Jnk1/2, p-p38, p38) were cut prior to antibody hybridization to allow for the probing of multiple targets on one membrane. All antibodies used have been previously published and validated in our lab and are found to not have non-specific binding. Membranes were blocked at room temperature for 2 h in 5% BSA in TBS-T followed by incubation with primary antibodies Ir, Irs1, p-Tyr-Irs1 (Millipore); Pi3k p85, Akt1, Akt2, pThr^308^-Akt, pSer^473^-Akt, Ampkα, p-Ampkα, p-Acc, Acc, p-p70S6K, mTorc1, p-Pka substrates, Sirt1, p-Foxo1, Pgc1α Stat3, Nf-kB p65, Keap1, Pkcα, Socs3, Nrf2, pSer^51^-Eif2α, Eif2α, Traf6, p-p38 Mapk, p38 Mapk, p-Erk1/2, Erk1/2, p-Jnk1/2, Jnk1/2, β-Tubulin, β-Actin (Cell signaling); Pckβ1, Pckβ2 (Santa Cruz), Srebp1c, Angptl4 (Invitrogen) at a 1:1000 dilution in %5 BSA at 4 °C overnight. Ctl1 (ENS-627)^30^ and Pcyt2α (V-5407)^20^ were produced in our laboratory and incubated at a 1:100 and 1:2000 dilution in 5% BSA, respectively, at 4 °C overnight. Membranes were washed 3 × in TBS-T and incubated with the appropriate horseradish peroxidase conjugated secondary antibody (1:10,000) in 5% BSA in TBS-T for 1 h at room temperature and visualized using chemiluminescent substrate (Sigma). β-Tubulin, β-Actin or Ponceau S were used as loading controls. The intensity of specific bands was quantified using NIH ImageJ software.

### Immunoprecipitation

Liver homogenates (n = 4) were thawed on ice prior to being pre-cleared with protein G agarose beads (Promega) for 2 h at 4 °C. Liver protein of 125 μg was pre-cleared with 25 μl of agarose beads. Supernatants were thereafter removed and incubated with a 1:100 dilution of specific antibodies overnight at 4 °C. Following the overnight incubation at 4 °C, 30 and 60 μl of agarose beads were added to the sample. All samples were incubated for 2 h at 4 °C and centrifuged at 4 °C for 10 min at 14,000 rpm. Supernatants were discarded and beads were washed 4 times for 30 min each with PBS-T at 4 °C. Fifty μl of sodium dodecyl sulfate (SDS)-containing buffer (Tris–HCl, pH 6.8, dithiothreitol (DTT), 2% SDS, 0.1% phenol blue, 10% glycerol) was then added to beads, and samples were heated at 95 °C for 5 min. Following centrifugation for 10 min at 14,000 rpm, the supernatant was removed, divided into aliquots, and stored at −80 °C until immunoblotting experiments were conducted using IR, IRS-1, pTyrIRS1 (Millipore) and p85PI3K (Cell Signaling) antibodies.

### Gene expression analysis

Frozen liver tissue weighing between 50 and 100 mg (n = 3–4 per group), was homogenized in 1 mL of TRIzol Reagent (Thermo Scientific) according to the manufacturer’s protocol to isolate mRNA. cDNA was synthesized from 2 μg of total mRNA using a poly(dT) primer and Superscript III reverse transcriptase (Invitrogen). Expression of various genes involved in lipid metabolism were determined by polymerase chain reaction (PCR) using the primer sequences and conditions listed in Supplementary Table 1. PCR reactions were carried out using the following cycle parameters: 30 s at 94 °C, 30 s at respective melting temperatures (Tm) in Supplementary Table 1, and 30 s at 72 °C for 32 cycles. PCR products were resolved on a 1.5% agarose gel and quantified using ImageJ. Reactions were standardized to glyceraldehyde 3-phosphate dehydrogenase (GAPDH).

### Glucose and pyruvate tolerance tests

For glucose tolerance test (GTT), mice (n = 4 per group) were fasted for 6 h before intraperitoneal injection (I.P.) of 2 mg/kg of glucose in 0.9% saline. For pyruvate tolerance test (PTT), mice (n = 4 per group) were fasted overnight then administrated 2 g/kg of body weight of sodium pyruvate by I.P. injection. Blood glucose level was measured by glucometer immediately before injection and 30, 60 and 120 min after glucose injection and 15, 30, 60, 120 min after pyruvate injection.

### Hepatocyte glucose production

Primary hepatocytes were isolated as previously described^19^. Briefly, livers from 8-mo *Pcyt2*^+*/*+^ and *Pcyt2*^+*/−*^ mice were perfused with 0.5% collagenase and hepatocytes resuspended in PBS containing 0.5% BSA, 5 mM glucose and 3.3 mM pyruvate. Hepatocytes were plated on 6-well collagen-coated plates and allowed to attach for 2 to 4 h in Williams medium E. Floating cells were removed and media replaced with Williams medium E containing 10% FBS and 1% antibiotic–antimycotic solution. To measure glucose release, hepatocytes were incubated 3 h in a glucose-free DMEM (no phenol red, pH 7.4) supplemented with 20 mM sodium lactate and 2 mM sodium pyruvate. Glucose present in the media was determined with a glucose assay kit (Sigma).

### Glucokinase and glucose-6 phosphatase activities

Hepatic glucokinase (Gk) activity was measured as previously described^31^ with some modification. Liver samples (100 mg) were homogenized in 50 mM HEPES, 100 mM KCl, 1 mM EDTA, 5 mM MgCl_2_, and 2.5 mM dithioerythritol. Homogenates were briefly centrifuged and incubated for 10 min on ice with 25% PEG. The microsomal fraction/glucose 6-phosphatase activity was then removed by ultracentrifugation (100,000×g; 30 min; 4 °C). Glucose phosphorylating activities were measured by the production of NADPH from NADP^+^ in the presence of glucose 6-phosphate dehydrogenase (G6PDH) and either 100 mM glucose or 0.5 mM glucose, to distinguish hexokinase (Hk) from glucokinase (Gk) activity. The activity obtained at 0.5 mM glucose is considered the HK activity. The subtraction of the activity measured at 100 mM glucose from the activity measured at 0.5 mM glucose is considered the Gk activity.

The glucose-6 phosphatase (G6Pase) assay was based on the hydrolysis of glucose-6-phosphate to Pi by the microsomal fractions isolated above. The microsomal fractions were incubated with 10 mM glucose-6-phosphate at 37 °C, and the reaction was stopped after 20 min with acid molybdate containing 2/9 volume of 10% SDS and 1/9 volume iof 10% ascorbic acid. The mixture was then incubated for 20 min at 45 °C and the Pi-molybdate complex absorbance read at 820 nm. Protein concentration was measured using BCA assay (Thermo Fisher Sci).

### Glycogen content

The glycogen content was determined as previously described^32^. In brief, livers (50 mg; n = 12 per group) were immersed in 500 μl 30% potassium hydroxide saturated with sodium sulfate and boiled for 20–30 min until homogenous solution was obtained. Glycogen was precipitated with cold 95% ethanol, separated by centrifugation, and dissolved in distilled water. Glycogen content was determined at 490 nm after the addition of 5% phenol and 95% sulfuric acid. Standard curve was generated using pure glycogen (Roche).

### Liver histology and immunohistochemistry

Livers (n = 4 per group) were fixed in 10% formalin in PBS at room temperature for 12–16 h and embedded in paraffin until histopathologic examination. Sections were de-waxed in xylene and rehydrated in a series of ethanol washes. Sections of 10 μm were stained with hematoxylin and eosin (H&E) to examine lipid droplets, periodic acid-Schiff reagent (PAS) for glycogen, 0.1% Picrosirius red (Sigma) for collagen, and F4/80 antibody (Abcam) for macrophages and were visualized with light microscopy using standard techniques. All staining was performed at the Ontario Veterinary College, Department of Pathobiology, University of Guelph.

### Hepatic triglyceride content

Livers (200 mg; n = 9 per group) were homogenized in 500 μL of PBS and 5% Tween 20. Samples were heated for 5 min at 95ºC and cooled to room temperature. Heating/cooling process was repeated, and insoluble material was removed through centrifugation. A TAG assay kit (Wako Diagnostics 994–02891 and 998–02992) was used to quantify TAG content.

### 2-Bromopalmitate and 2-deoxyglucose uptake by liver

Mice were fasted for 12 h (n = 6 per group) and retro-orbitally injected with 5 μCi 2-deoxy[^14^C]glucose and 1.5 μCi BSA-complexed 2-bromo-[^3^H]palmitate. After 5 min post injection, blood was drawn and radioactivity in serum determined by liquid scintillation counting (LSC). After 1 h post injection, livers were harvested and homogenized in PBS. Radioactivity was determined by LCS and calculated as a portion of the initial radioactivity present in 5 μl of serum 5 min after injection, to adjust for the injected dose.

### RT-PCR array

Liver RNA from *Pcyt2*^+*/*+^ and *Pcyt2*^+*/−*^ (n = 3 for each group) was extracted using the RNeasy Mini Kit (QIAGEN). The samples were pooled together for complementary DNA synthesis, 1 µg total RNA was reverse transcribed using the High-Capacity RNA-to DNA Master Mix (Applied Biosystems). Complementary DNA, equivalent to 40 ng RNA, was used as a template for real-time reverse-transcription PCR (RT-PCR) using an Applied Biosystems 7900HT Fast Real-time PCR system (Applied Biosystems). Insulin Signaling Pathway PCR Array Mouse Gene Expression Array—PAMM-030Z (SA-Biosciences) was used for expression of 84 genes. PCR array data were calculated by the comparative cycle threshold method, normalized against multiple housekeeping genes, and expressed as mean fold change.

The enrichment analysis tool Enrichr (https://maayanlab.cloud/Enrichr/#) was used for the analysis of the microarray data (GEO microarrays GSE55617) and RT-PCR array data^33–35^. The bar charts of the top 10 enriched terms from the selected libraries and clustergrams of the input genes vs. the enriched terms were produced separately for upregulated and down-regulated genes. The Manhattan and Volcano plots that establish the significance of each gene set vs. its odds ratio were visualized with Appyter (https://appyters.maayanlab.cloud/#/Enrichment_Analysis_Visualizer).

### Blood biochemistry

For analysis of liver enzymes and albumin (n = 12), blood was collected after 12 h of fasting. Serum was separated immediately through centrifugation and sent to Animal Health Laboratory (University of Guelph) for biochemical analyses.

### Statistical analysis

Data was analyzed using two-tailed unpaired t-test, and for differences between more than 2 groups one-way ANOVA with Tukey's post hoc test was performed. Significance was rejected at *p* ≥ 0.05. Results are represented as mean ± SD. All statistical tests were performed with Graphpad Prism 6 software.

## Results

### Liver glucose contributes to increased glucose tolerance in older ***Pcyt2***^+***/−***^

**Figure 1:**
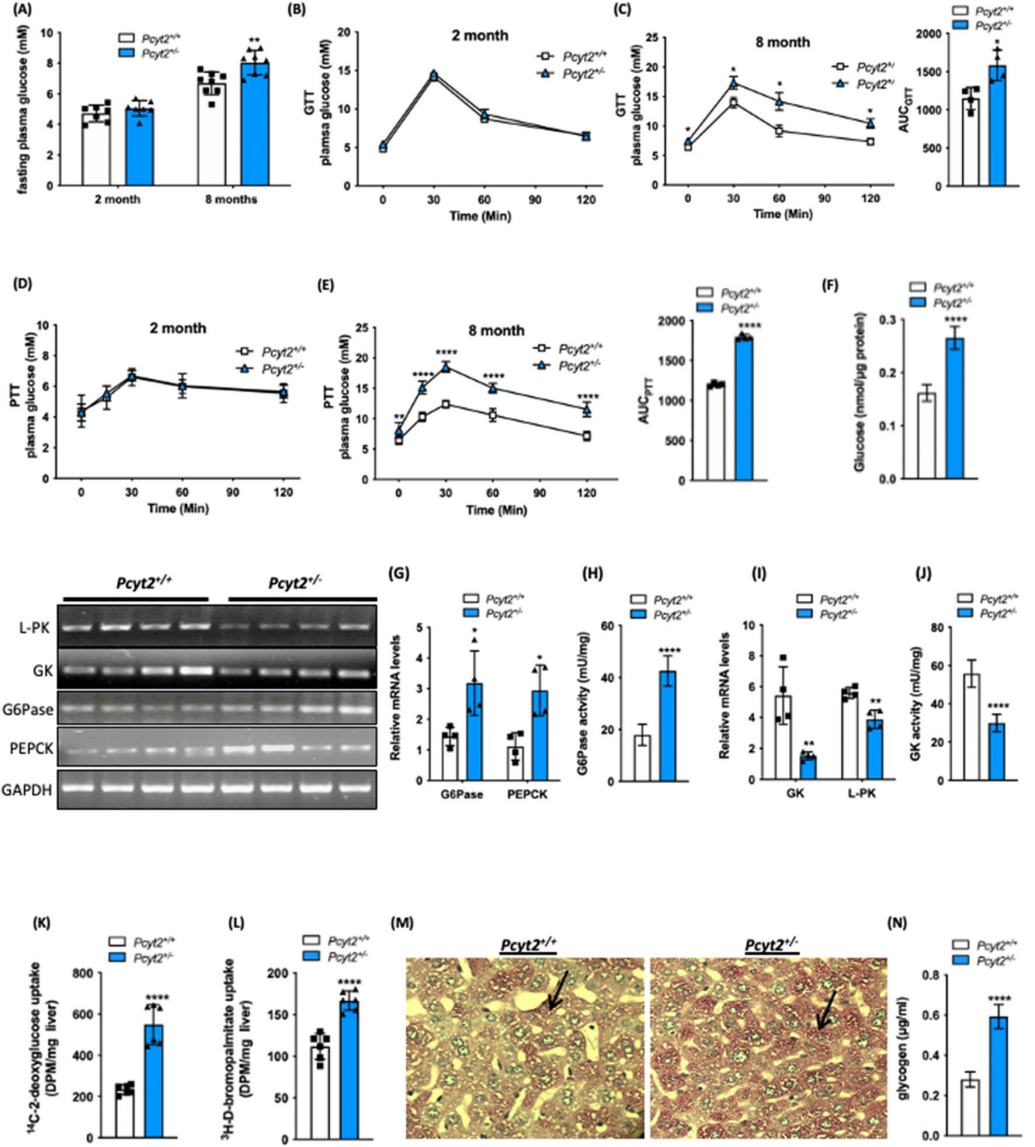
Glucose metabolism is altered in older *Pcyt2*^+*/−*^ mice. (**A**) Fasting glucose levels for 2-mo and 8-mo *Pcyt2*^+*/*+^ and *Pcyt2*^+*/−*^ mice. (**B**) Intraperitoneal glucose tolerance test for 2-mo and (**C**) 8-mo *Pcyt2*^+*/*+^ and *Pcyt2*^+*/−*^ and area under the curve (n = 4). (**D**) Intraperitoneal pyruvate tolerance test for 2-mo and (**E**) 8-mo *Pcyt2*^+*/*+^ and *Pcyt2*^+*/−*^ and area under the curve (n = 4). (**F**) Glucose production in primary hepatocytes (n = 12). (**G**) Relative mRNA expression levels of gluconeogenic enzymes *G6Pase* and *Pepck* (n = 4) and (**H**) G6Pase enzymatic activity (n = 12) in 8-mo *Pcyt2*^+*/*+^and *Pcyt2*^+*/−*^ mice. (**I**) Relative mRNA expression levels of glycolytic enzymes *Gk* and *L-Pk* (n = 4) and (**J**) GK enzymatic activity (n = 12) in 8-mo *Pcyt2*^+*/*+^ and *Pcyt2*^+*/−*^ mice. (**K**) [^14^C]deoxyglucose and (**L**) [^3^H]bromopalmitate uptake in liver (n = 6). (**M**) Periodic acid-Schiff staining of liver sections for glycogen in 8-mo *Pcyt2*^+*/*+^ and *Pcyt2*^+*/−*^ mice. (**N**) Liver glycogen content of 8-mo *Pcyt2*^+*/*+^ and *Pcyt2*^+*/−*^ mice (n = 12). Band intensities were measured using ImageJ. Data are presented as mean ± SD. *p < 0.05; **p < 0.01; ***p < 0.001; ****p < 0.0001.

*Pcyt2*^+*/−*^ mice are similar weights at 2-mo compared to control littermates but progressively gain more weight than controls as they age despite consuming equal amounts of food^21^. To help elucidate the mechanism by which heterozygous ablation of *Pcyt2* affects glucose metabolism by age, we performed glucose (GTT) and pyruvate (PTT) and tolerance tests on 2-mo and 8-mo mice. *Pcyt2*^+*/−*^ fasting glucose levels are unaltered at 2-mo of age, but by 8-mo are 20% higher than age-matched *Pcyt2*^+*/*+^ littermates (Fig. 1A). Two-mo *Pcyt2*^+*/−*^ maintain normal glucose levels in response to the GTT while 8-mo *Pcyt2*^+*/−*^ are hyperglycemic compared to *Pcyt2*^+*/*+^ littermates (Fig. 1B and C). GTT area under the curve (AUC) was elevated by 38% in 8-mo *Pcyt2*^+*/−*^ showing age-dependent and reduced glucose clearance from plasma (Fig. 1C). This adds to our previous findings of elevated insulin levels in response to a glucose challenge in 9-mo but not 2-mo *Pcyt2*^+*/−*^^21^, showing that impaired glucose metabolism is a consequence, not a cause of the *Pcyt2*^+*/−*^ phenotype.

We determined the ability of the *Pcyt2*^+*/−*^ liver to utilize pyruvate for glucose production through an intraperitoneal injection of sodium pyruvate and measurement of the subsequent rise in plasma glucose levels. Glucose production is unchanged in 2-mo *Pcyt2*^+*/−*^ but increased in 8-mo *Pcyt2*^+*/−*^ with a 50% elevation in PTT AUC, relative to *Pcyt2*^+*/*+^ littermates (Fig. 1D and E). To reinforce the concept that Pcyt2 deficiency augments liver glucose production we determined glucose release from primary hepatocytes isolated from 8-mo mice. Indeed, primary *Pcyt2*^+*/−*^ hepatocytes exhibit a 64% increased glucose output compared to *Pcyt2*^+*/*+^ hepatocytes (Fig. 1F). This completements our previous evidence showing increased formation of both DAG and TAG that could be normalized with overexpression of Pcyt2 complementary DNA in Pcyt2 deficient primary hepatocytes^13^, establishing perturbed glucose and lipid homeostasis in *Pcyt2*^+*/−*^ primary hepatocytes is a result of Pcyt2 deficiency.

Further indicators of elevated glucose production are shown in the increased expression of the key liver enzymes in the gluconeogenic pathway. In fasted 8-mo *Pcyt2*^+*/−*^ mice, mRNA levels of *Pepck* and *G6Pase* are increased by 2.36- and 2.21-fold, respectively, along with a 2.37-fold increase in G6Pase enzyme activity (Fig. 1G and H). The expression of glycolytic *L-Pk* was modestly reduced by 31% but *Gk* expression was reduced 3.58-fold and Gk activity by 46% showing reduced glucose utilization by glycolysis in old *Pcyt2*^+*/−*^ liver (Fig. 1I and J). Together these data show that liver glucose production by gluconeogenesis was normal at younger age, however, significantly increased and contributed to the elevated plasma glucose in older *Pcyt2*^+*/−*^.

In addition, in vivo radiolabeling experiments showed that the incorporation of the undegradable [^14^C]deoxyglucose is increased 2.36-fold in fasted 8-mo *Pcyt2*^+*/−*^ liver relative to *Pcyt2*^+*/*+^ littermates. The FA uptake measured by the metabolically stable [^3^H]bromopalmitate is increased by 49% demonstrating that both glucose and FA are more readily available in fasted *Pcyt2*^+*/−*^ than in fasted *Pcyt2*^+*/*+^ mice (Fig. 1K and L). Liver staining displayed altered glycogen storage and quantitative analysis revealed a 75% increase in glycogen content in older *Pcyt2*^+*/−*^ liver (Fig. 1M and N).

### Enrichment gene analysis of young ***Pcyt2***^+***/−***^ predict liver disease

**Figure 2:**
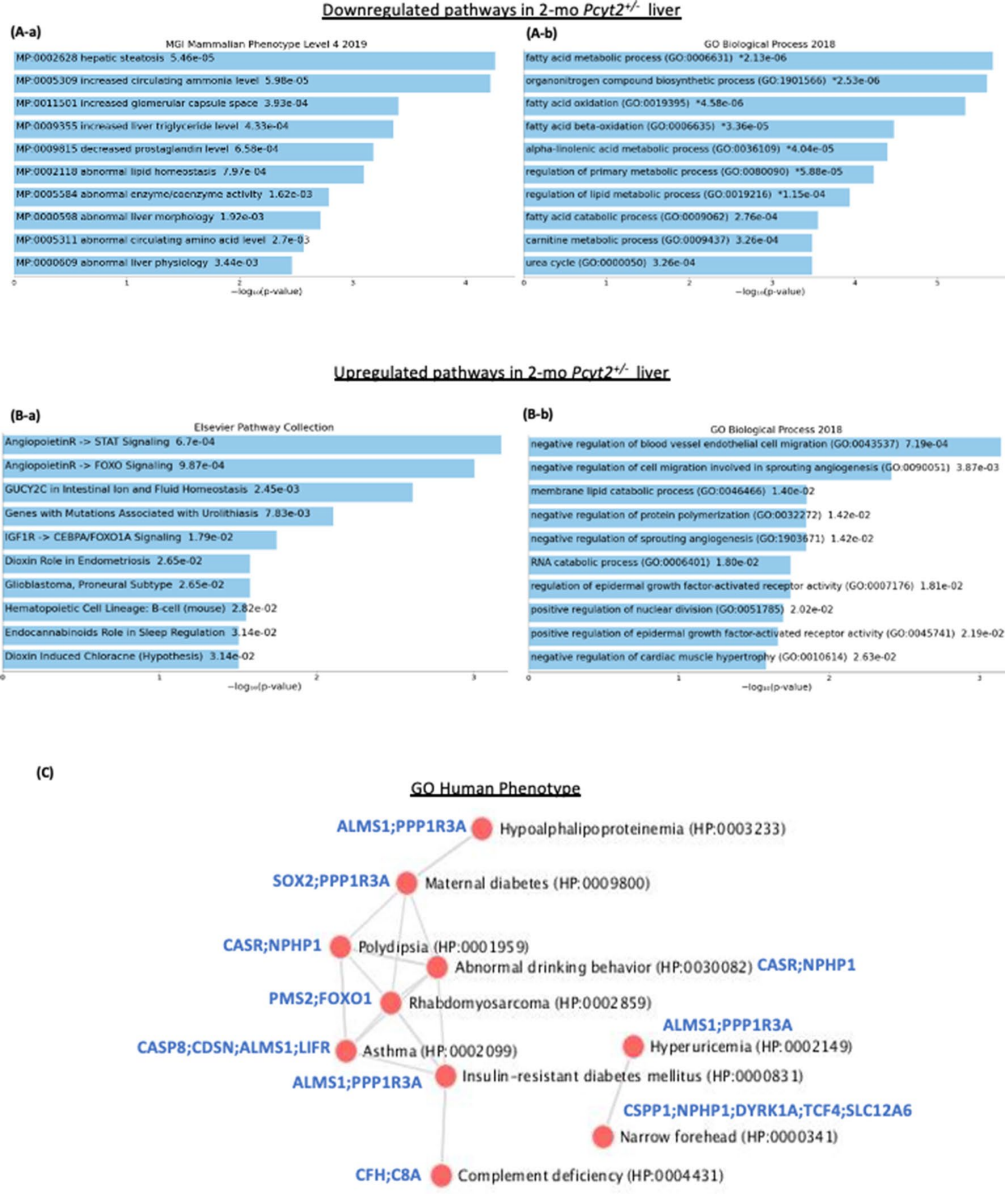

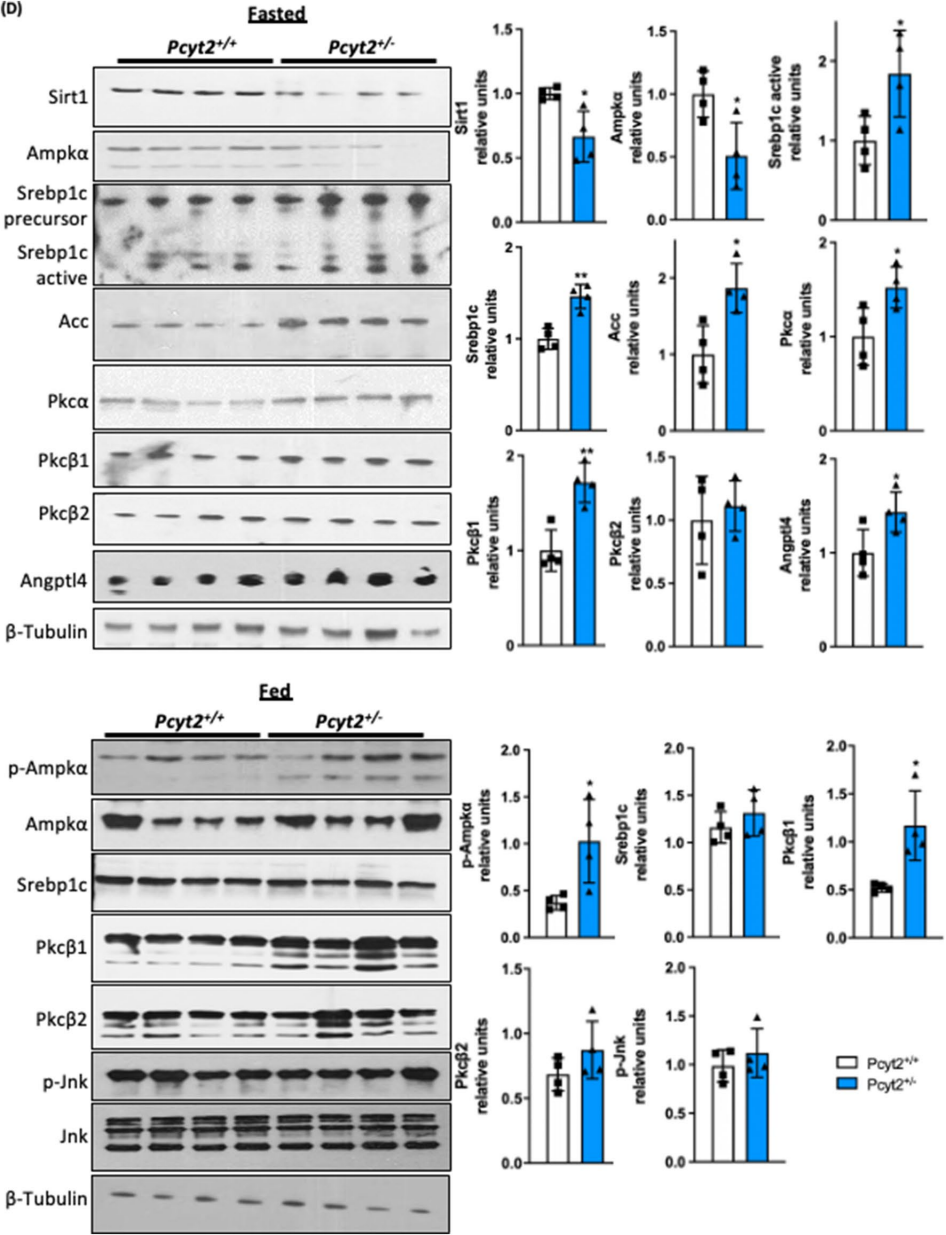
Analysis of down- and upregulated pathways in 2-mo *Pcyt2*^+*/−*^ liver. (**A**) Downregulated genes from 2-mo *Pcyt2*^+*/−*^ liver were analyzed with Enrichr (https://maayanlab.cloud/Enrichr/)^33–35^ (n = 3). (**A-a**) Pathway analysis (MGI Mammalian Phenotypes Level 4–2019) established the most enriched terms include hepatic steatosis and increased circulating ammonia. (**A-b**) The Gene Onthology (GO) analysis (GO Biological Processes 2018) further shows that the most significantly downregulated were processes of fatty acid oxidation and nitrogen degradation. The most frequently downregulated GO genes from phospholipids and fatty acid metabolism and nitrogen (urea cycle, arginine) are indicated in the clustergram. (**B**) Upregulated genes from 2-mo *Pcyt2*^+*/−*^ liver were analyzed with Enrich (n = 3). (**B-a**) The cluster analysis for the Elsevier Pathway Collection identified as elevated the Igf2- and Ang4/Ang2-Foxo1 pathways and ion/amino acid transport. (**B-b**) GO Biological Processes 2018 of the most upregulated genes identified as the most important the processes linked to Ang2/Ang4 functions in regulation of angiogenesis and Egfr signaling. (**C**) Go:Human Phenotype establish a gene/disease network for young *Pcyt2*^+*/−*^ with significant risk for development of maternal- and type 2- diabetes and related pathologies. (**D**) Immunoblot analysis of lipid pathways in fasted and fed *Pcyt2*^+*/*+^ and *Pcyt2*^+*/−*^ (n = 4). Band intensities were measured using ImageJ. Data are presented as mean ± SD. *p < 0.05; **p < 0.01.

To determine early changes in gene expression caused by Pcyt2 deficiency we assessed the existing microarray data for 2-mo *Pcyt2*^+*/−*^ liver (GEO microarray data set: GSE55617) (Fig. 2A and B, Supplementary Tables 2 and 3). At young age *Pcyt2*^+*/−*^ has no clinical symptoms of steatosis or insulin resistance (Fig. 1), yet the pathway analysis (Fig. 2A-a, Supplementary Table 2) of the 2-mo *Pcyt2*^+*/−*^ downregulated genes (714 genes at *p* < 0.05) established that the most enriched pathways are for hepatic steatosis, abnormal liver physiology/morphology, increased triglyceride and ammonia, and abnormal amino-acid levels. The Gene Onthology (GO) analysis (Fig. 2A-b, Supplementary Table 2) further indicated that the most downregulated processes were synthesis and oxidation of fatty acids (gene cluster in Fig. 2A-b: *Acox1, Aldh3a2, Pparγ, Acad11, Adipor2, Acadm, Acadl, Cpt1a, Elovl5, Acsl, Ehhadh, Decr2*), CDP-Etn Kennedy pathway (*Pcyt2, Cept*) and nitrogen metabolism (nitric oxide and urea cycle genes: *Nos3, Arg1, Ass1, Asl*). As expected, because of *Pcyt2* single-allele deletion, *Pcyt2* was among those downregulated genes in 2-mo *Pcyt2*^+*/−*^ (Fig. 2A-b). Insulin signaling and genes involved in glucose metabolism were not significantly downregulated in 2-mo *Pcyt2*^+*/−*^.

The enrichment analysis of 534 upregulated genes established multiple modified pathways in 2-mo *Pcyt2*^+*/−*^ liver (Fig. 2B, Supplementary Table 3). Significantly enriched pathways include the angiopoietin signaling (*Angpt4, Angpt2, Foxo1, Lck*), endocannabinoid PE related pathway (*Napepld, Daglb*) and response to hypoxia (*Arnt, Epgn*) (Fig. 2B-a). The pathway analysis with BioPlanet2019 and Reactome2016 both recognized as the most significant the transport processes of inorganic cations/anions and amino acids (*Slc9a3, Slc6a19, Slc7a8, Slc34a1, Slc7a11, Slc12a6*), G-protein activation (*Gnaz, Gnb4, Gng12*) and Caspase 8 activation/apoptosis (*Casp8, Tnfsf10, Madd*) (Supplementary Table 3). In agreement with this analysis, Gene Ontology (GO: Biological processes 2018) analysis (Fig. 2B-b, Supplementary Table 3) further indicated that angiopoietins as well as thromboxane signaling, (*Angpt4, Angpt2, Tbxa2r, Mmrn2, Meox2*) are the most important for the negative regulation (disruption) of angiogenesis. Interestingly, GO Human Phenotype analysis of the upregulated genes in 2-mo *Pcyt2*^+*/−*^ liver (Fig. 2C) identified a disease network between Insulin-resistant Diabetes and Maternal Diabetes and Hypoalphalipoproteinemia (HDL lipoprotein deficiency), Hyperuricemia, Polydipsia**,** Rhabdomyosarcoma and immunity (Asthma and Complement deficiency). The muscle specific glycogen-associated regulatory subunit of Protein Phosphatase-1 (*Ppp1r3a*) was the most common gene in this new network. *Ppp1r3a* plays a critical role in glycogen synthesis that is independent of insulin^36^.

### Young ***Pcyt2***^+***/−***^ exhibit defects in fatty acid metabolism that remain impaired with aging

Because young *Pcyt2*^+*/−*^ had normal GTT and PTT tests (Fig. 1B and D) yet the microarray analysis indicated an early impairment in fatty acid metabolism (Fig. 2A) we next checked the activity of mitochondrial and fatty acid metabolic pathways. Fasted 2-mo *Pcyt2*^+*/−*^ showed reduced levels and activity of the mitochondrial activators Sirt1 and Ampkα, 34% and 49%. Lipogenic ACC, and Srepb1c both in the precursor and active form were increased by 87%, 46% and 84%, respectively. Pkcα and Pkcβ1/2, well known DAG regulated kinases, increased by 52% and 72% while Pkcβ2 underwent a modest increase of 11%. In addition, the important angiogenic factor and inhibitor of lipolysis Angptl4 exhibited a 43% elevation in 2-mo *Pcyt2*^+*/−*^ (Fig. 2D). Importantly, Angpt4 and Angpt2 gene expression and signaling via STAT and FOXO1 pathways were also upregulated in 2-mo *Pcyt2*^+*/−*^ (Fig. 2B, Supplementary Table 3). In the fed state, only p-AMPK and Pkcβ1 were increased by 177% and 144%.

To determine if these defects persist into adulthood, we measured these proteins in fasted 6-mo *Pcyt2*^+*/−*^ (Supplementary Fig. 1). Indeed, Sirt1 is reduced by 31%, total Ampkα and p-Ampkα: Ampkα ratio decreased 56% and 43% and highly increased Srebp1c in both the precursor form (2.95-fold) and the active form (2.40-fold), along with a substantial (6.40-fold) increase in Acc. DAG regulated Pkcα and Pkcβ1/2, are also drastically elevated by 3.25-fold, 4.87- fold and 7.45-fold. Stat and Foxo1 pathways also continued to be modified with aging in 6-mo old *Pcyt2*^+*/−*^ as it will be shown later as part of the PEA study.

Together these data show that an early reduction in mitochondria energy production and increased fatty acid synthesis by lipogenesis^37^ preceded development of adult *Pcyt2*^+*/−*^ liver steatosis, and became even more impaired with ageing.

### Enrichment gene analysis of older ***Pcyt2***^+***/−***^ reflect adult disease phenotype

**Figure 3:**
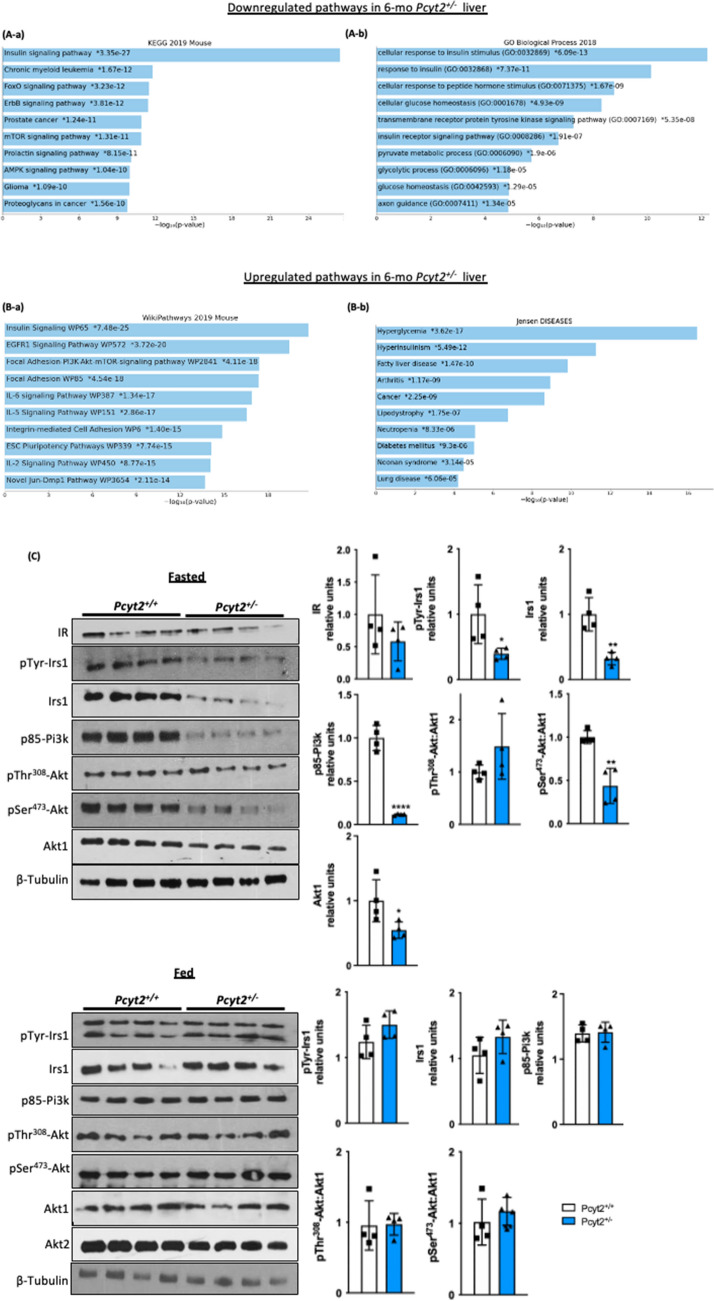

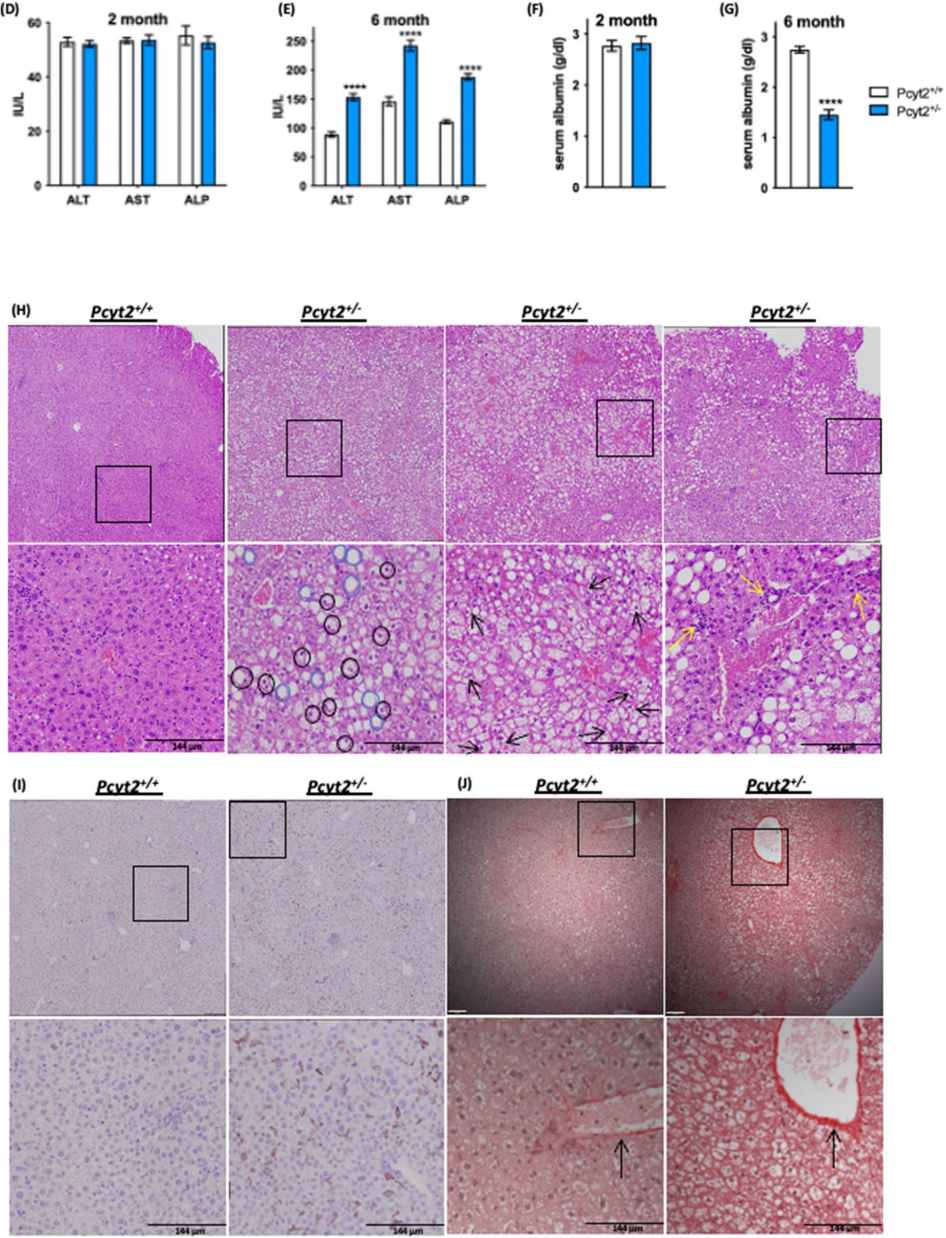
Analysis of down- and upregulated pathways in 6-mo *Pcyt2*^+*/—*^liver*.* (**A**) Downregulated genes of 6-mo *Pcyt2*^+*/−*^ liver were analyzed with Enrichr (https://maayanlab.cloud/Enrichr/)^33–35^ (n = 3). (**A-a**) The Mouse 2019 KEGG^38–40^ pathway analysis and genes cluster analysis indicated that the most downregulated pathways included the growth factor (insulin, ErB, prolactin, cancer) pathways, and mTOR and AMPK pathways. (**A-b**) GO Biological Processes 2018 showed that the most downregulated processes were insulin signaling and glucose metabolism. (**B**) Upregulated genes of 6-mo old *Pcyt2*^+*/−*^ were analyzed with Enrich (n = 3). (**B-a**) WikiPathway Mouse 2019 established the most significantly upregulated gene clusters and pathways for insulin/EGFR signaling and pro inflammatory pathways mediated by IL2, IL9, IL5, IL6, and IL7 (**B-b**) Jansen Diseases analysis showed that the most upregulated were gene involved in hyperglycemia, hyperinsulinemia, and fatty liver disease. (**C**) Immunoblot analysis of Pi3k/Akt pathway in fasted and fed 6-mo *Pcyt2*^+*/−*^ (n = 4). Serum levels of liver enzymes ALT, AST, ALP in (**D**) 2-mo and (**E**) 6-mo *Pcyt2*^+*/−*^ (n = 12). Serum levels of albumin in (**F**) 2-mo and (**E**) 6-mo *Pcyt2*^+*/−*^ (n = 12). Histology of 8-mo liver with (**H**) H&E stain showing steatosis (blue circles), ballooned hepatocytes (black circles) with Mallory-Denk bodies (black arrows) and lobular inflammation (yellow arrows); (**I**) immunohistochemical stain with F4/80 showing macrophage infiltration; (**J**) Picrosirius red stain showing collagen deposition. Band intensities were measured using ImageJ. Data are presented as mean ± SD. *p < 0.05, **p < 0.01, ***p < 0.001, ****p < 0.0001.

Enrichment analysis of RT-PCR arrays for 6-mo *Pcyt2*^+*/−*^ liver indicated that insulin signaling, FoxO, mTOR, AMPK and several growth factors that share post-receptor regulation with insulin (cluster: *Mapk2k1, Sos1, Raf, Pi3kca, Pi3kr2, Braf*) were downregulated (Fig. 3A-a, Supplementary Table 4). Gene ontology (GO Biological processes 2018) analysis established that the most significantly downregulated genes were responses to insulin/peptide hormones/insulin receptor/tyrosine kinase signaling and pyruvate/glycolytic process/glucose homeostasis (Fig. 3A-b, Supplementary Table 4 gene list and statistics for top 10 processes).The upregulated pathways in 6-mo *Pcyt2*^+*/−*^ (Fig. 3B-a, Supplementary Table 5) included growth-promoting pathways (insulin, EGFR1, Jun), stem cell pluripotency, cell adhesion (focal/ integrin cell adhesion), as well as proinflammatory pathways (IL-6, IL-5, IL-2). These pathways share a large set of genes participating in receptor activation, cell signaling and nuclear transcription (*Shc1, Grb2, Akt1, Pik3r1, Raf1, Mapk, Pik3r, Fos, Jun, Araf, Kras*). In addition, Jensen Disease analysis (Fig. 3B-b, Supplementary Table 5) establish that the upregulated genes are involved in Hyperglycemia, Hyperinsulinemia, Fatty liver disease, Type-2 diabetes, Lipodystrophy, Arthritis, Neutropenia, Cancer, Lung disease and Noonan syndrome. The most shared upregulated genes in 6-mo *Pcyt2*^+*/−*^ liver were the lipogenic genes (*Srebp1, Pparγ, Retn, Lep*), the glucose metabolic genes (*Slc2a1, G6pc, Irs2, Akt1*) and the growth promoting genes (*Kras, Jun, Vegfa*).

### Older ***Pcyt2***^+***/−***^ liver develops impaired Pi3k/Akt signalling and steatohepatitis.

We next examined the activity of the insulin signaling pathway in 6-mo *Pcyt2*^+*/−*^ liver. Consistent with the microarray data, older *Pcyt2*^+*/−*^ shows severe impairment in the Irs1/Pi3K/Akt pathway in fasted state (Fig. 3C). Insulin receptor IR was not significantly modified, however, total Irs1 protein was reduced by 68% and pTyr-Irs1 was diminished by 60%. Pcyt2 deficiency caused a dramatic 88% reduction in p85-Pi3k and a 45% decrease in Akt1. Pdk mediated activation at pThr^308^-Akt is not affected however mTorc2 meditated pSer^473^-Akt activation was diminished by 56%. *Pcyt2*^+*/−*^ liver did not show impairments in the insulin signaling in fed state where phosphorylated and total content of Irs1, p85PI3K and Akt1/2 were unchanged.

Based on the pathway analysis indicating an upregulation of genes involved in lipogenesis and fatty liver disease and proinflammatory pathways, we examined serum biomarkers of liver disease/dysfunction and the primary histological features of NASH. We have previously shown that 2-mo *Pcyt2*^+*/−*^ have normal plasma TAG levels but by 8-mo *Pcyt2*^+*/−*^ plasma TAG content is elevated due to an age-dependent upregulation of very low density lipoprotein (VLDL) secretion and liver microsomal triglyceride transfer protein activity^21,37^. Hepatic enzymes alkaline phosphatase (ALP), alanine aminotransferase (ALT) and aspartate aminotransferase (AST), are normal at 2-mo, however, 6-mo *Pcyt2*^**+/−**^ exhibit elevations in ALP, ALT and AST by 70%, 72% and 66%, respectively (Fig. 3D and E). Serum albumin level is unaffected in 2-mo *Pcyt2*^**+/−**^ but is reduced by 50% 6-mo *Pcyt2*^**+/−**^ (Fig. 3F and G).

H&E staining revealed adult *Pcyt2*^**+/−**^ develop steatosis (blue circles), ballooned hepatocytes (black circles) with Mallory-Denk bodies (black arrows) and lobular inflammation (yellow arrows) (Fig. 3H). Immunohistochemical staining with F4/80 revealed macrophage infiltration (Fig. 3I) and picrosirius red staining revealed increased collagen deposition (Fig. 3J) in older *Pcyt2*^+*/−*^ liver. Together, these data indicate hepatocellular damage and inflammation that is consistent with progressive liver functional impairment associated with NASH.

### PEA modifies phospholipid and fatty acid metabolic genes and cell signaling proteins

**Figure 4:**
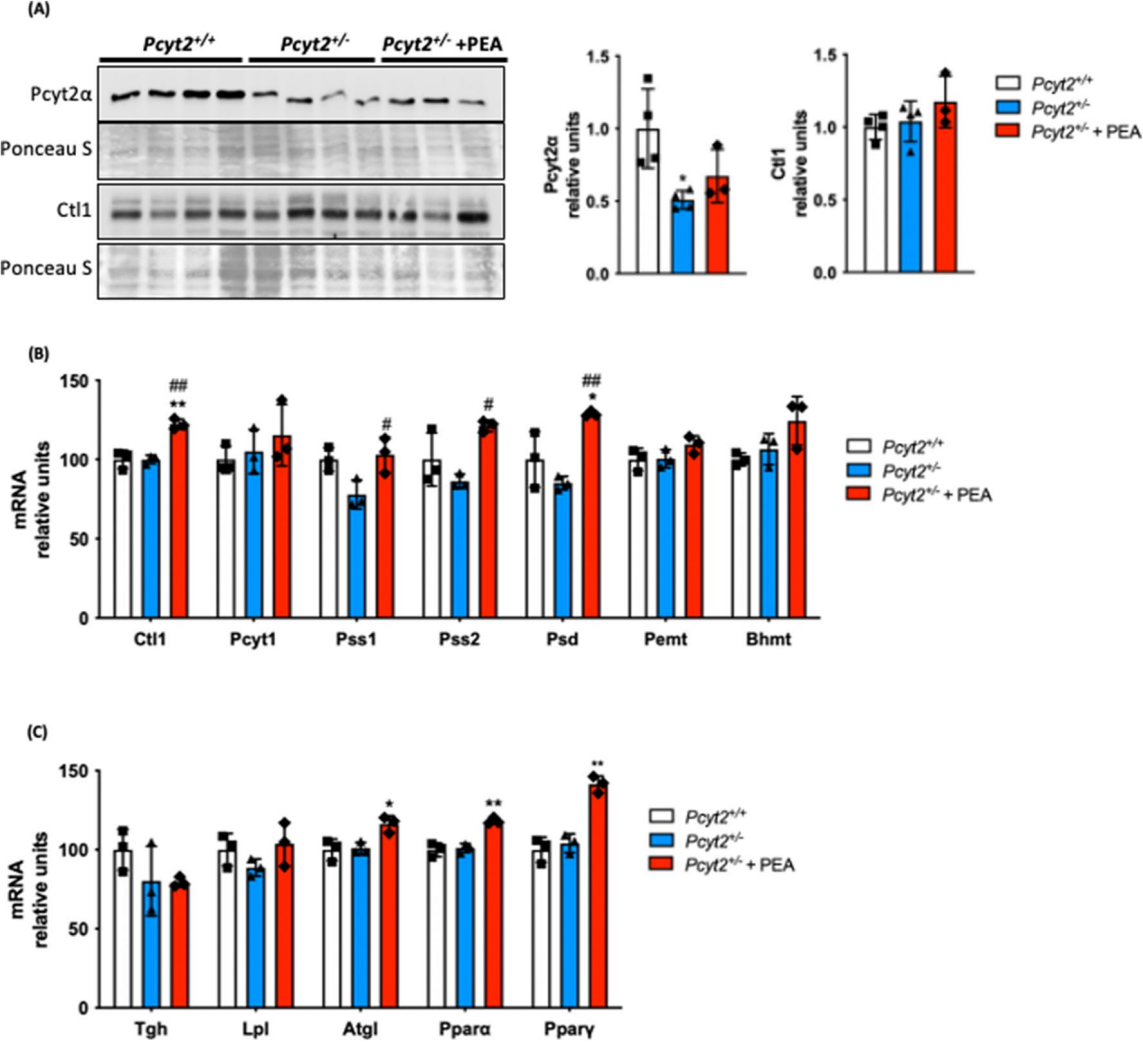
PEA stimulates phospholipid and triglyceride metabolic genes. (**A**) Immunoblot analysis of liver Pcyt2α and Ctl1 in 8-mo *Pcyt2*^+*/*+^, *Pcyt2*^+*/−*^ and *Pcyt2*^+*/−*^ + PEA mice treated for 12 weeks (n = 3–4). mRNA analysis of (**B**) phospholipid and (**C**) lipid metabolism genes in *Pcyt2*^+*/*+^, *Pcyt2*^+*/−*^ and *Pcyt2*^+*/−*^ + PEA mice. GAPDH used as loading control. Band intensities were measured using ImageJ. Membranes were cut prior to antibody hybridization to allow for the probing of multiple targets on one membrane. Data are presented as mean ± SD. *p < 0.05, **p < 0.01 relative to *Pcyt2*^+*/*+^; ^*#*^*p* < 0.05, ^##^*p* < 0.01 relative to *Pcyt2*^+*/−*^.

We next supplemented mice with the Pcyt2 substrate PEA at Etn physiological levels^28^ through drinking water and sacrificed mice at 8-mo as only older *Pcyt2*^+*/−*^ develop NASH. It was previously determined that supplementation does not influence water intake^29^. Because PEA was constantly supplemented, we evaluated the molecular effect of PEA in the fed state. As expected for single-allele deletion, in addition to the reduced mRNA (Fig. 2A-b), Pcyt2 protein was also reduced in *Pcyt2*^+*/−*^ and was unaltered with its PEA supplementation (Fig. 4A). The Kennedy pathway transporter Ctl1^41^ was increased by PEA at the mRNA level but protein content was not changed. PEA increased *Pss1* by 32% in *Pcyt2*^+*/−*^, suggesting that PS synthesis from PC readily occurs in *Pcyt2* deficiency. PEA also increased PS decarboxylase (*Psd*) and *Pss2* expression by 52% and 41%, respectively, indicating that PEA stimulated an increase in the conversions of PE to PS by Pss2 and Psd decarboxylation of PS to PE (Fig. 4B). Because Pss2 uses PE at the level of the ER, this indicates that PEA stimulation of the CDP-Etn Kennedy pathway was balanced by an increase in both Pss2 and Psd pathways, i.e., increased PE degradation to PS in the ER mitochondria associated membranes (MAM) by Pss2 occurs simultaneously with increased PS degradation to PE by Psd in the mitochondria. Taken together, such specific stimulatory effect of PEA on *Pss1*, *Pss2* and *Psd* genes that control the PC-PS-PE cycle showed that PEA was readily metabolized to PE by the CDP-Etn Kennedy pathway. In addition, PEA caused small but significant increase (15–17%) in the mRNA expression of the fatty acid and triglyceride metabolic regulators *Ppar ∝ *, *Pparγ* and *Atgl* (Fig. 4C).

**Figure 5:**
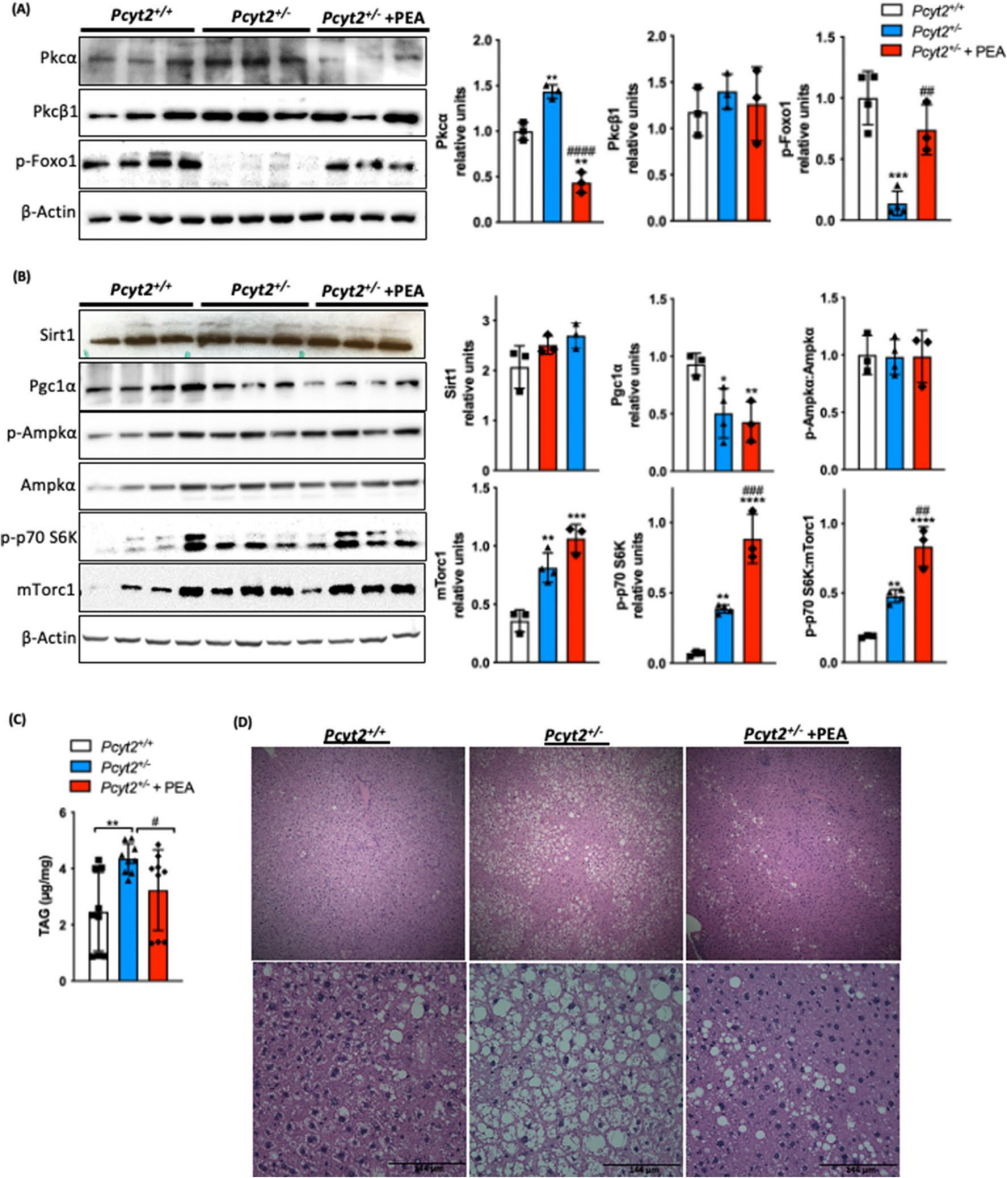
PEA inactivates Foxo1 and activates mTorc1 signaling pathway and reduced hepatic steatosis. Immunoblot analysis of liver (**A**) Pkcα, Pkcβ1, p-Foxo and (**B**) Sirt1, Pgc1α, p-Ampkα, Ampkα, p70S6K, and mTorc1 in 8-mo fed *Pcyt2*^+*/*+^, *Pcyt2*^+*/−*^ and *Pcyt2*^+*/−*^ + PEA mice (n = 3–5). (**C**) Liver triglyceride analysis (n = 9) and (**D**) H&E staining showing that 8-mo *Pcyt2*^+*/−*^ have elevated triglycerides and hepatic steatosis that could be reversed with PEA treatment. Band intensities were measured using ImageJ. Membranes were cut prior to antibody hybridization to allow for the probing of multiple targets on one membrane. Data are presented as mean ± SD. *p < 0.05, **p < 0.01 relative to *Pcyt2*^+*/*+^; ^*#*^*p* < 0.05, ^*##*^*p* < 0.01 relative to *Pcyt2*^+*/−*^.

The most prominent effect of PEA on *Pcyt2*^+*/−*^ liver was the increased phosphorylation/inactivation of Foxo1. pFoxo1 was almost absent (reduced 86%) in *Pcyt2*^+*/−*^ (Fig. 5A) and probably is the most responsible for the increased gluconeogenesis in *Pcyt2*^+*/−*^ liver (Fig. 1). PEA also reduced the elevated (43%) Pkcα by 3.29-fold and did not affect Pkcβ (Fig. 5A). On the other hand, mTorc1 and p-p70S6K increased 2.28- and 5.76-fold in *Pcyt2*^+*/−*^ and an additional 77% and 2.3-fold with PEA. Sirt1 and Ampkα activity were unchanged in fed *Pcyt2*^+*/−*^ and unaltered by PEA. Pgc1α that is reduced 50–57% in *Pcyt2*^+*/−*^ was not altered with PEA supplementation (Fig. 5B). Taken together, supplementation had a prominent effect on amino acid metabolism, by a strong inhibitory effect on gluconeogenesis (Foxo1) and stimulatory effect on protein synthesis (mTorc1). In addition, PEA was able to attenuate hepatic steatosis. *Pcyt2*^+*/−*^ hepatic TAG content was elevated by 76% relative to *Pcyt2*^+*/*+^ littermates and attenuated 26% by PEA supplementation (Fig. 5C) and this is reflected histologically where PEA treated mice show a reduction in lipid droplet accumulation (Fig. 5D).

### PEA attenuates ***Pcyt2***^+***/−***^ liver inflammation

**Figure 6:**
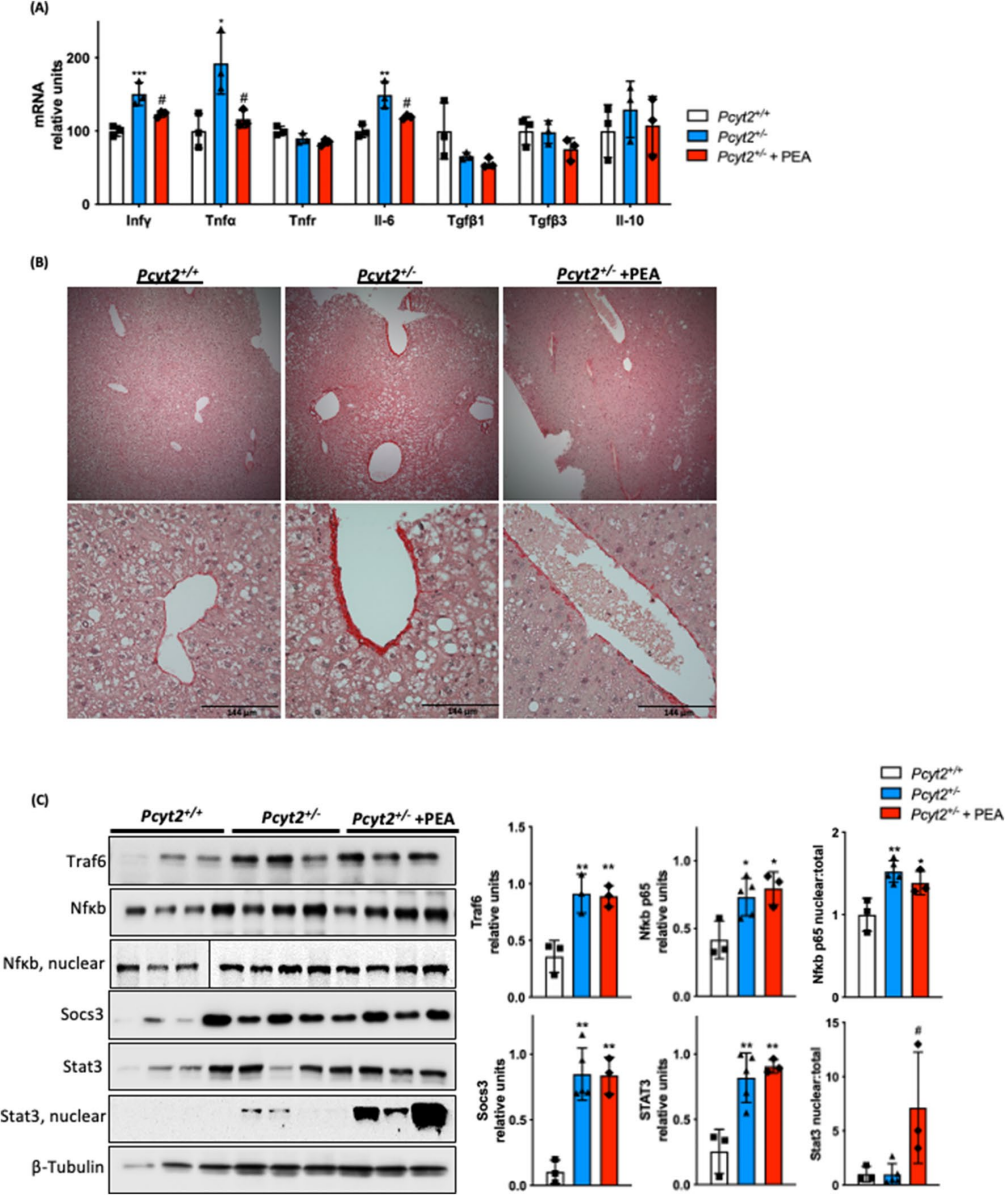

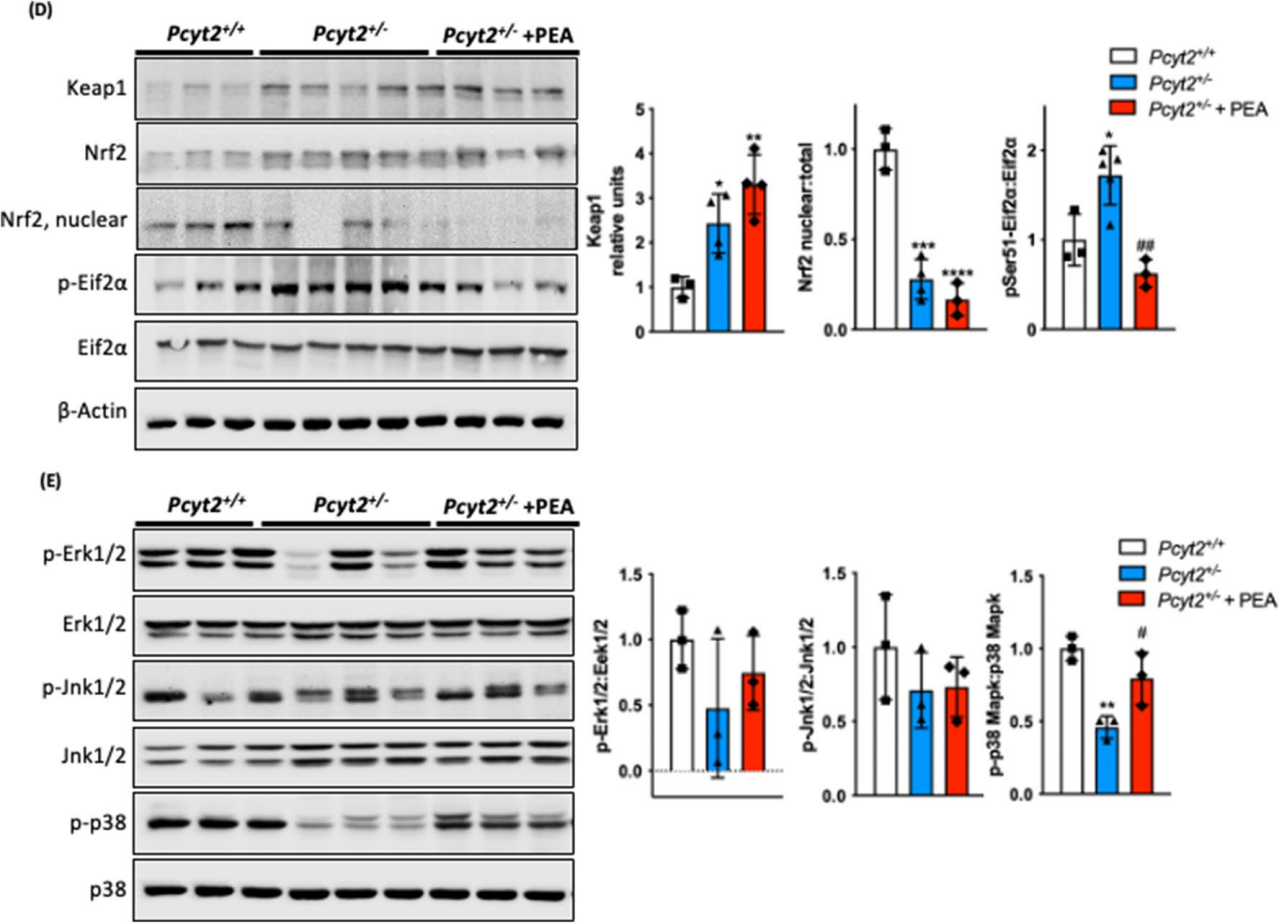
PEA reduces hepatic inflammation. (**A**) mRNA expression of liver pro- and anti-inflammatory genes in 8-mo *Pcyt2*^+*/*+^, *Pcyt2*^+*/−*^ and *Pcyt2*^+*/−*^ + PEA mice treated for 12 weeks (n = 3). GAPDH used as loading control. (**B**) Picrosirius stain of showing that 8-mo *Pcyt2*^+*/−*^ liver have increased collagen deposition that could be reversed with PEA. (**C**) Immunoblot analysis of transcription factors from Jak/Stat and Nfκb pathways: Traf6, Nfκb-p65, Socs3, Stat3; (**D**) Keap1/Nrf2 and pEif2α activation: Keap1, Nrf2, pSer^51^-Eif2α, Eif2α and (**E**) Stress kinases**:** p-Erk1/2, Erk1/2, p-Jnk1/2, Jnk1/2, p-p38 Mapk, p38 Mapk in 8-mo *Pcyt2*^+*/*+^, *Pcyt2*^+*/−*^ and *Pcyt2*^+*/−*^ + PEA mice (n = 3–4). Nfκb-p65 nuclear *Pcyt2*^+*/*+^ bands are from a gel separate from the *Pcyt2*^+*/−*^ and *Pcyt2*^+*/−*^ + PEA bands, but were ran under the same conditions. Band intensities were measured using ImageJ. Membranes were cut prior to antibody hybridization to allow for the probing of multiple targets on one membrane. Data are presented as mean ± SD. *p < 0.05, **p < 0.01, ***p < 0.001 relative to *Pcyt2*^+*/*+^; ^*#*^*p* < 0.05, ^*##*^*p* < 0.01 relative to *Pcyt2*^+*/−*^.

Next, we probed whether PEA improves hepatic inflammation. Expression of proinflammatory cytokines *Infγ*, *Tnfα* and *Il-6* were increased by 50%, 92%, and 49%, respectively, in *Pcyt2*^+*/−*^ liver (Fig. 6A). PEA supplementation reduces the elevated *Infγ*, *Tnfα* and *Il-6* by 18%, 40% and 20%. *Tnfr* showed no significant differences across all groups nor did anti-inflammatory *Tgfβ1*, *Tgfβ3* and *Il-10*. Histological staining with picrosirius red for collagen deposition shows the PEA is able to alleviate the fibrosis seen in *Pcyt2*^+*/−*^ liver (Fig. 6B).

We further investigated the involvement of PEA in JAK/STAT and NFkB pathways (Fig. 6C); Keap1/Nrf2 and pEifα1 activation (Fig. 6D); and stress kinases (Fig. 6E). *Pcyt2*^+*/−*^ mice exhibit a Traf6 increase by 2.55-fold and NFkB increase by 77%. Furthermore, total NFκb was elevated ~ 2-fold indicating its increased activation in *Pcyt2*^+*/−*^ and not modified by PEA. Socs3 protein was elevated by 8.32-fold and Stat3 by 3.22-fold in *Pcyt2*^+*/−*^*.* Nuclear Stat3 was minimally raised in *Pcyt2*^+*/−*^ relative to *Pcyt2*^+*/*+^, and PEA induced a dramatic (7.25-fold) increase in nuclear Stat3 activation. Keap1, the negative regulator of cytoprotective nuclear factor Nrf2, was increased by 2.43-fold and accordingly nuclear Nrf2 was decreased by 72% in *Pcyt2*^+*/−*^ and a further 40% with PEA. Phosphorylation of translation initiation factor Eif2α was increased by 72% in *Pcyt2*^+*/−*^ and decreased by 64% by PEA. Phosphorylation of Erk1/2 and Jnk1/2 were unaffected across all groups however, p-p38 Mapk that was decreased by 54% in *Pcyt2*^+*/−*^ was improved 73% by PEA supplementation. Taken together, these data established that old *Pcyt2*^+*/−*^ liver inflammation was characterized with upregulated Nfkb and pEif2a and reduced Keap1/Nrf2, Stat3, and p-p38 activity that all except Nfkb were regressed with 2 months of PEA supplementation at physiologically relevant levels.

**Figure 7:**
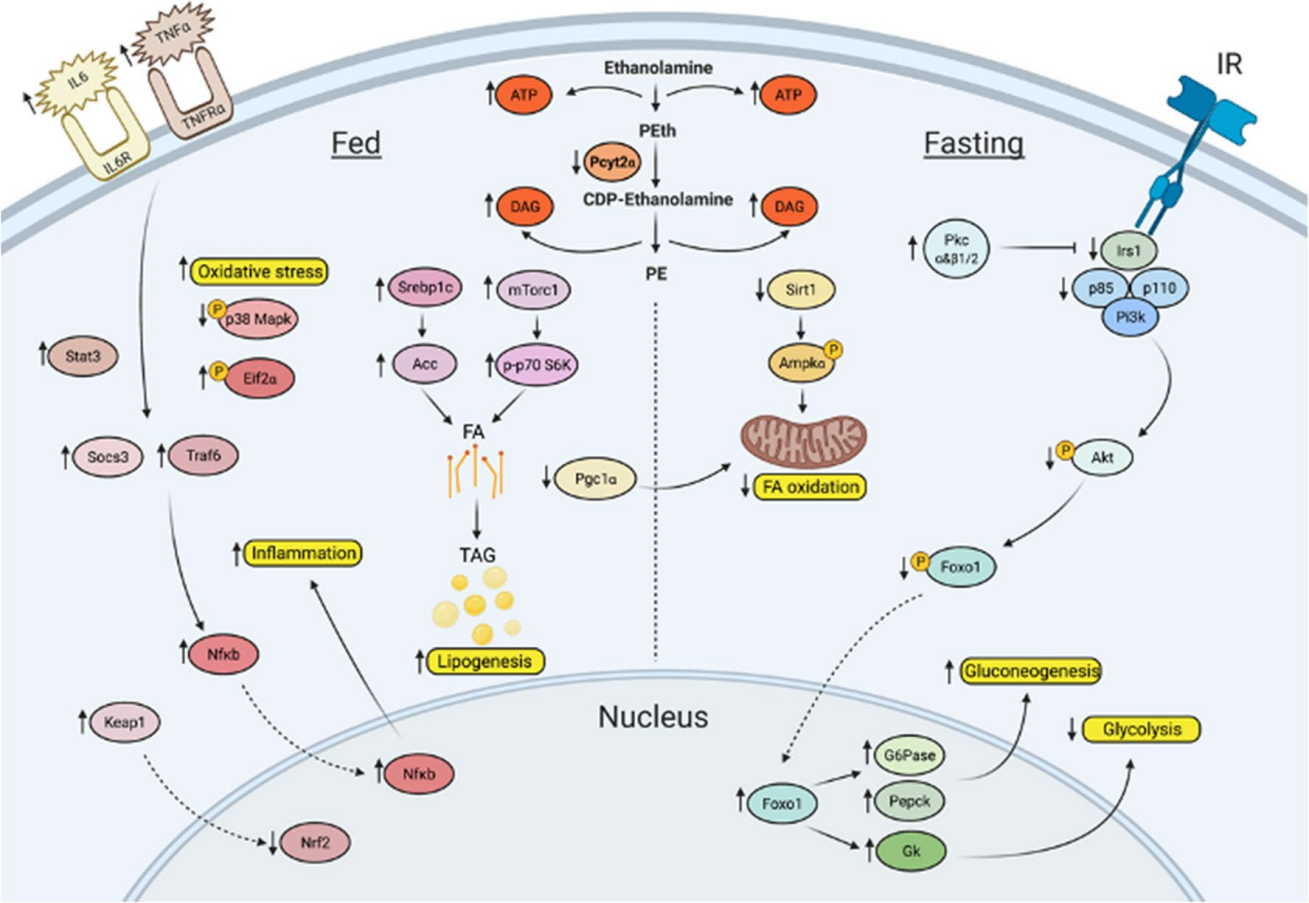
Working model for age-dependent development of *Pcyt2*^+*/−*^ hepatic steatosis and inflammation, and reversion with PEA. Image was created with BioRender.com.

The working model for the signaling perturbations in *Pcyt2*^+*/−*^ NASH is illustrated in Fig. 7. It shows how reduced de novo synthesis of the membrane PE phospholipid results in metabolic and genetic adaptations to maintain membrane bilayers and accommodate unused metabolic intermediates, resulting in changes in glucose and FA metabolism and inflammation that contribute to NASH development.

## Discussion

Various mouse models of NASH have been reported, however, few recapitulate both the metabolic and histopathological features and often require special diets. For example, the most widely used diet to induce NASH is a choline/methionine deficient diet. However, this diet is criticized because it causes weight loss and does not induce insulin resistance, an important risk factor for NASH^24^. Here, we show that *Pcyt2*^+*/−*^ mice are an ideal translation model for the human disease because they develop NASH over time and within the context of key risk factors for the human condition (obesity and metabolic syndrome). In this study we focus on the mechanisms of age-related development of NASH in *Pcyt2*^+*/−*^ mice.

At young age (2-mo) *Pcyt2*^+*/−*^ have no clinical symptoms of NAFLD, However, 2-mo *Pcyt2*^+*/−*^ exhibit early defects in fatty acid metabolism that favour FA synthesis and persist into adulthood, Adult (6-8mo) *Pcyt2*^+*/−*^ exhibit a fasting-specific deficit in Pi3k/Akt signalling with a shift to increased glucose production by gluconeogenesis, and reduced lipolysis and FA oxidation ^20^. Together these impairments cause an increase in liver glycogen and lipid content, leading to steatosis and metabolic syndrome. In humans, NASH is diagnosed only by the hepatic histological findings. Adult *Pcyt2*^**+/−**^ exhibit all of the criteria for biopsy proven NASH ^42^: steatosis, hepatocyte ballooning degeneration with Mallory bodies, inflammatory infiltration of macrophages, and fibrosis. Hepatic inflammation is the critical factor distinguishing NASH from simple steatosis and is further demonstrated in adult *Pcyt2*^**+/−**^ in the enrichment of proinflammatory pathways and increased mRNA/protein expression of proinflammatory modulators. Adult *Pcyt2*^**+/−**^ exhibit elevated serum ALP, AST and ALT and decreased albumin, which is suggestive of hepatocellular damage and progressive liver functional impairment^43,44^. Therefore, this firmly establishes the adult *Pcyt2*^**+/−**^ liver pathology as NASH.

A major paradox of type 2 diabetes is the selective impairment in the insulin mediated liver processes. The adult *Pcyt2*^+*/−*^ model recapitulates this by exemplifying the insulin resistant condition at older age with concomitantly increased gluconeogenesis and lipogenesis. In fasting, the contribution of glucose to the liver energy production is low, as shown by reduced glucose uptake and expression/activity of the glycolytic genes in older *Pcyt2*^+*/−*^. Previous metabolic profiling demonstrated increased plasma glucose, reduced plasma glycerol (reduced lipolysis) and increased plasma acyl carnitines (reduced FA oxidation), and an altered amino acid metabolism in older *Pcyt2*^+*/−*^, with the notable increase in major anaplerotic precursor, glutamine^20,45^. A suppression of glucose and FA utilization limits the available pathways for energy production and thus, forces anaplerosis to replenish TCA cycle intermediates and permit its continued function. Subsequent obligate cataplerosis is linked to glucose and lipid synthesis in the liver^46^ and thus, an active contribution of glutamine/amino acids to TCA reconciles the contradiction of simultaneously increased hepatic glucose and FA production in *Pcyt2*^+*/−*^. This mechanism is corroborated in mice^47^ and human subjects with NAFLD^48^, showing the connection between increased anaplerosis and intrahepatic TAG accumulation, gluconeogenesis and insulin resistance.

We established that the mitochondria regulators Sirt1/p-Ampkα are inhibited and the main regulator of FA synthesis Srebp1c is upregulated early in asymptomatic young *Pcyt2*^+*/−*^, as direct consequence of *Pcyt2* gene deletion, not because of liver steatosis or insulin resistance, which develops at adult age. *Pcyt2* heterozygosity leads to reduced flux by the Kennedy pathway and an accumulation of unused intermediates (DAG, ATP) that need to be accommodated^21^. An early increase in Srebp1 and FA synthesis is necessary to form TAG from DAG to reduce DAG levels and thus, even at young age, glucose and FA usage for energy are suppressed^21^. The function of conventional, DAG dependant PKCα/β1/2 is tightly linked to phospholipid homeostasis and stimulates ET/Pcyt2 activity^3,8^ and not surprisingly they were also constitutively upregulated in young *Pcyt2*^+*/−*^. PKCα and PKC β1/2 among many other functions, are well-known inhibitors of insulin signaling^49^. As DAG/TAG accumulate, constitutively increased PKC, which occurs prior to NASH development, could progressively diminish insulin signaling in older *Pcyt2*^+*/−*^. These findings are in line with previously established reduced FA oxidation and increased DAG in asymptomatic young *Pcyt2*^+*/−*^^21,50^, indicating that these are the main drivers of impaired insulin signaling and adult-onset NASH developemnt even on a regular chow diet. Together the inherent transcriptional and metabolic adaptations to reduced Pcyt2 expression and activity cause a progressive metabolic dysfunction, culminating in obesity, insulin resistance, and hypertriglyceridemia^21,37^.

*Pcyt2*^+*/−*^ liver shows heavily diminished Irs1, p-Irs1, PI3K and Akt1/2 and pSer^473^-Akt activation by Torc2 specifically in the fasted state, suggesting an increased protein degradation which agree with higher anaplerotic demands for amino acids, described above. There is also an increased gluconeogenesis in fasted state, showing a failed blockade of glucose production by insulin and increased *Pepck* and *G6Pase* expression and activity, controlled by gluconeogenic transcription factor Foxo1^51^. Indeed, *Pcyt2*^+*/−*^ phosphorylation of Foxo1 is decreased, leading to increased transcriptional activity which is in congruance with observed increase in gluconeogenesis.

Supplementation of the artificial substrate PEA is able to ameliorate *Pcyt2*^+*/−*^ NASH. PEA supplementation increases total mTorc1 and mTorc1 substrate p70S6K levels, indicting a stimulation of protein synthesis, which may help rectify the abnormal amino acid metabolism shown in 6-mo *Pcyt2*^+*/−*^ microarray. PEA increases the activation of Stat3 and restores the diminished phosphorylation of Foxo1, allowing for the improvement in NASH^52,53^. Notably, DAG-regulated Pkcα is reduced by PEA supplementation, suggesting an attenuation of DAG accumulation and improvement in lipid metabolism. PEA supplementation marginally increased *Atgl* and *Pparα* and parallels the effects of over expression of ATGL, an important lipase that governs hepatic TAG turnover and PPARα. Overexpression of ATGL increases FA oxidation, preventing hepatic lipid accumulation and increases PPARα activity, whereas knockdown causes steatosis^54,55^. Thus, it is conceivable that PEA is able to improve *Pcyt2*^+*/−*^ steatosis through stimulating metabolic flux by the Kennedy pathway, restoring membrane phospholipid homeostasis and increasing the utilization of DAG to reestablish energy balance and improve lipid and glucose metabolism.

The beneficial effect of PEA was also evident on several characteristics of the inflammatory response associated with NASH. *Pcyt2*^+*/−*^ livers exhibit an elevated content inflammatory factors *Il-6, Tnfα,* Socs3, Traf6*,* and Nfκb, demonstrating the chronic inflammation characteristic of NASH. PEA significantly attenuated several proinflammatory cytokines and hepatic fibrosis but was not able to reduce the nuclear content of Nfκb. Traf6 activates Nfκb through PI3k signaling^56^ and *Pcyt2*^+*/−*^ Pi3k was not modified by PEA, leading to the consistent activation of NFκb^57^. However, PEA appears to alleviate oxidative stress as p38 Mapk undergoes dephosphorylation in *Pcyt2*^+*/−*^ that is partially restored with PEA supplementation. While p38 activity is typically associated with inflammation, dephosphorylation of p38 Mapk occurs in response to increased Pi3k activity and oxidative stress and is shown to be regulated independently from Erk/Jnk^58,59^. Moreover, p-Eif2α was reduced by PEA, indicating an improvement of *Pcyt2*^+*/−*^ oxidative stress. In mice with NAFLD induced with high fructose diet, phosphorylation of Eif2α is increased to protect hepatocytes from oxidative stress, fibrosis and death^60^. Nuclear content of Stat3 is dramatically increased with PEA. Stat3 plays a complex role in liver inflammation, having both pro- and anti-inflammatory functions, but importantly STAT3 has been shown protect against hepatocellular damage and attenuate the inflammatory response in models of liver injury^61,62^.

An important mechanism in fibrogenesis is the generation of mesenchymal cells through epithelial-to-mesenchymal transition, which deposit extracellular matrix once activated^63^. PEA most likely attenuated this process through stimulation of the Kennedy pathway, known to be a regulator of the reverse process, i.e., the mesenchymal-to-epithelial transition^64^ and therefore inhibition of extracellular matrix deposition. The reduction in fibrosis is in line with an improvement in hepatic reduction of *Tnfα* and *Il-6*^65^.

In summary, this work emphasizes the importance of membrane phospholipid homeostasis in metabolic disease development and progression. Our work established for the first time that a negative metabolic energy balance, due to reduced membrane lipid synthesis, that results in excessive production of FA to accommodate unused intermediates could lead to all known features of NASH, including steatosis and inflammation. PEA supplementation was able to reverse *Pcyt2*^+*/−*^ hepatic steatosis and inflammation. These effects indicate the CDP-Etn Kennedy pathway as a target in fatty liver disease and the therapeutic potential of PEA. Given the increasing global presence of obesity and type 2 diabetes and lack of drug therapy for NASH, identifying new treatment options is critical.

## Data and resource availability

The datasets generated during the current study are available from the corresponding author upon request. The resources and suppliers used in this study have been provided above.

## Supplementary Information

Supplementary Information 1.

Supplementary Information 2.
