# Author Correction: Pcyt2 deficiency causes age-dependent development of nonalcoholic steatohepatitis and insulin resistance that could be attenuated with phosphoethanolamine

**DOI:** 10.1038/s41598-023-36725-w

**Published:** 2023-06-22

**Authors:** Sophie Grapentine, Rathnesh K. Singh, Poulami Basu, Sugashan Sivanesan, Gabriela Mattos, Oreoluwa Oresajo, Jasmine Cheema, Wendwesen Demeke, Vernon W. Dolinsky, Marica Bakovic

**Affiliations:** 1 Department of Human Health and Nutritional Sciences, University of Guelph, 50 Stone Rd E, Guelph, N1G2W1 Canada; 2 Department of Pharmacology and Therapeutics, University of Manitoba, Winnipeg, Canada

Correction to: *Scientific Reports* 10.1038/s41598-022-05140-y, published online 20 January 2022

The original version of this Article contained errors.

There is a repeated error throughout the Article where the compound used in the study Phosphonoethylamine (PEA) was incorrectly given as Phosphoethanolamine (PEtn).

Figures 1, 2, 3, 4, 5, 6 and 7 also contained this error where the abbreviation PEA was incorrectly given as PEtn. The original Figures 1, 2, 3, 4, 5, 6 and 7 and the accompanying legends appear below.

**Figure 1:**
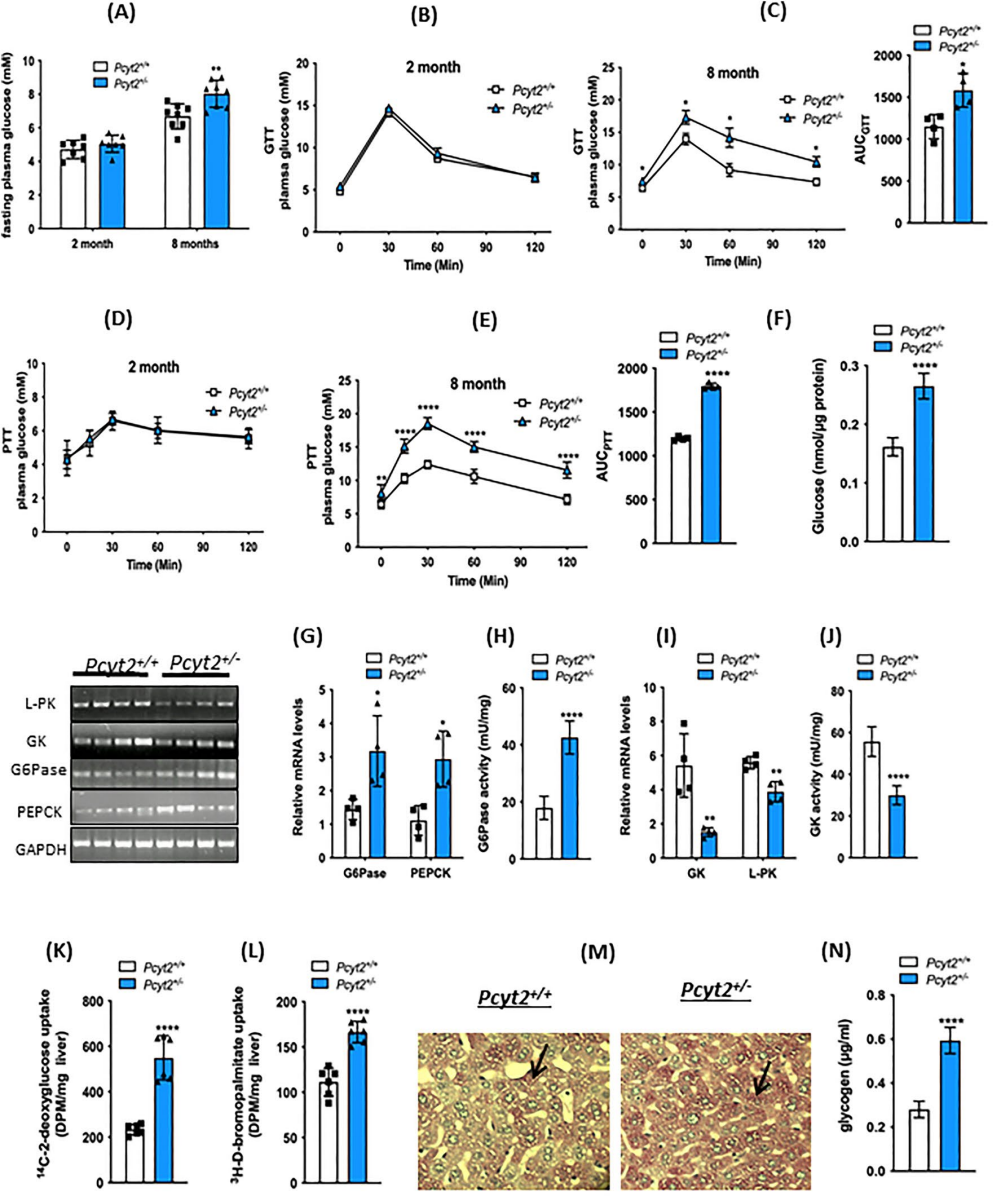
Glucose metabolism is altered in older *Pcyt2*^+*/−*^ mice. (**A**) Fasting glucose levels for 2-mo and 8-mo *Pcyt2*^+*/*+^ and *Pcyt2*^+*/−*^ mice. (**B**) Intraperitoneal glucose tolerance test for 2-mo and (**C**) 8-mo *Pcyt2*^+*/*+^ and *Pcyt2*^+*/−*^ and area under the curve (n = 4). (**D**) Intraperitoneal pyruvate tolerance test for 2-mo and (**E**) 8-mo *Pcyt2*^+*/*+^ and *Pcyt2*^+*/−*^ and area under the curve (n = 4). (**F**) Glucose production in primary hepatocytes (n = 12). (**G**) Relative mRNA expression levels of gluconeogenic enzymes *G6Pase* and *Pepck* (n = 4) and (**H**) G6Pase enzymatic activity (n = 12) in 8-mo *Pcyt2*^+*/*+^and *Pcyt2*^+*/−*^ mice. (**I**) Relative mRNA expression levels of glycolytic enzymes *Gk* and *L-Pk* (n = 4) and (**J**) GK enzymatic activity (n = 12) in 8-mo *Pcyt2*^+*/*+^ and *Pcyt2*^+*/−*^ mice. (**K**) [^14^C]deoxyglucose and (**L**) [^3^H]bromopalmitate uptake in liver (n = 6). (**M**) Periodic acid-Schiff staining of liver sections for glycogen in 8-mo *Pcyt2*^+*/*+^ and *Pcyt2*^+*/−*^ mice. (**N**) Liver glycogen content of 8-mo *Pcyt2*^+*/*+^ and *Pcyt2*^+*/−*^ mice (n = 12). Band intensities were measured using ImageJ. Data are presented as mean ± SD. *p < 0.05; **p < 0.01; ***p < 0.001; ****p < 0.0001.

**Figure 2:**
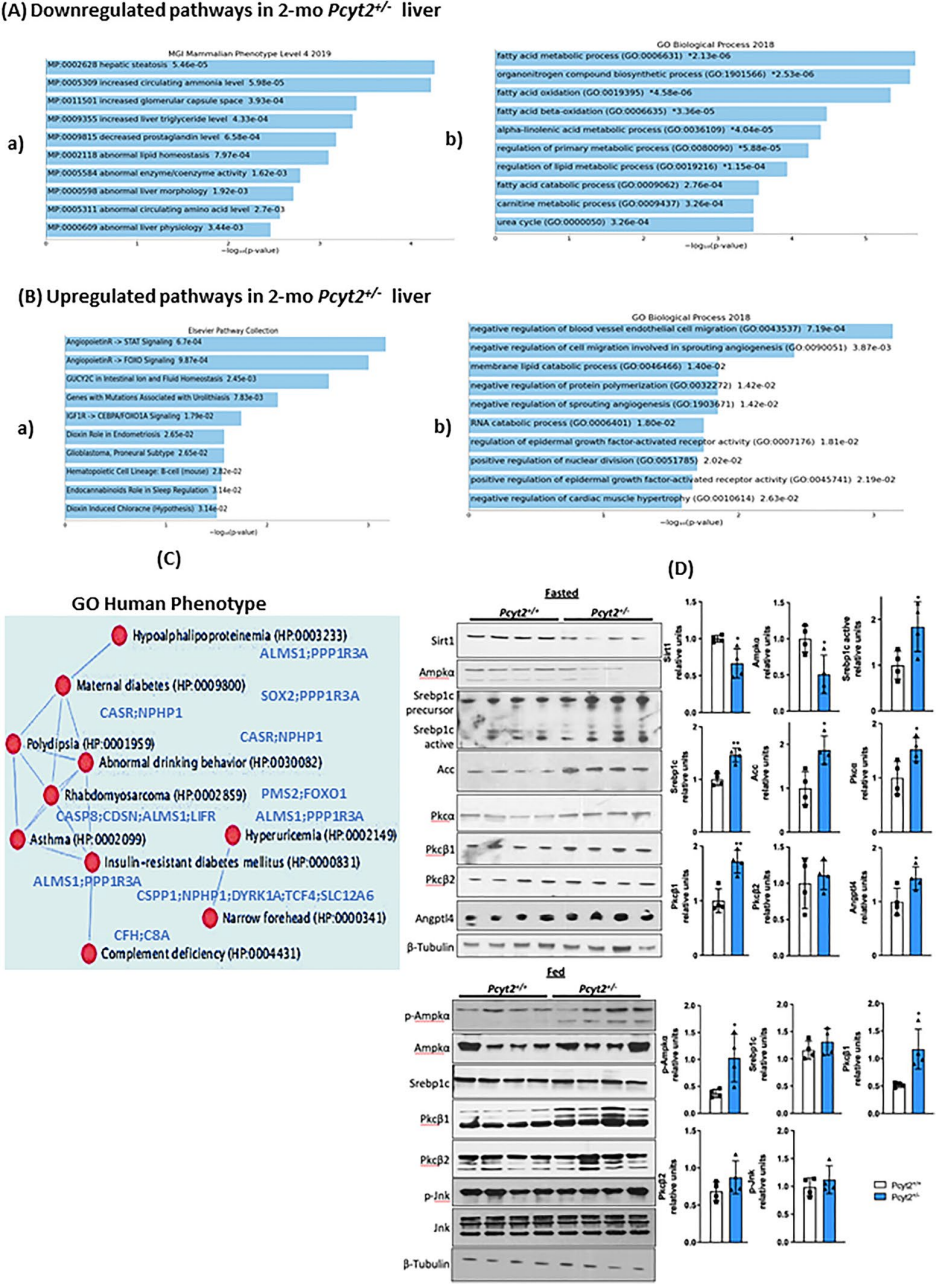
Analysis of down- and upregulated pathways in 2-mo *Pcyt2*^+*/−*^ liver. (**A**) Downregulated genes from 2-mo *Pcyt2*^+*/−*^ liver were analyzed with Enrichr (https://maayanlab.cloud/Enrichr/)^30,31,32^ (n = 3). (**A-a**) Pathway analysis (MGI Mammalian Phenotypes Level 4–2019) established the most enriched terms include hepatic steatosis and increased circulating ammonia. (**A-b**) The Gene Onthology (GO) analysis (GO Biological Processes 2018) further shows that the most significantly downregulated were processes of fatty acid oxidation and nitrogen degradation. The most frequently downregulated GO genes from phospholipids and fatty acid metabolism and nitrogen (urea cycle, arginine) are indicated in the clustergram. (**B**) Upregulated genes from 2-mo *Pcyt2*^+*/−*^ liver were analyzed with Enrich (n = 3). (**B-a**) The cluster analysis for the Elsevier Pathway Collection identified as elevated the Igf2- and Ang4/Ang2-Foxo1 pathways and ion/amino acid transport. (**B-b**) GO Biological Processes 2018 of the most upregulated genes identified as the most important the processes linked to Ang2/Ang4 functions in regulation of angiogenesis and Egfr signaling. (**C**) Go:Human Phenotype establish a gene/disease network for young *Pcyt2*^+*/−*^ with significant risk for development of maternal- and type 2- diabetes and related pathologies. (**D**) Immunoblot analysis of lipid pathways in fasted and fed *Pcyt2*^+*/*+^ and *Pcyt2*^+*/−*^ (n = 4). Band intensities were measured using ImageJ. Data are presented as mean ± SD. *p < 0.05; **p < 0.01.

**Figure 3:**
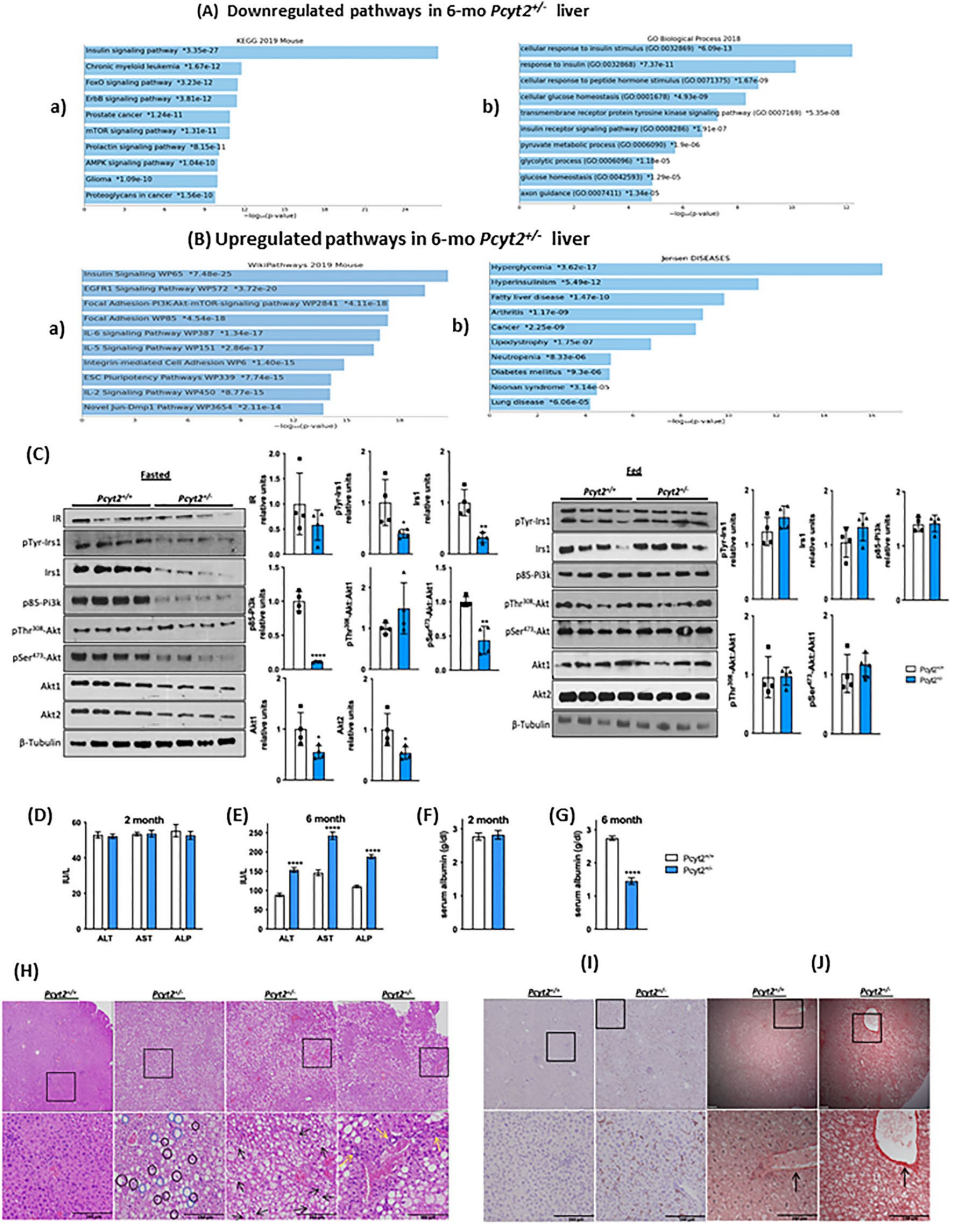
Analysis of down- and upregulated pathways in 6-mo *Pcyt2*^+*/—*^liver*.* (**A**) Downregulated genes of 6-mo *Pcyt2*^+*/−*^ liver were analyzed with Enrichr (https://maayanlab.cloud/Enrichr/)^30,31,32^ (n = 3). (**A-a**) The Mouse 2019 KEGG^60,61,62^ pathway analysis and genes cluster analysis indicated that the most downregulated pathways included the growth factor (insulin, ErB, prolactin, cancer) pathways, and mTOR and AMPK pathways. (**A-b**) GO Biological Processes 2018 showed that the most downregulated processes were insulin signaling and glucose metabolism. (**B**) Upregulated genes of 6-mo old *Pcyt2*^+*/−*^ were analyzed with Enrich (n = 3). (**B-a**) WikiPathway Mouse 2019 established the most significantly upregulated gene clusters and pathways for insulin/EGFR signaling and pro inflammatory pathways mediated by IL2, IL9, IL5, IL6, and IL7 (**B-b**) Jansen Diseases analysis showed that the most upregulated were gene involved in hyperglycemia, hyperinsulinemia, and fatty liver disease. (**C**) Immunoblot analysis of Pi3k/Akt pathway in fasted and fed 6-mo *Pcyt2*^+*/−*^ (n = 4). Serum levels of liver enzymes ALT, AST, ALP in (**D**) 2-mo and (**E**) 6-mo *Pcyt2*^+*/−*^ (n = 12). Serum levels of albumin in (**F**) 2-mo and (**E**) 6-mo *Pcyt2*^+*/−*^ (n = 12). Histology of 8-mo liver with (**H**) H&E stain showing steatosis (blue circles), ballooned hepatocytes (black circles) with Mallory-Denk bodies (black arrows) and lobular inflammation (yellow arrows); (**I**) immunohistochemical stain with F4/80 showing macrophage infiltration; (**J**) Picrosirius red stain showing collagen deposition. Band intensities were measured using ImageJ. Data are presented as mean ± SD. *p < 0.05, **p < 0.01, ***p < 0.001, ****p < 0.0001.

**Figure 4:**
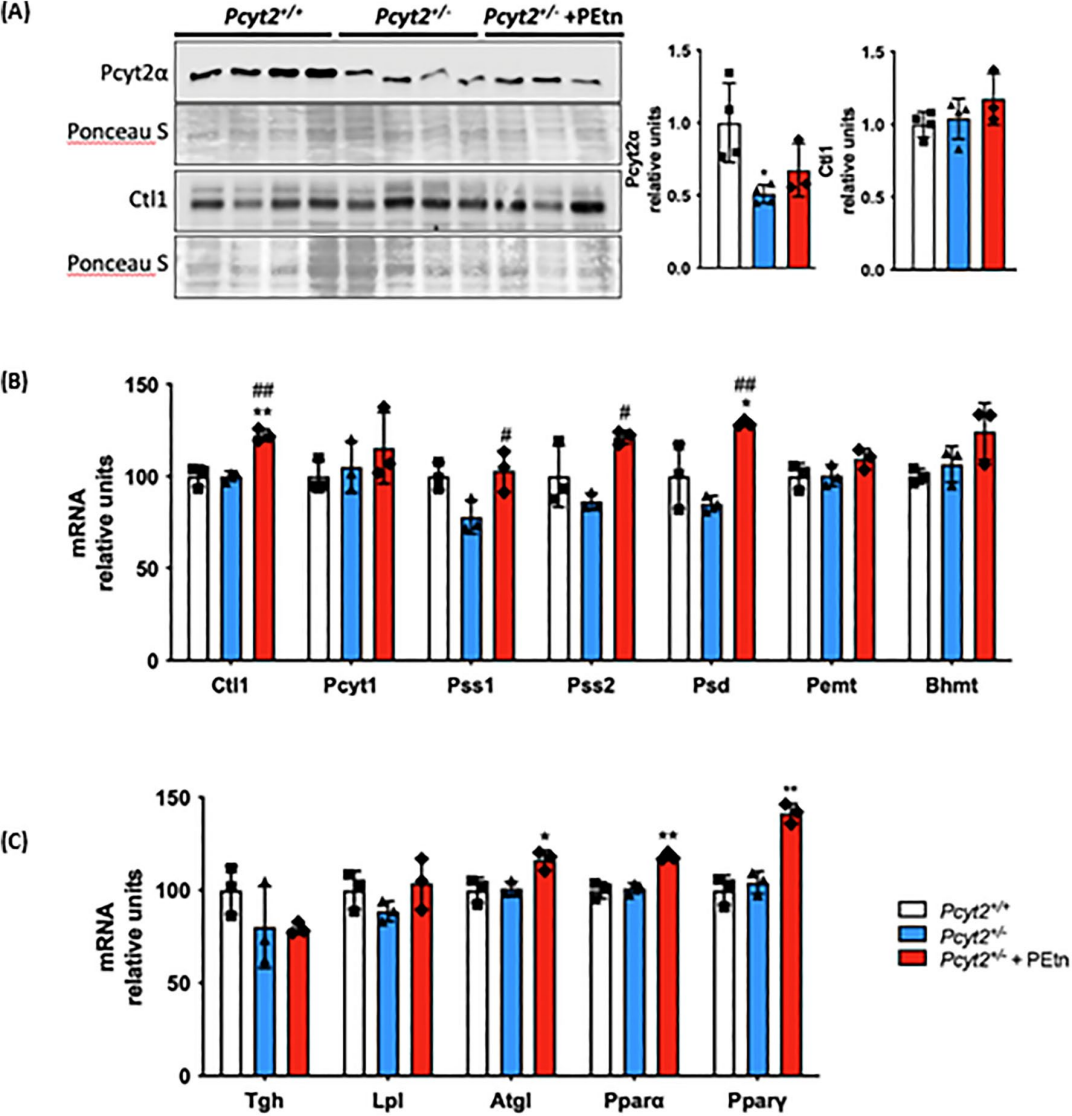
PEtn stimulates phospholipid and triglyceride metabolic genes. (**A**) Immunoblot analysis of liver Pcyt2α and Ctl1 in 8-mo *Pcyt2*^+*/*+^, *Pcyt2*^+*/−*^ and *Pcyt2*^+*/−*^  + PEtn mice treated for 12 weeks (n = 3–4). mRNA analysis of (**B**) phospholipid and (**C**) lipid metabolism genes in *Pcyt2*^+*/*+^, *Pcyt2*^+*/−*^ and *Pcyt2*^+*/−*^  + PEtn mice. GAPDH used as loading control. Band intensities were measured using ImageJ. Membranes were cut prior to antibody hybridization to allow for the probing of multiple targets on one membrane. Data are presented as mean ± SD. *p < 0.05, **p < 0.01 relative to *Pcyt2*^+*/*+^; ^*#*^*p* < 0.05, ^##^*p* < 0.01 relative to *Pcyt2*^+*/−*^.

**Figure 5:**
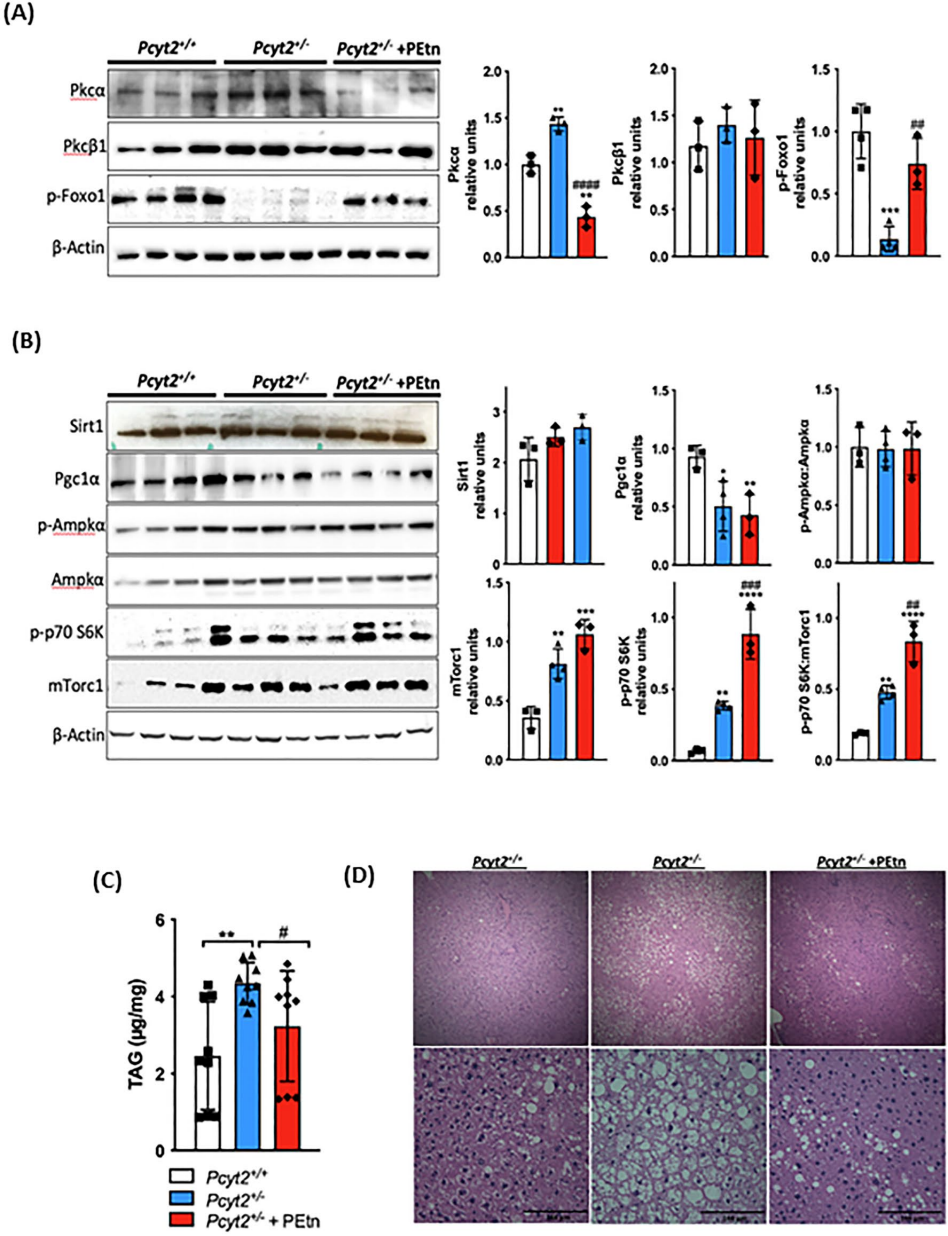
PEtn inactivates Foxo1 and activates mTorc1 signaling pathway and reduced hepatic steatosis. Immunoblot analysis of liver (**A**) Pkcα, Pkcβ1, p-Foxo and (**B**) Sirt1, Pgc1α, p-Ampkα, Ampkα, p70S6K, and mTorc1 in 8-mo fed *Pcyt2*^+*/*+^, *Pcyt2*^+*/−*^ and *Pcyt2*^+*/−*^  + PEtn mice (n = 3–5). (**C**) Liver triglyceride analysis (n = 9) and (**D**) H&E staining showing that 8-mo *Pcyt2*^+*/−*^ have elevated triglycerides and hepatic steatosis that could be reversed with PEtn treatment. Band intensities were measured using ImageJ. Membranes were cut prior to antibody hybridization to allow for the probing of multiple targets on one membrane. Data are presented as mean ± SD. *p < 0.05, **p < 0.01 relative to *Pcyt2*^+*/*+^; ^*#*^*p* < 0.05, ^*##*^*p* < 0.01 relative to *Pcyt2*^+*/−*^.

**Figure 6:**
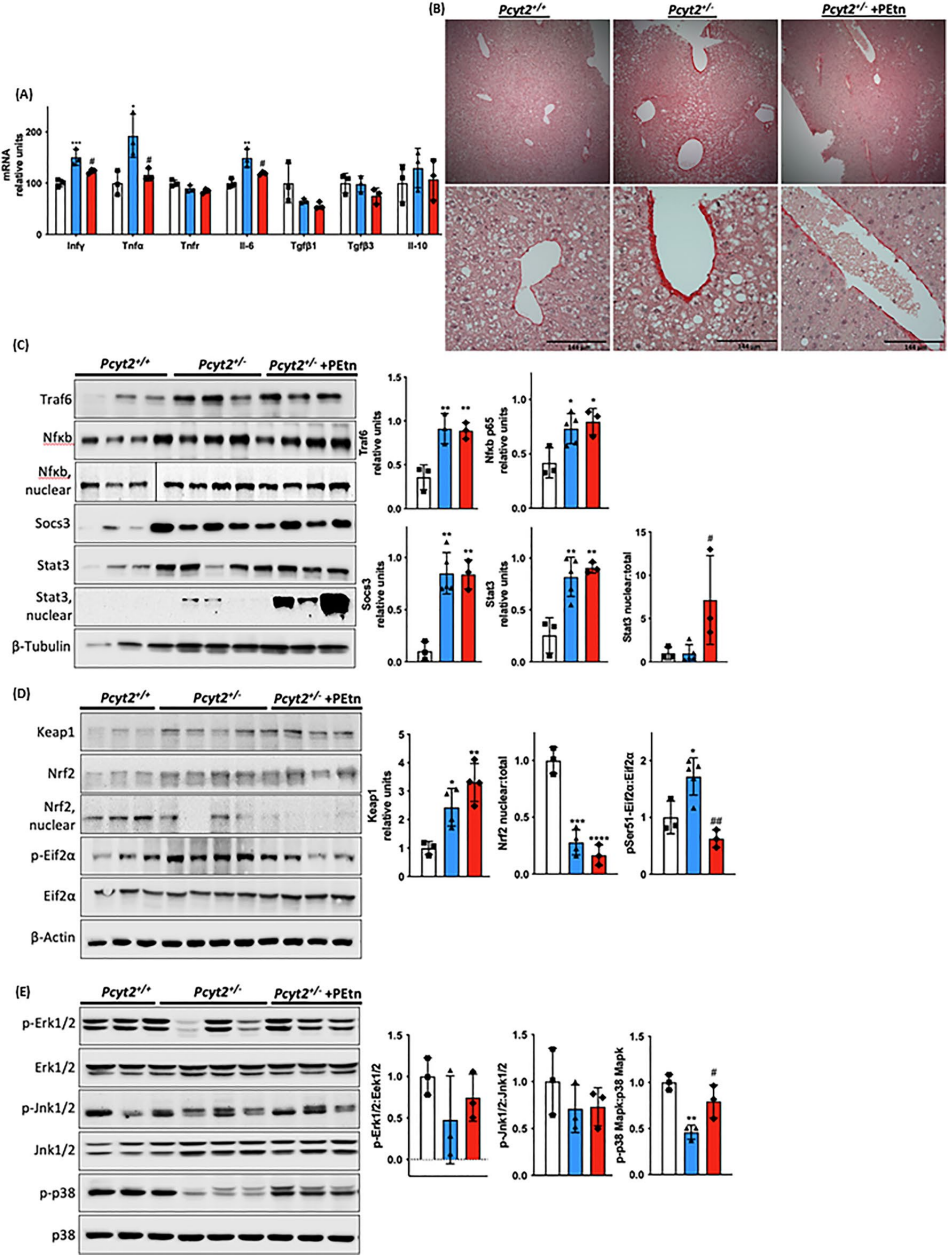
PEtn reduces hepatic inflammation. (**A**) mRNA expression of liver pro- and anti-inflammatory genes in 8-mo *Pcyt2*^+*/*+^, *Pcyt2*^+*/−*^ and *Pcyt2*^+*/−*^  + PEtn mice treated for 12 weeks (n = 3). GAPDH used as loading control. (**B**) Picrosirius stain of showing that 8-mo *Pcyt2*^+*/−*^ liver have increased collagen deposition that could be reversed with PEtn. (**C**) Immunoblot analysis of transcription factors from Jak/Stat and Nfκb pathways: Traf6, Nfκb-p65, Socs3, Stat3; (**D**) Keap1/Nrf2 and pEif2α activation: Keap1, Nrf2, pSer^51^-Eif2α, Eif2α and (**E**) Stress kinases**:** p-Erk1/2, Erk1/2, p-Jnk1/2, Jnk1/2, p-p38 Mapk, p38 Mapk in 8-mo *Pcyt2*^+*/*+^, *Pcyt2*^+*/−*^ and *Pcyt2*^+*/−*^  + PEtn mice (n = 3–4). Nfκb-p65 nuclear *Pcyt2*^+*/*+^ bands are from a gel separate from the *Pcyt2*^+*/−*^ and *Pcyt2*^+*/−*^  + PEtn bands, but were ran under the same conditions. Band intensities were measured using ImageJ. Membranes were cut prior to antibody hybridization to allow for the probing of multiple targets on one membrane. Data are presented as mean ± SD. *p < 0.05, **p < 0.01, ***p < 0.001 relative to *Pcyt2*^+*/*+^; ^*#*^*p* < 0.05, ^*##*^*p* < 0.01 relative to *Pcyt2*^+*/−*^.

**Figure 7:**
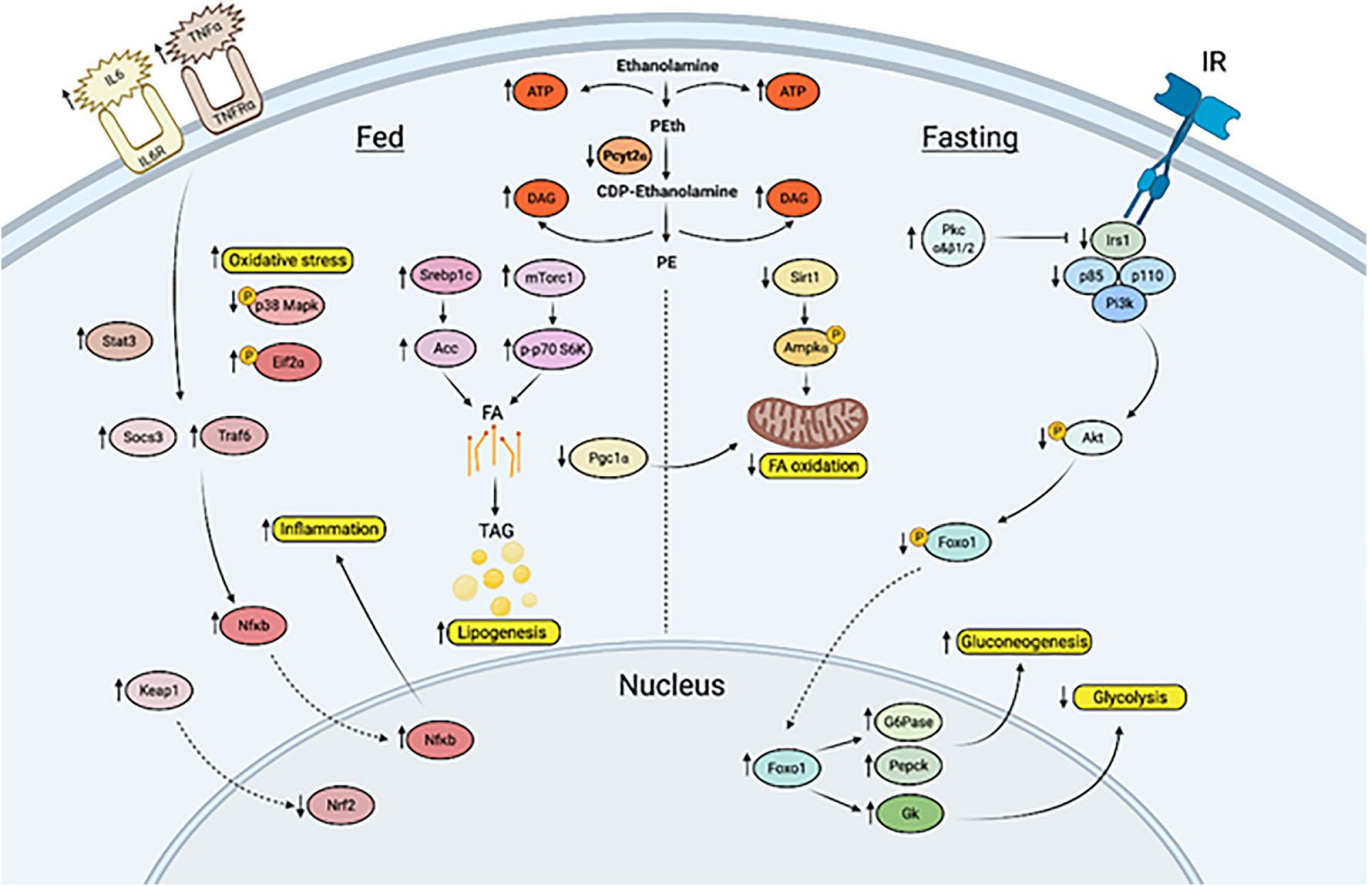
Working model for age-dependent development of *Pcyt2*^+*/−*^ hepatic steatosis and inflammation, and reversion with PEtn. Image was created with BioRender.com.

In addition, References 25, 26 and 27 were omitted and are listed below:

25. Curley-Joseph, J. & Henderson, T. O. 2-Aminoethylphosphonic acid metabolism in the rat. *Lipids* **12**, 75–84 (1977).

26. Alhadeff, J. A. & Doyle Daves, G. 2-Aminoethylphosphonic acid: Distribution in human tissues. *Biochimica et Biophysica Acta (BBA)—General Subjects* **244**, 211–213 (1971).

27. Kafarski, P. Phosphonates: Their Natural Occurrence and Physiological Role. in *Contemporary Topics about Phosphorus in Biology and Materials* (IntechOpen, 2019). doi: 10.5772/intechopen.87155.

The original Article has been corrected.

