# Supplementary material for: Pcyt2 deficiency causes age-dependant development of nonalcoholic steatohepatitis and insulin resistance that could be attenuated with phosphonoethylamine
Source: Sci Rep. 2022 Jan 20;12:1048. doi: 10.1038/s41598-022-05140-y (PMC8776951; doi:10.1038/s41598-022-05140-y)

Figure 1G and H

ORIGINAL | USED IN MANUSCRIPT

Pcyt2$^{+/+}$ *Pcyt2*$^{+/-}$

L-PK

GK

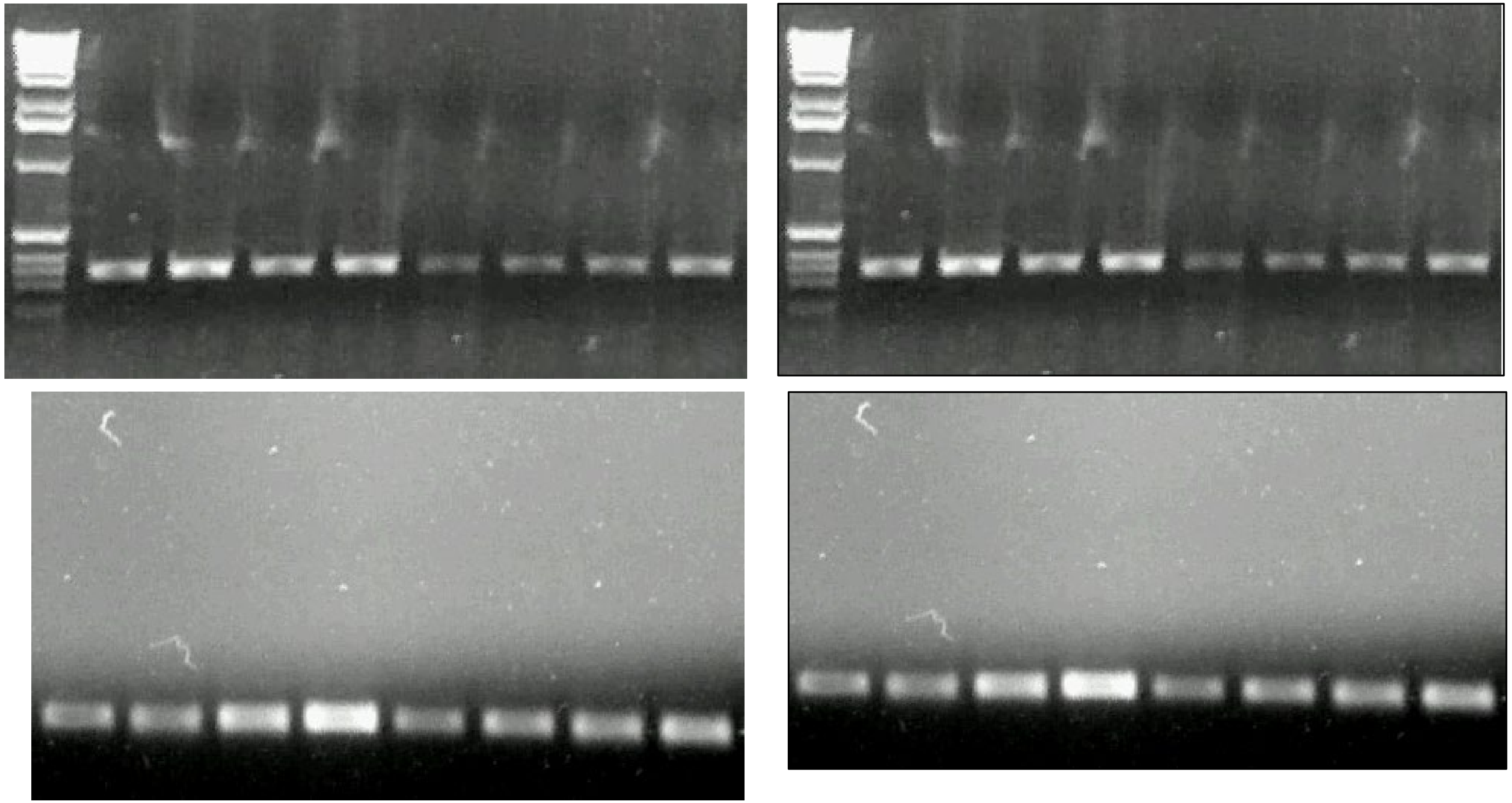

Figure 1G and H

ORIGINAL

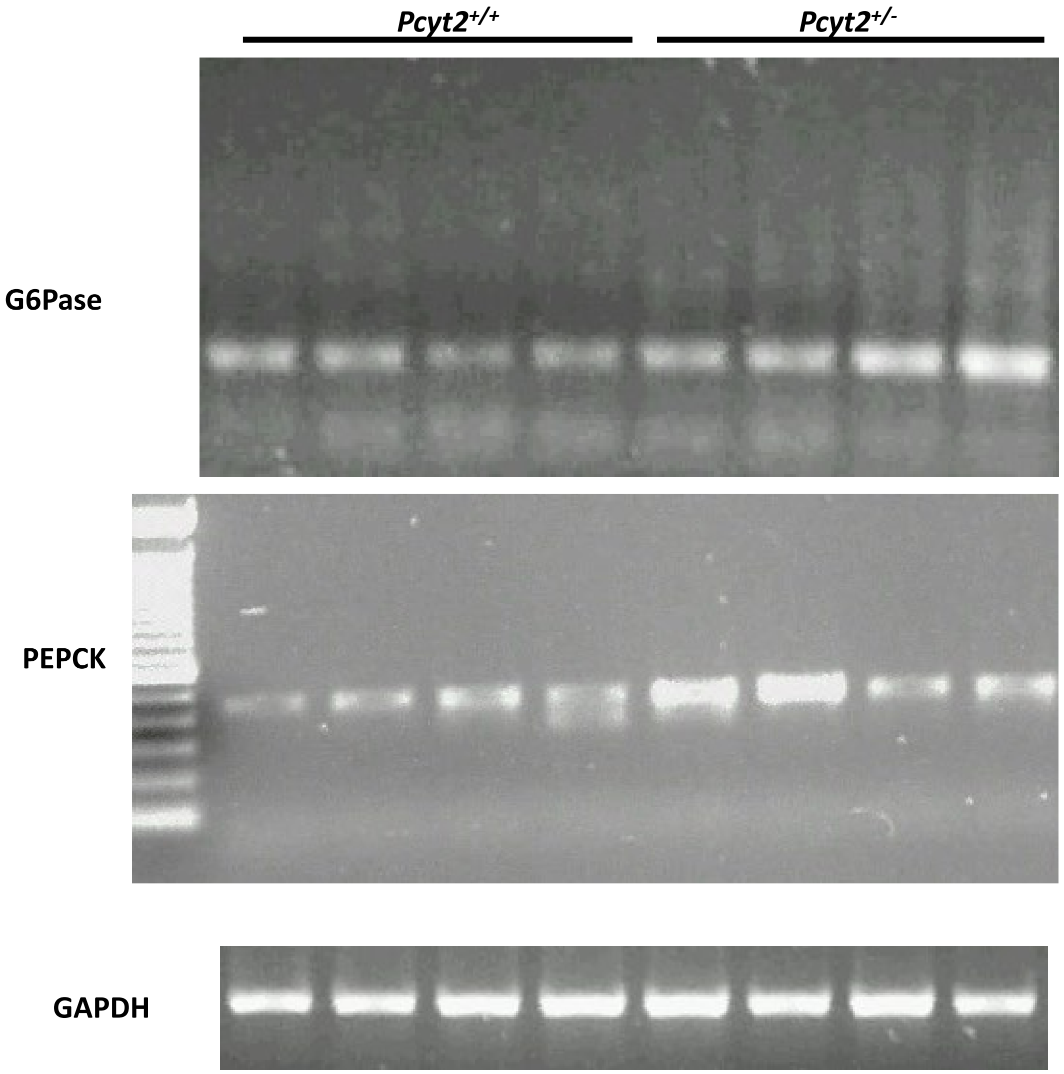

USED IN MANUSCRIPT

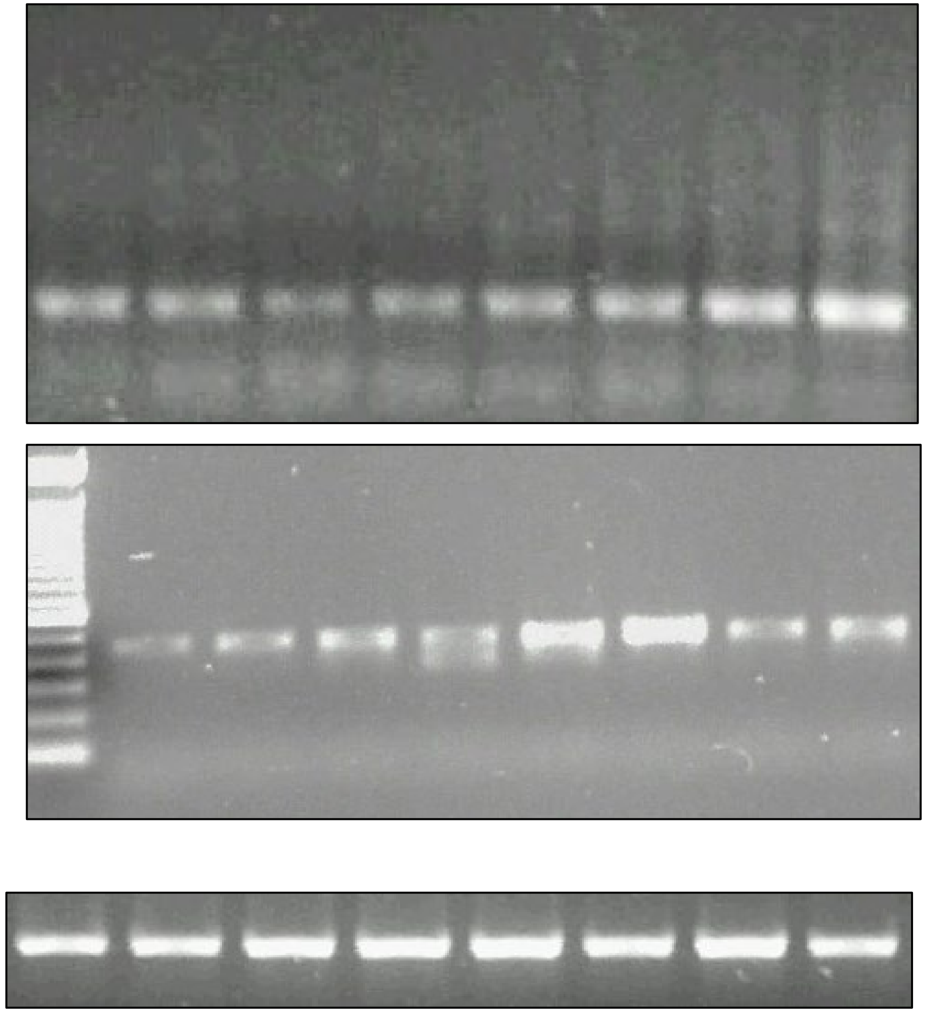

Figure 2D

ORIGINAL

Fasted

USED IN MANUSCRIPT

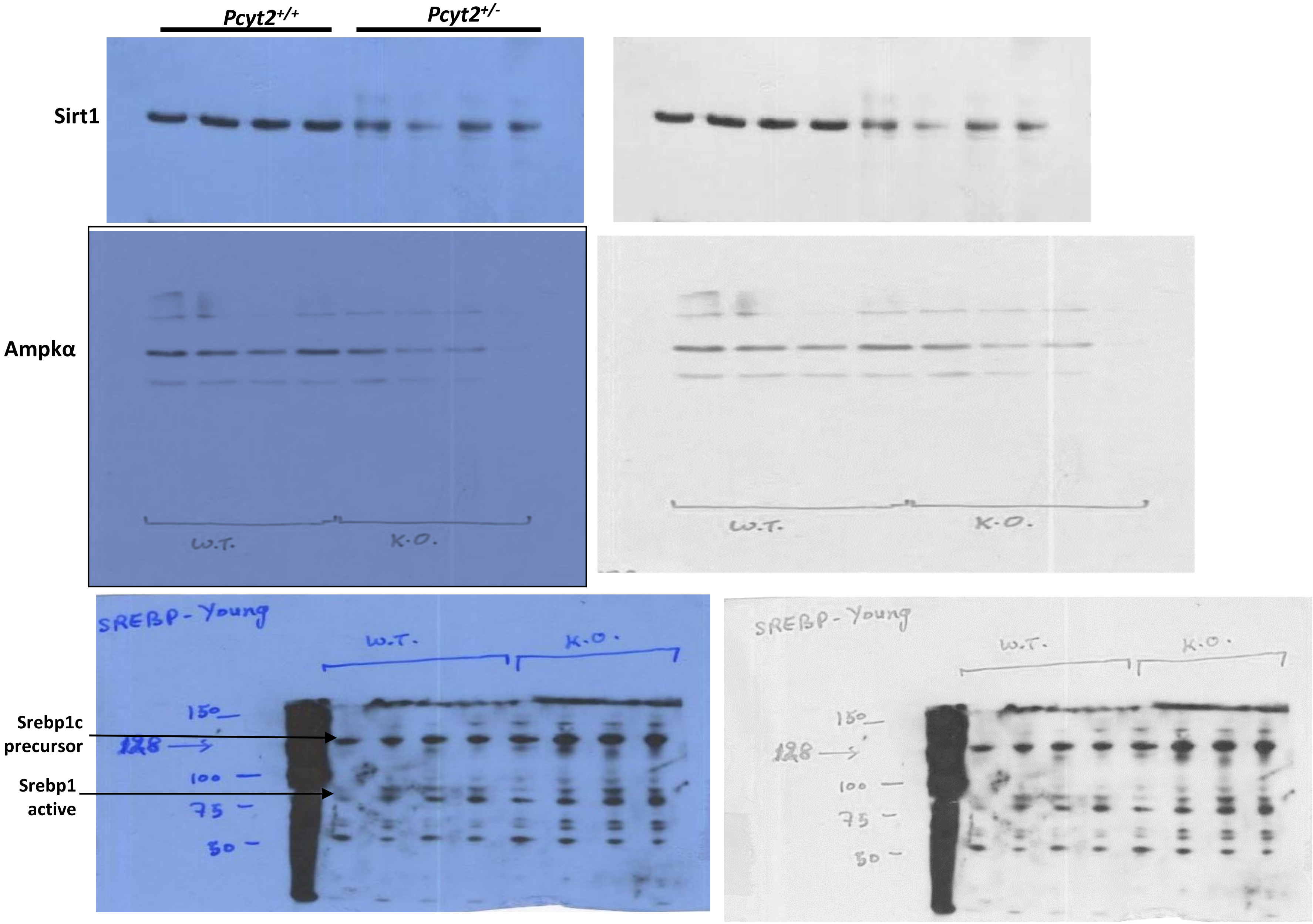

Figure 2D

ORIGINAL **Fasted**

Pcyt2$^{+/+}$ ***Pcyt2***$^{+/-}$

USED IN MANUSCRIPT

Acc

Pkcα

Pkcβ1

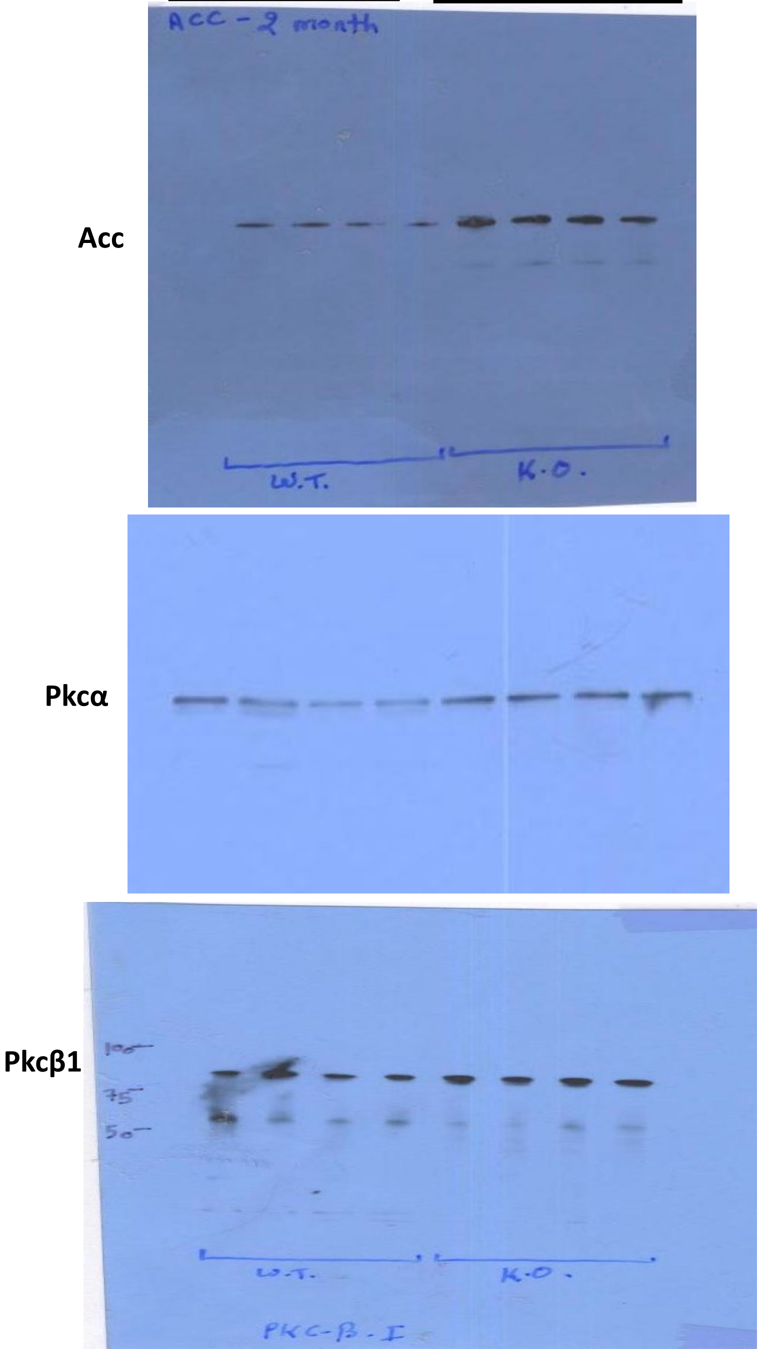

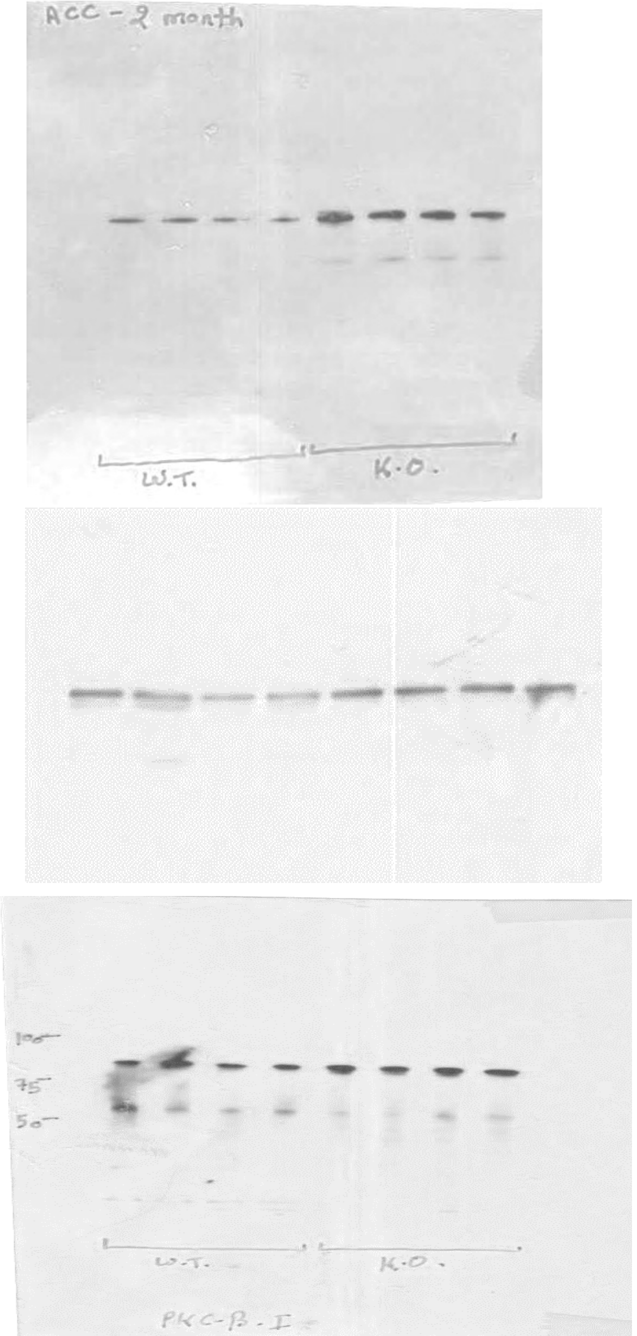

Figure 2D

ORIGINAL

USED IN MANUSCRIPT

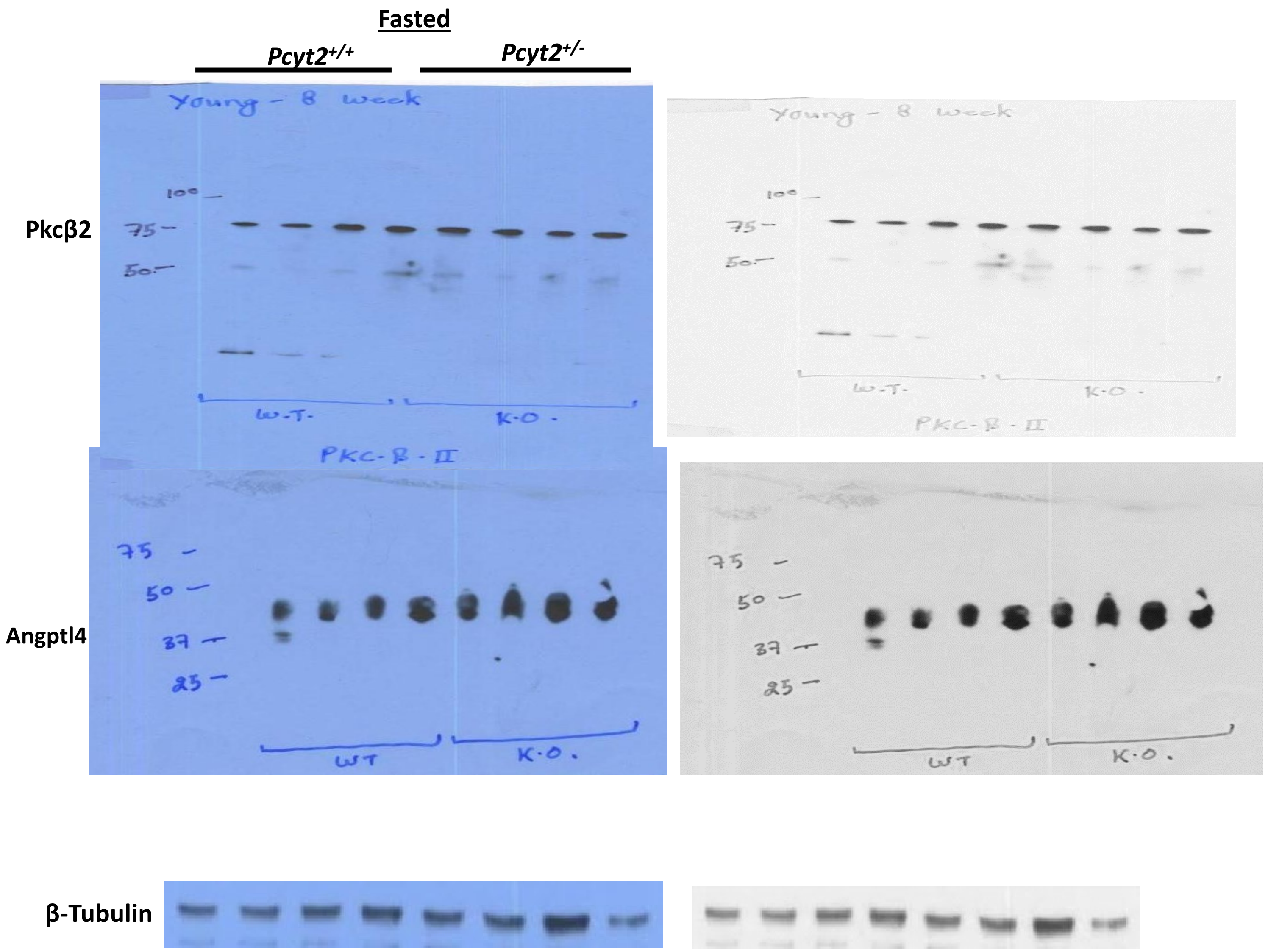

Figure 2D

ORIGINAL

USED IN MANUSCRIPT

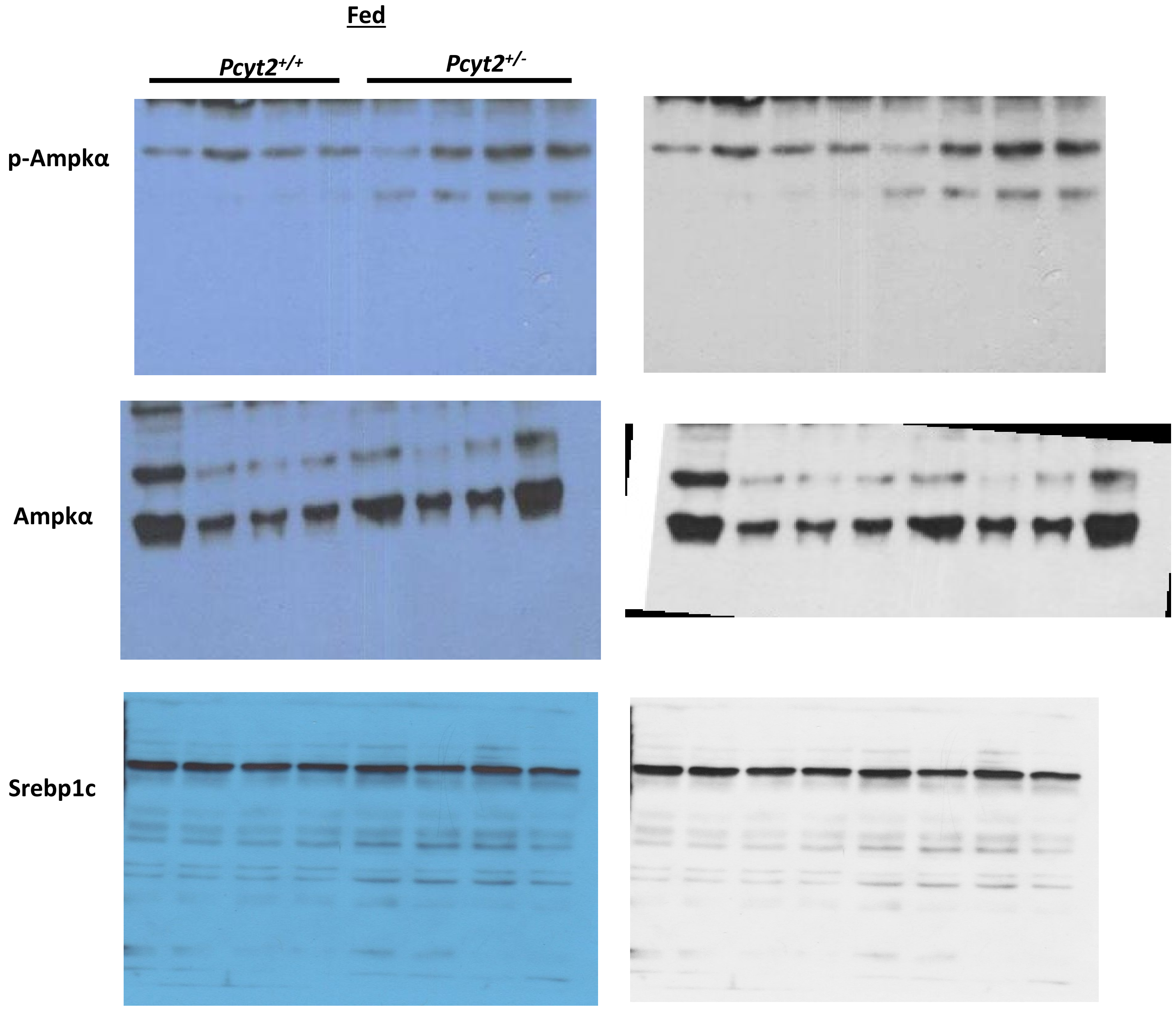

Figure 2D

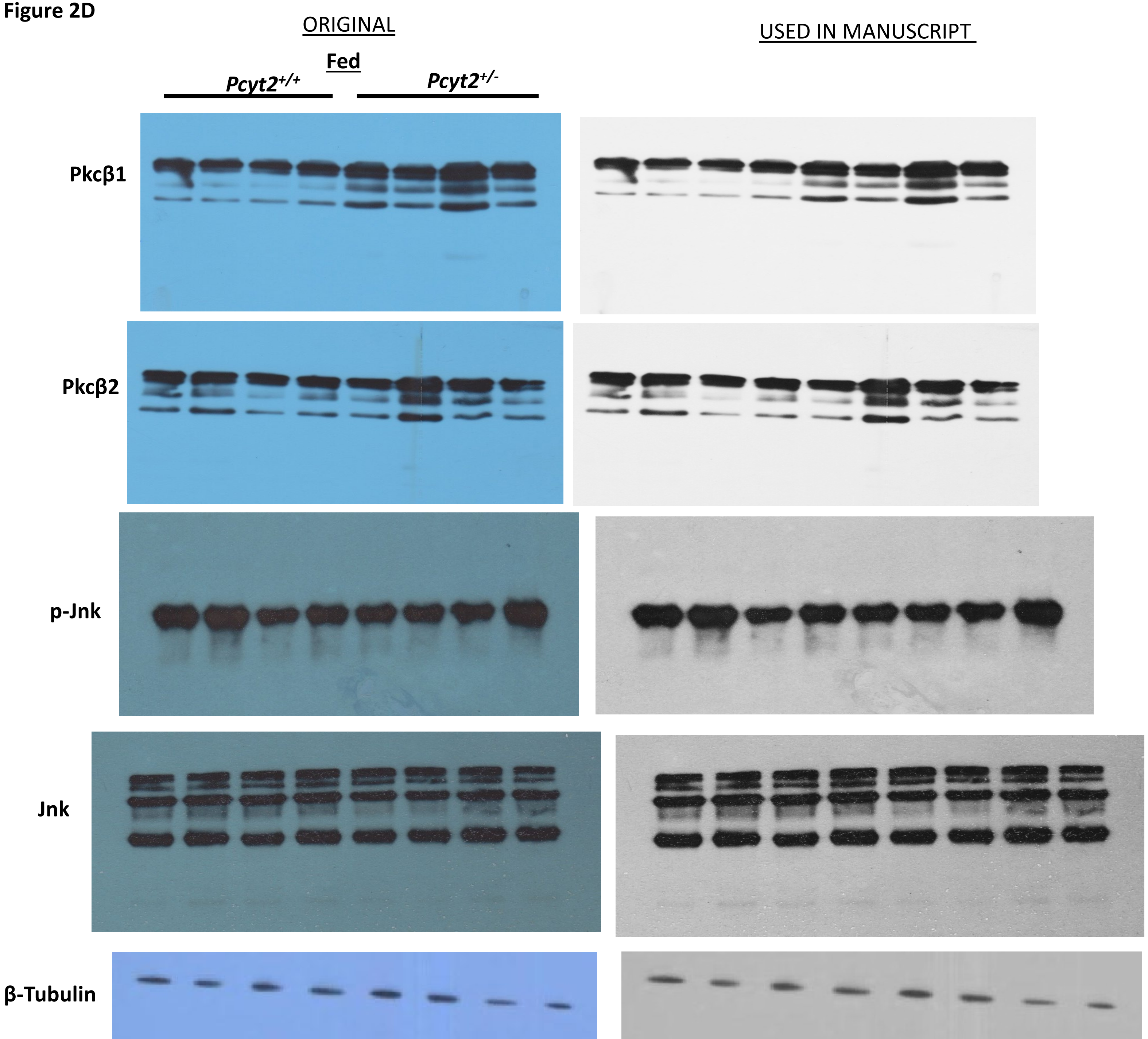
ORIGINAL
USED IN MANUSCRIPT
Fed
Pcyt2+/+
Pcyt2+/-
Pkcβ1
Pkcβ2
p-Jnk
Jnk
β-Tubulin

Figure 3C

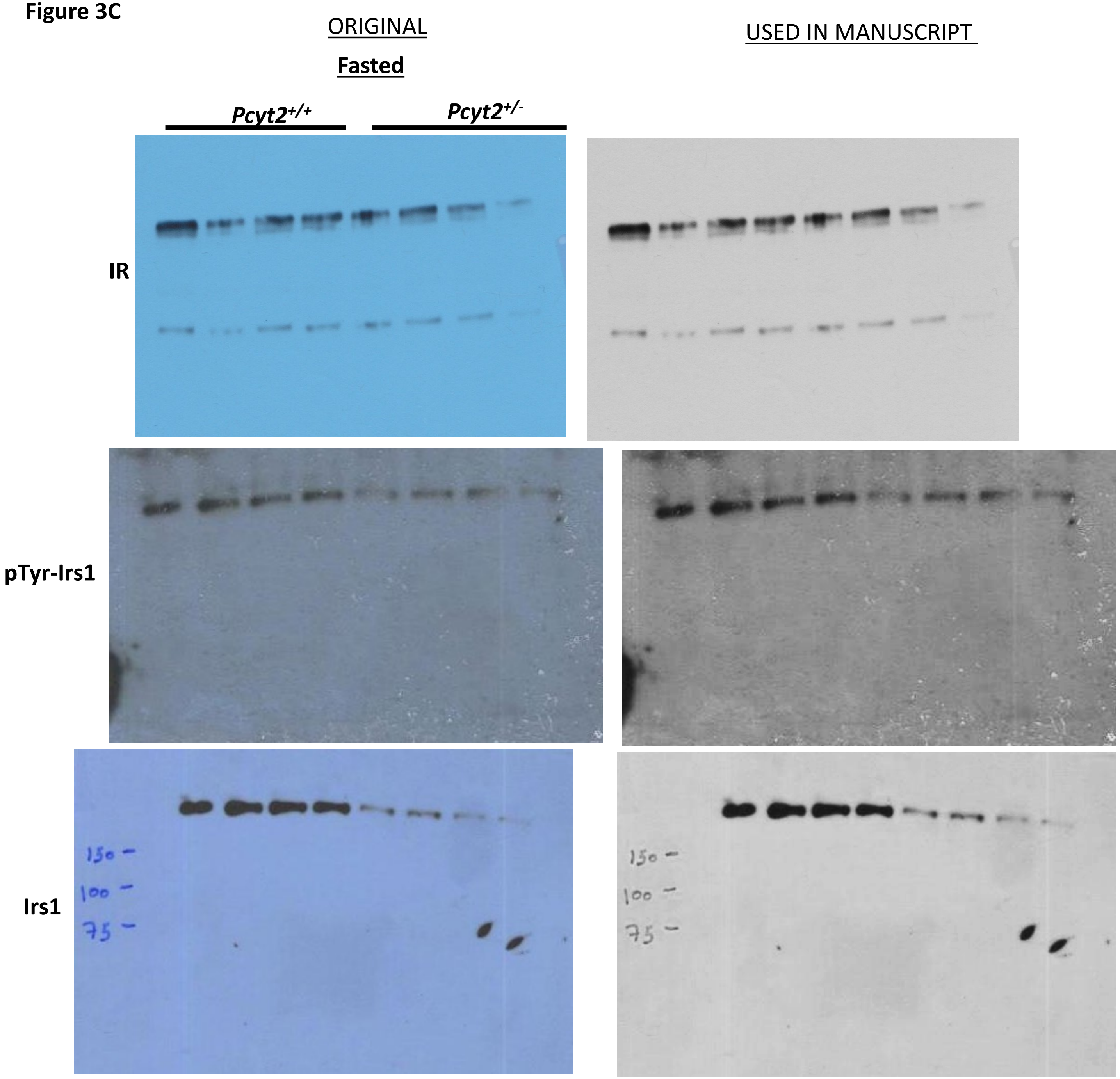
ORIGINAL
USED IN MANUSCRIPT
Fasted
Pcyt2+/+
Pcyt2+/-
IR
pTyr-Irs1
Irs1
150
100
75

Figure 3C

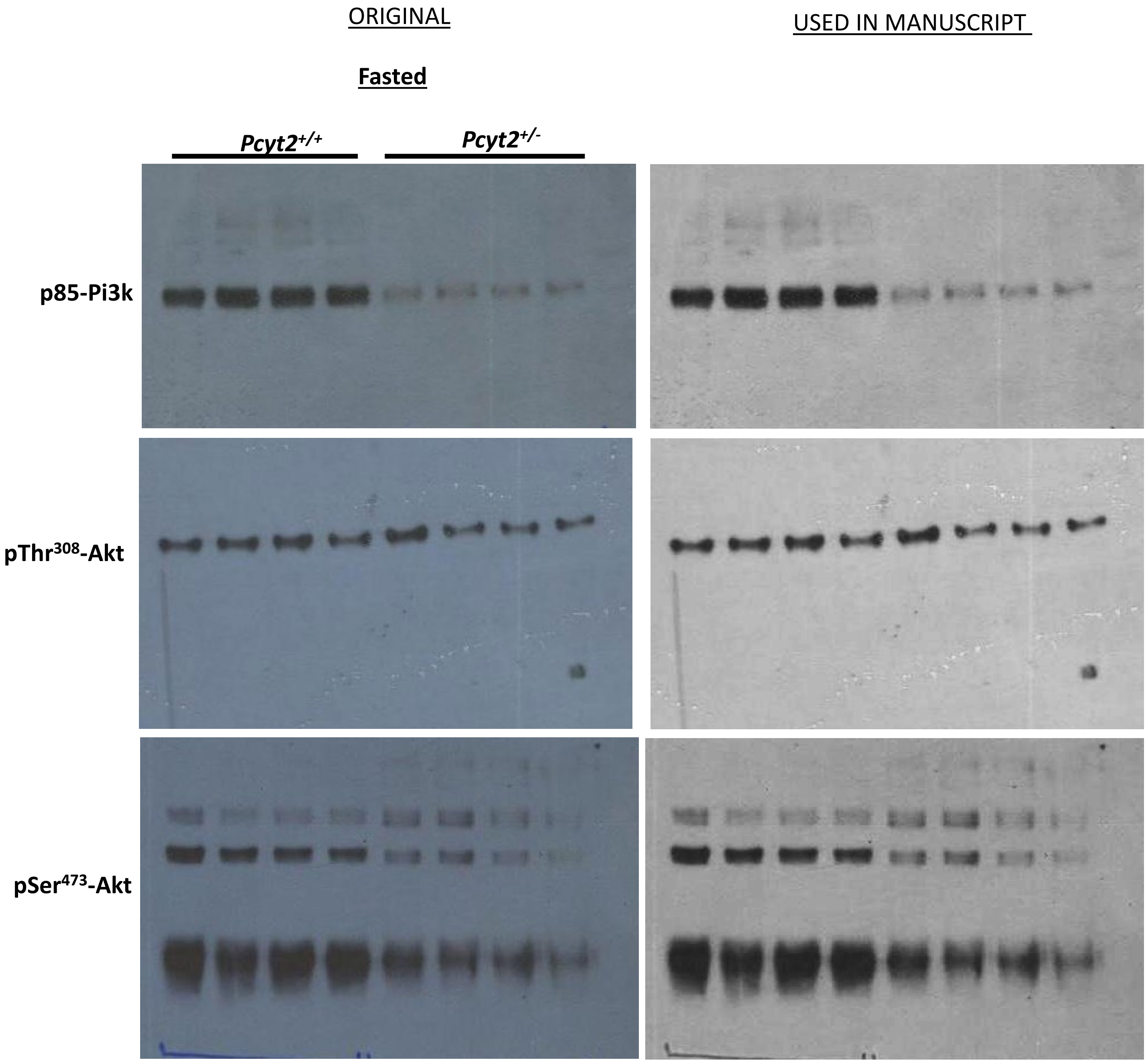
ORIGINAL
Fasted
Pcyt2+/+
Pcyt2+/-
USED IN MANUSCRIPT
p85-Pi3k
pThr308-Akt
pSer473-Akt

Figure 3C

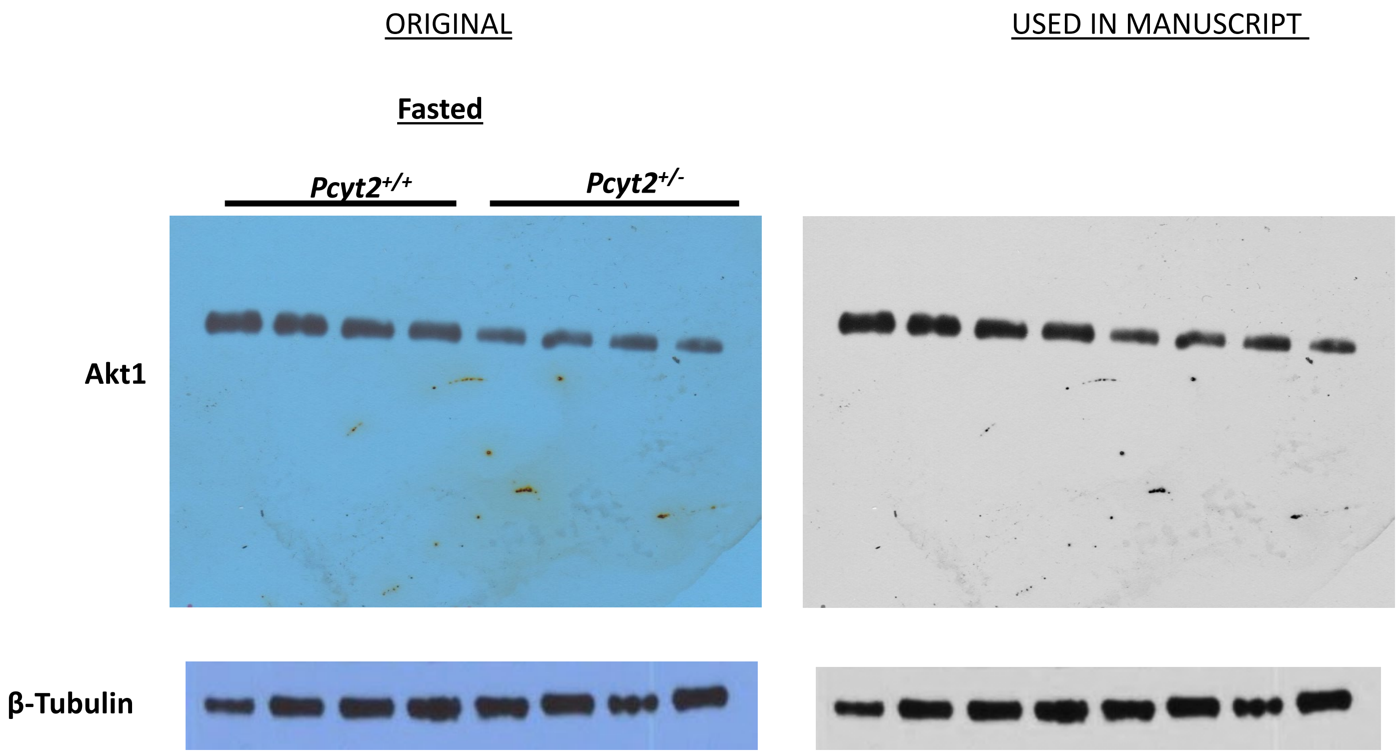
ORIGINAL
USED IN MANUSCRIPT
Fasted
Pcyt2+/+
Pcyt2+/-
Akt1
β-Tubulin

Figure 3C

ORIGINAL

USED IN MANUSCRIPT

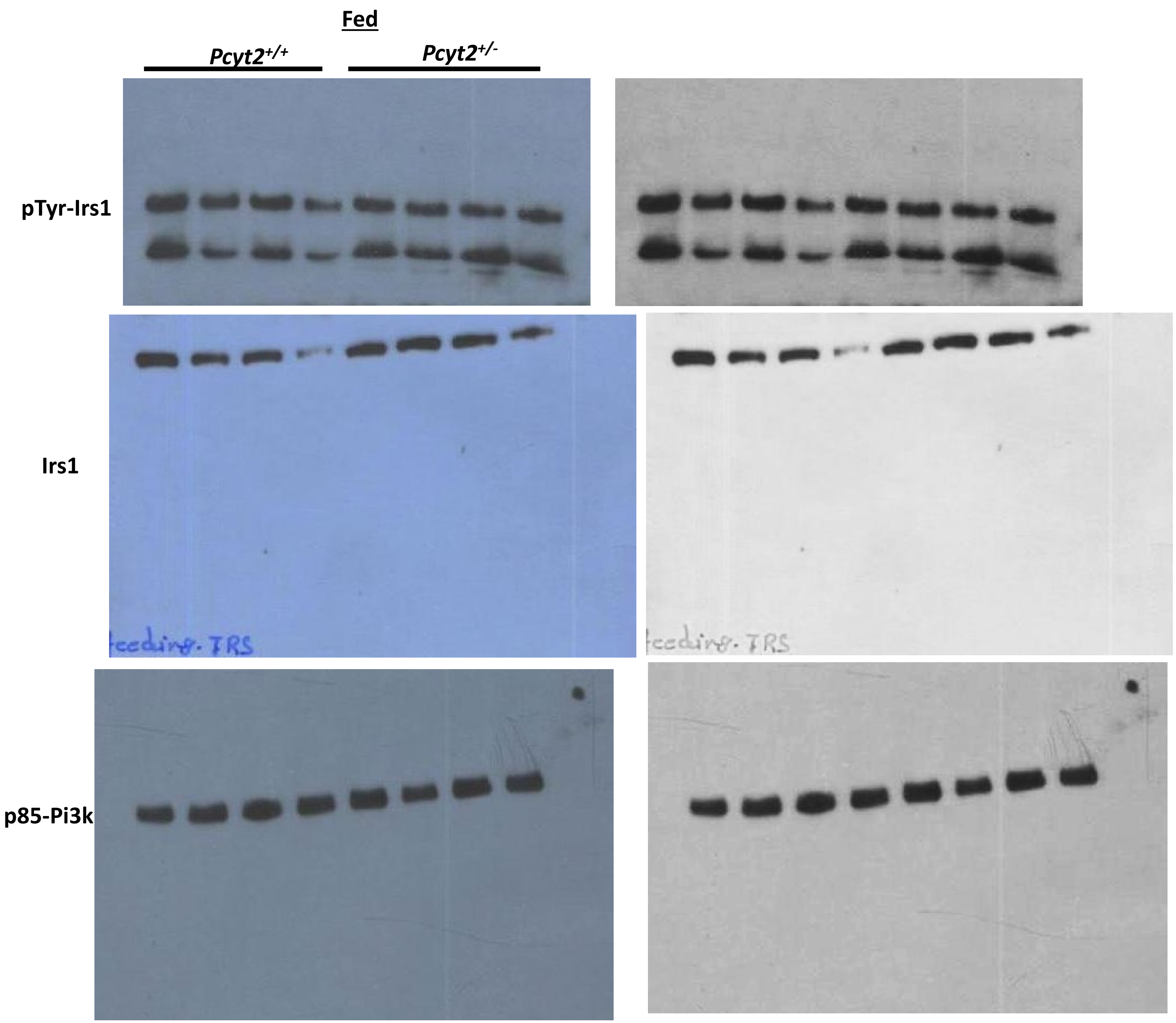

Figure 3C

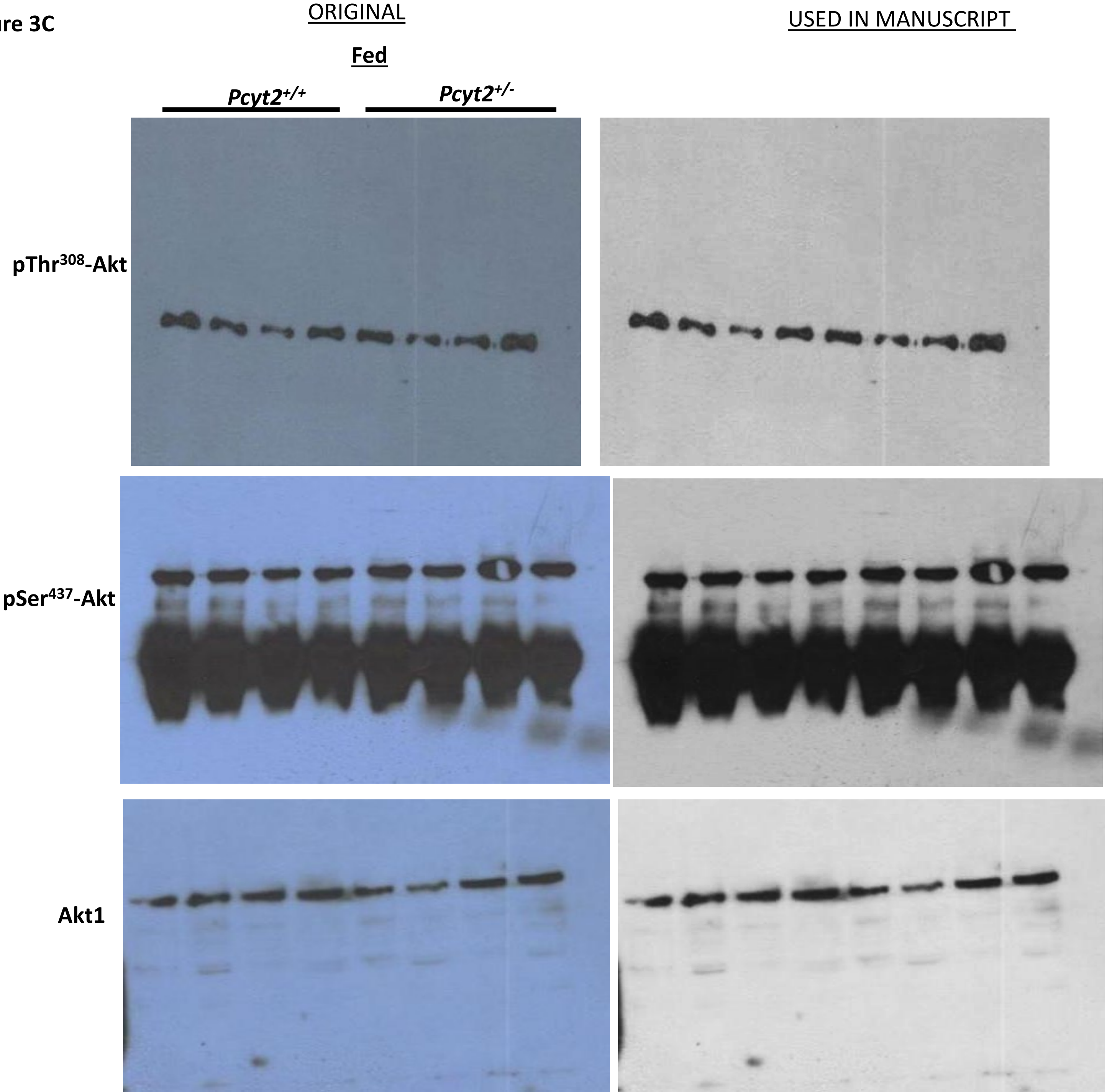
ORIGINAL
USED IN MANUSCRIPT
Fed
Pcyt2+/+
Pcyt2+/-
pThr308-Akt
pSer437-Akt
Akt1

Figure 3C

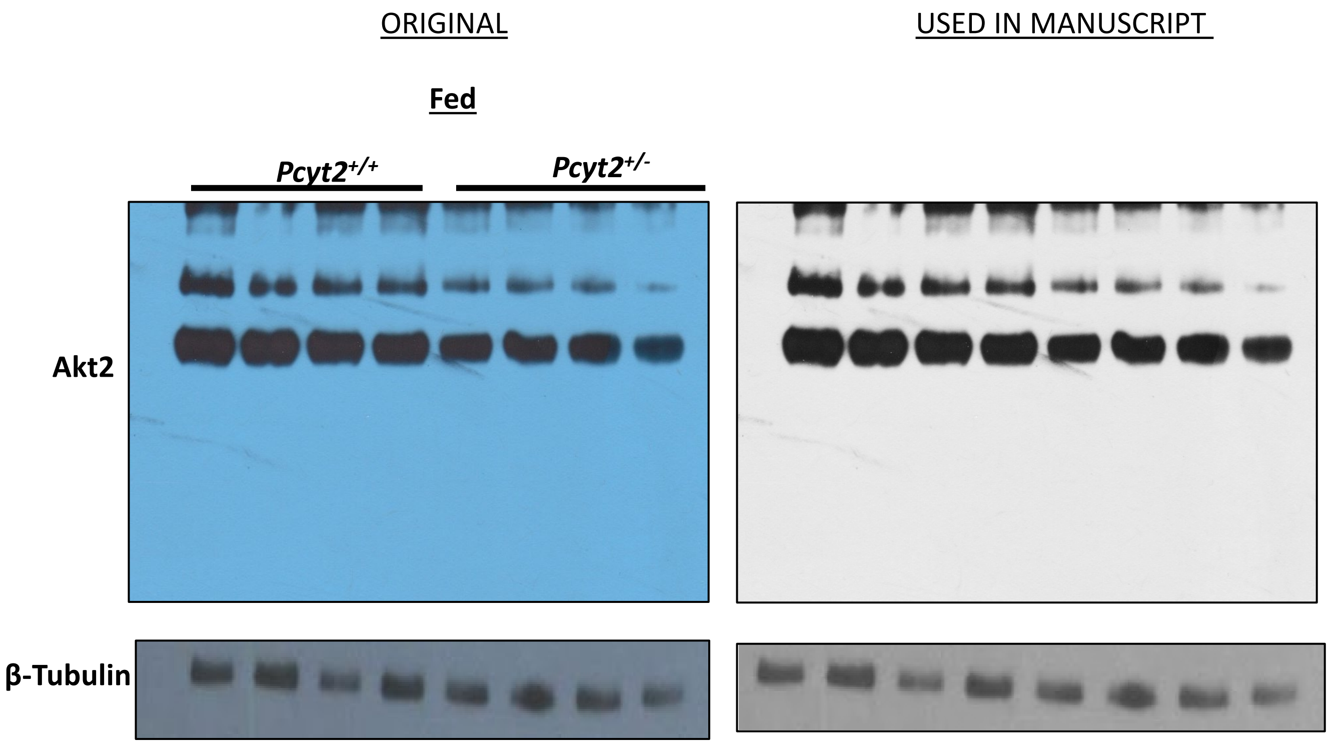

Supplementary Figure 1 ORIGINAL USED IN MANUSCRIPT

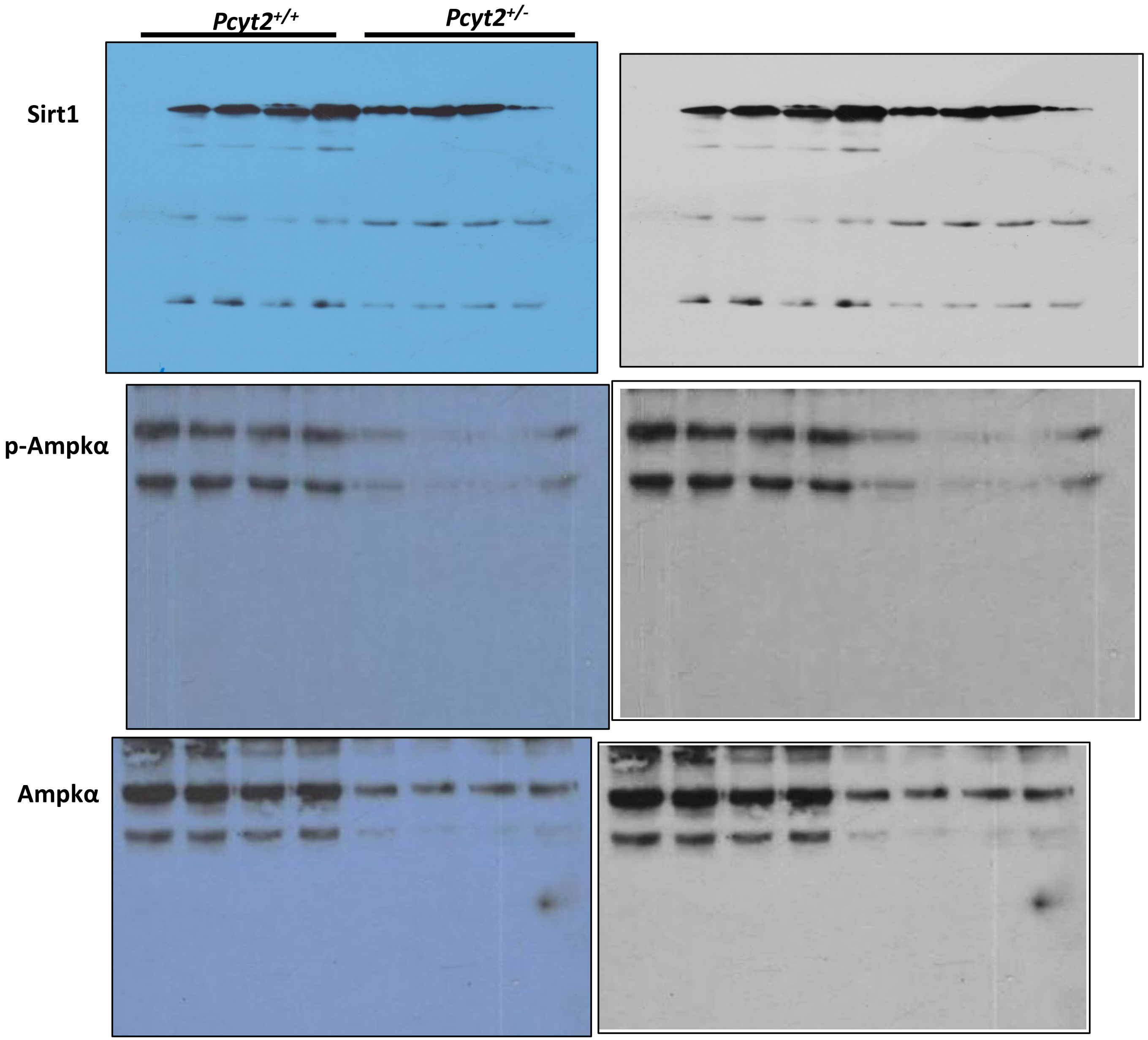

Supplementary Figure 1

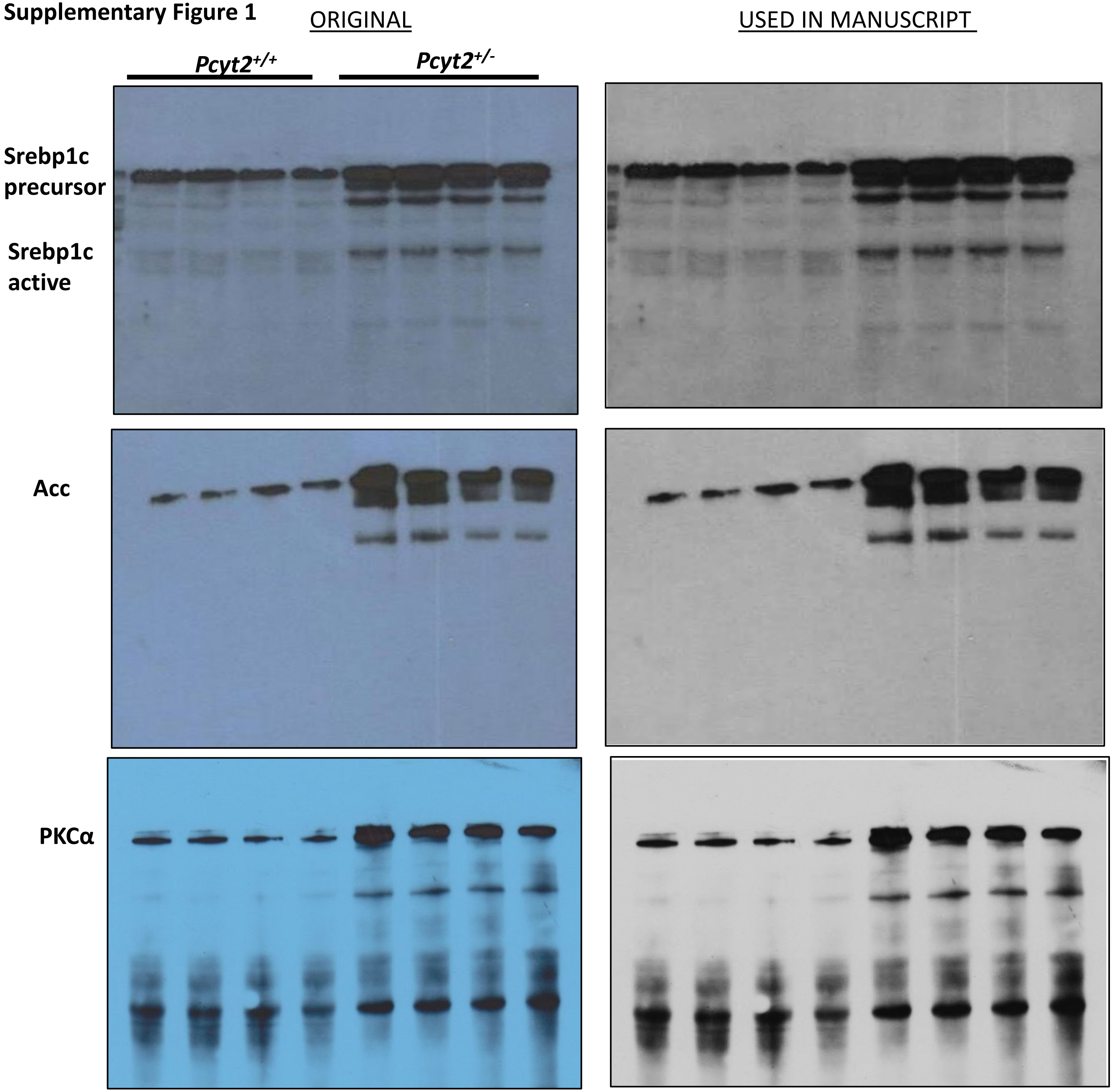

Supplementary Figure 1

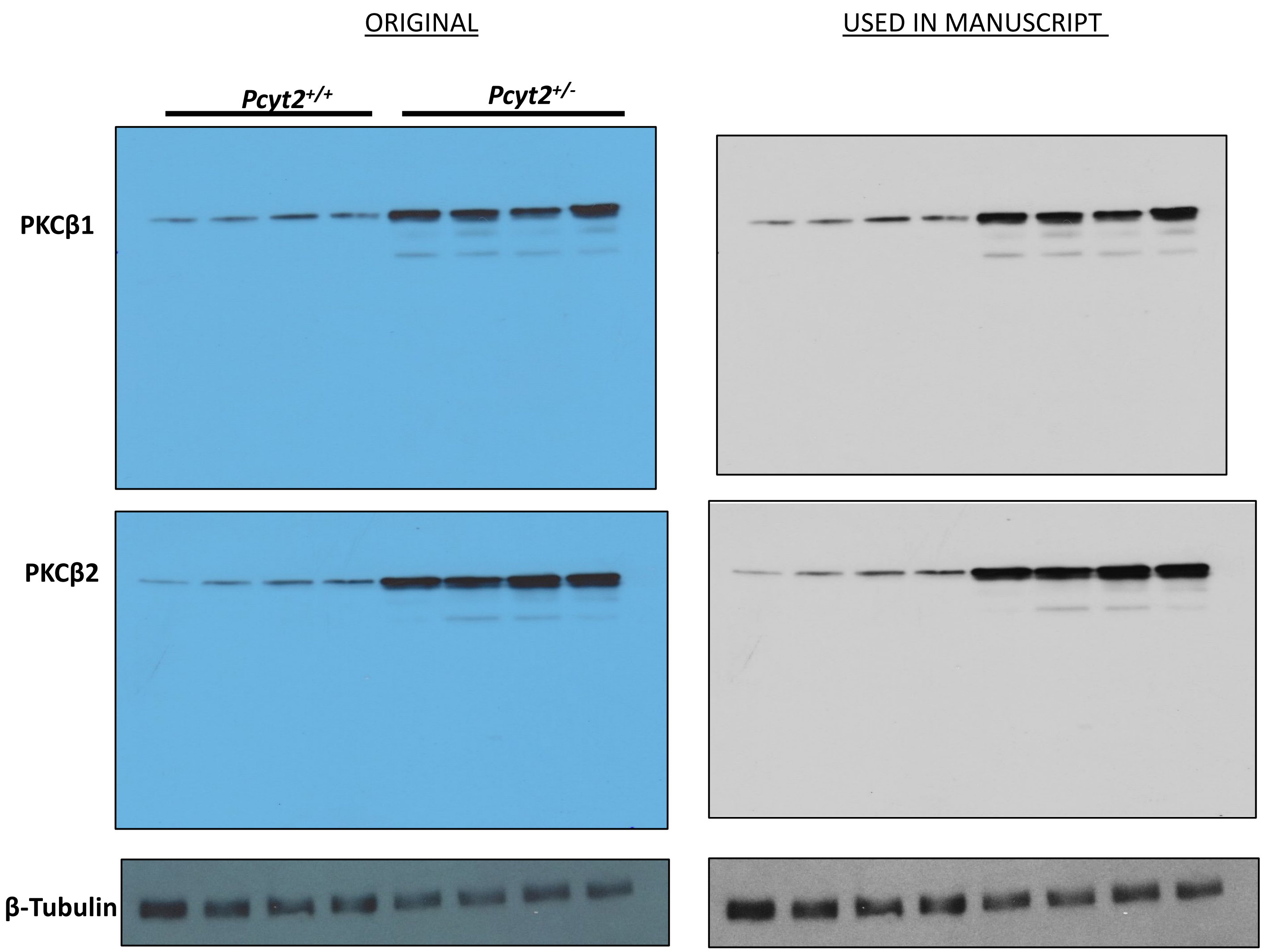

Figure 4A

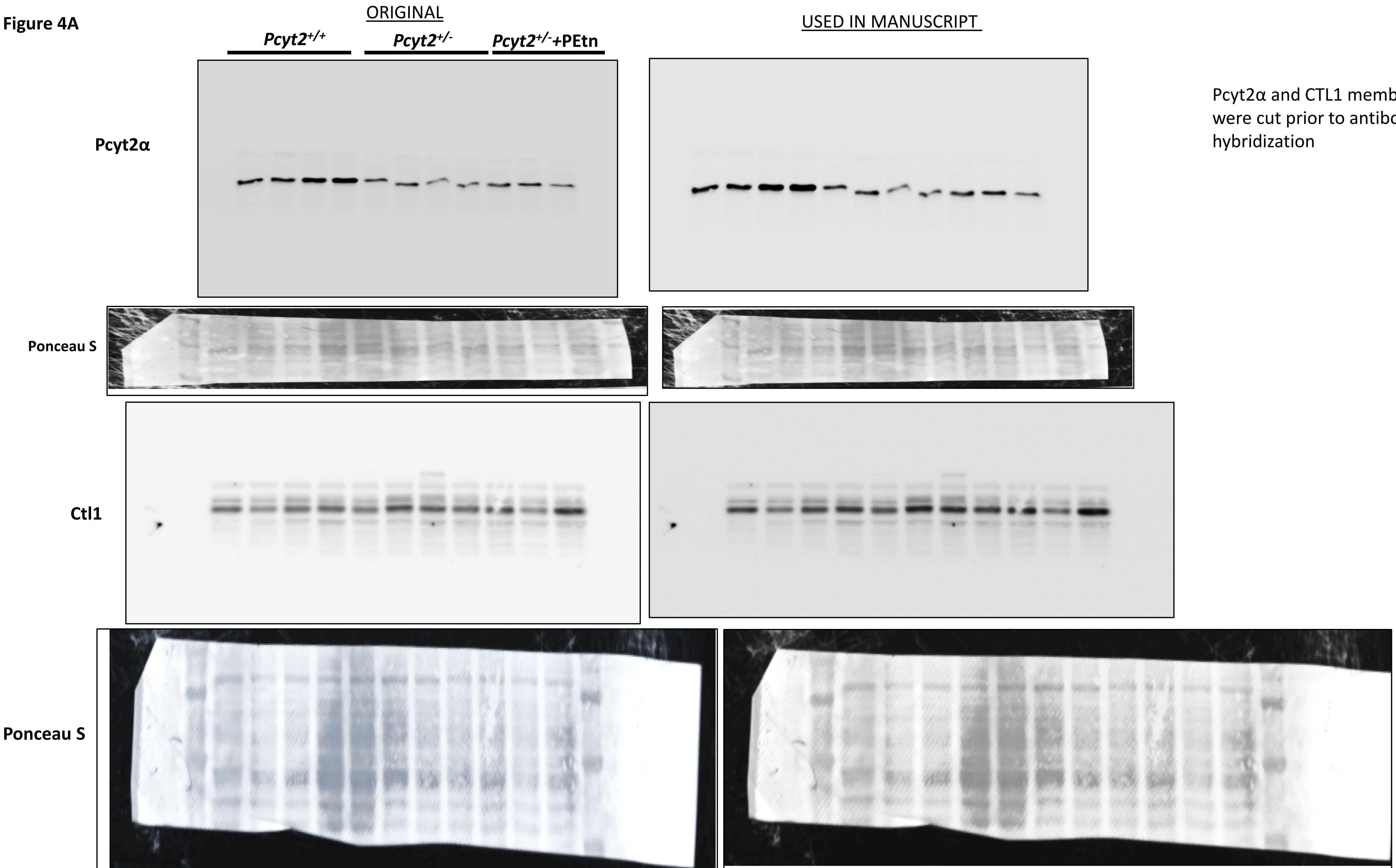

Pcyt2α and CTL1 membranes were cut prior to antibody hybridization

Figure 5A Pkcα and Pkcβ1 membranes were cut prior to antibody hybridization

ORIGINAL USED IN MANUSCRIPT

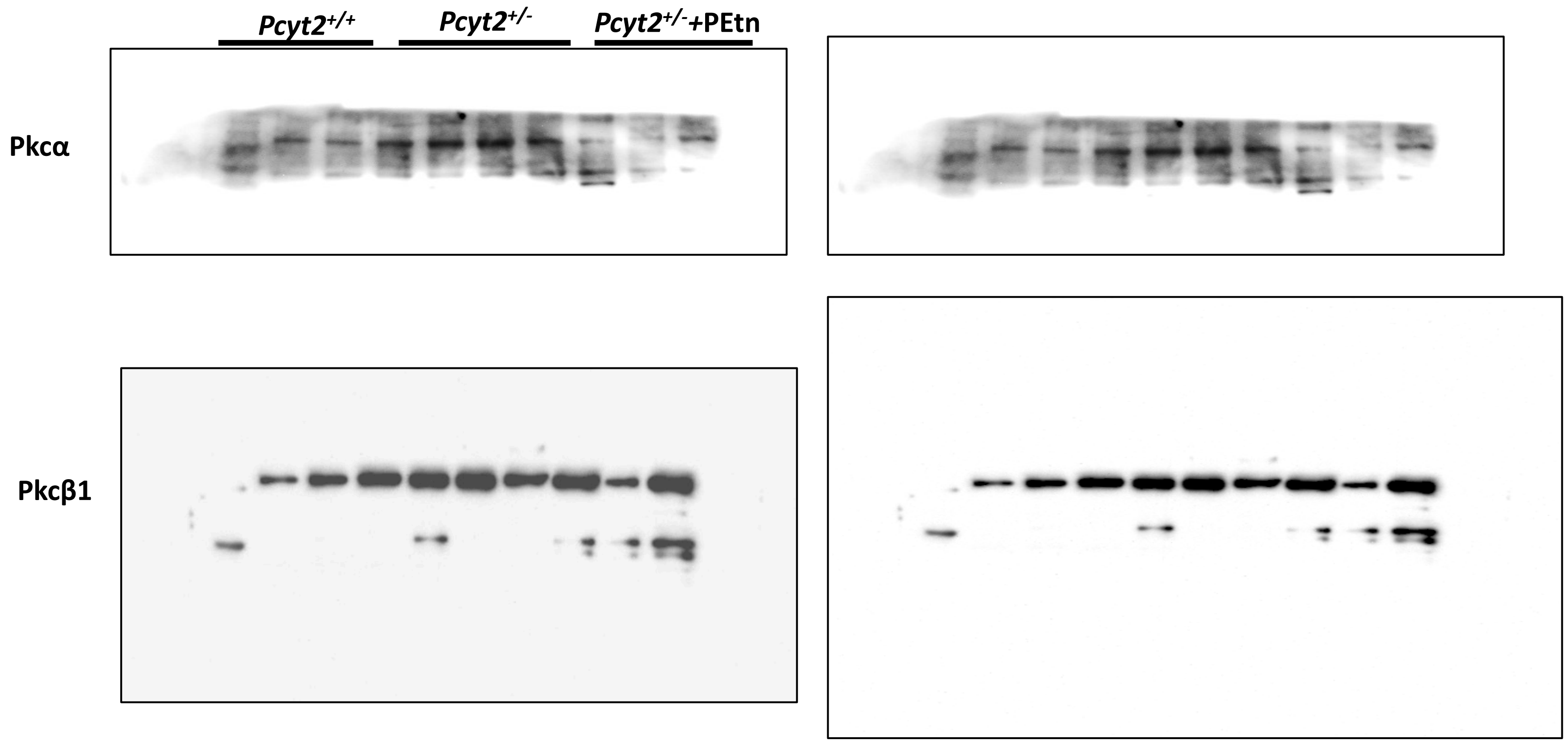

Figure 5A

p-Foxo1 and β-Actin **m**embranes were cut prior to antibody hybridization

ORIGINAL

USED IN MANUSCRIPT

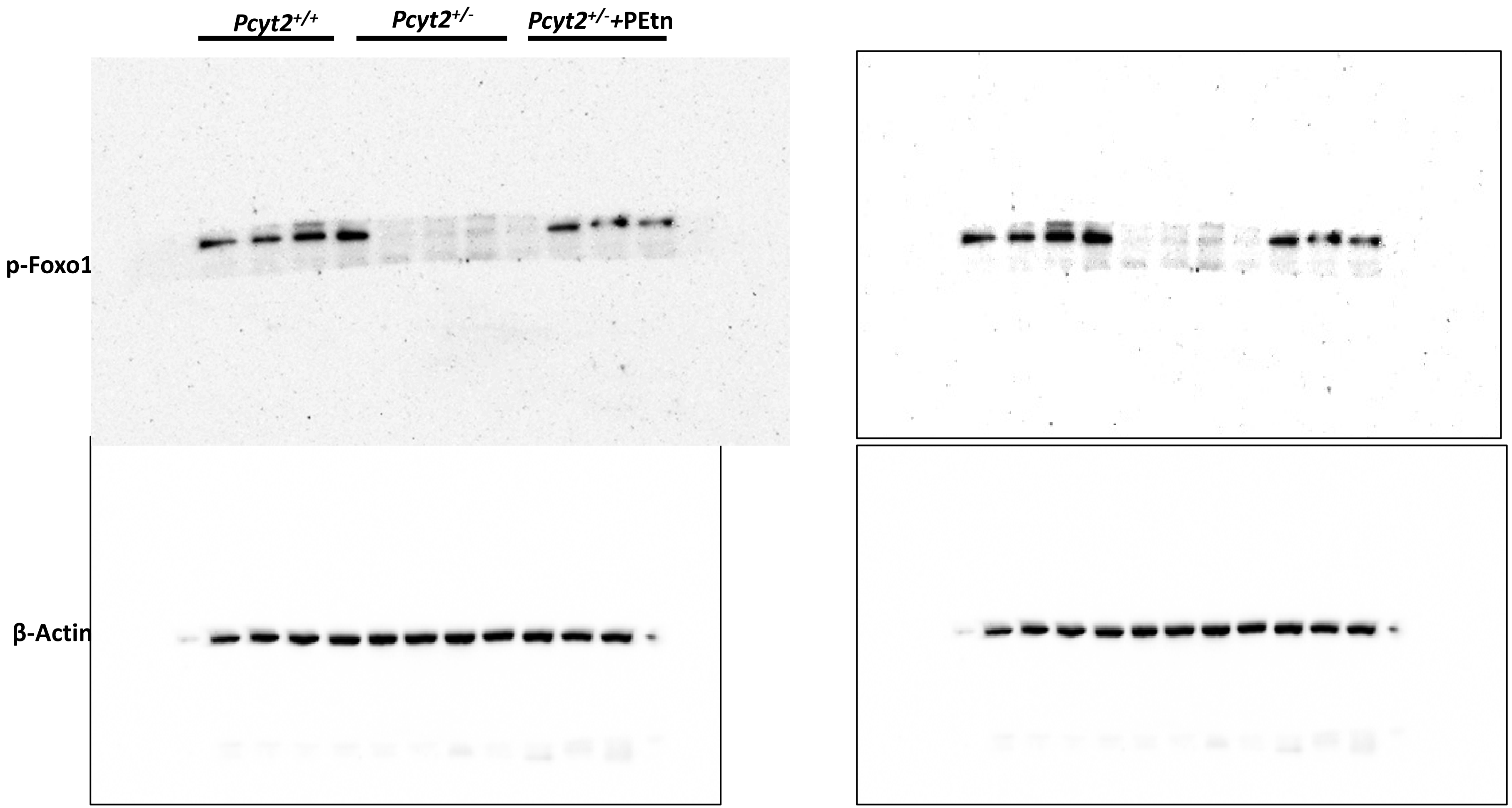

Figure 5B

Sirt1, Pgc1α and p-Ampkα membranes were cut prior to antibody hybridization

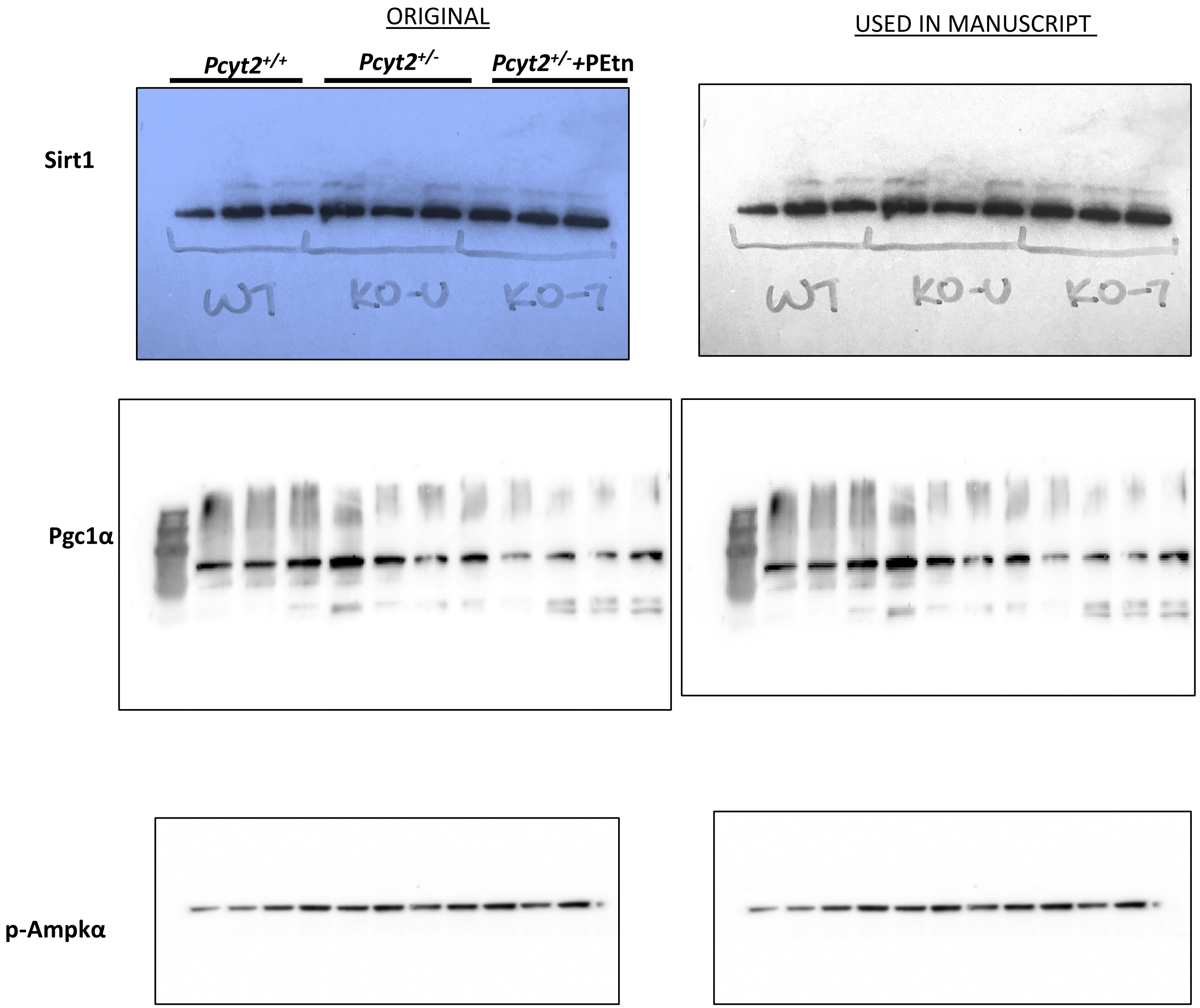

Ampkα and p-p70 S6K membranes were cut prior to antibody hybridization

Figure 5B

ORIGINAL

USED IN MANUSCRIPT

Pcyt2$^{+/+}$ ***Pcyt2***$^{+/-}$ ***Pcyt2***$^{+/-}$**+PEtn**

Ampkα

p-p70 S6K

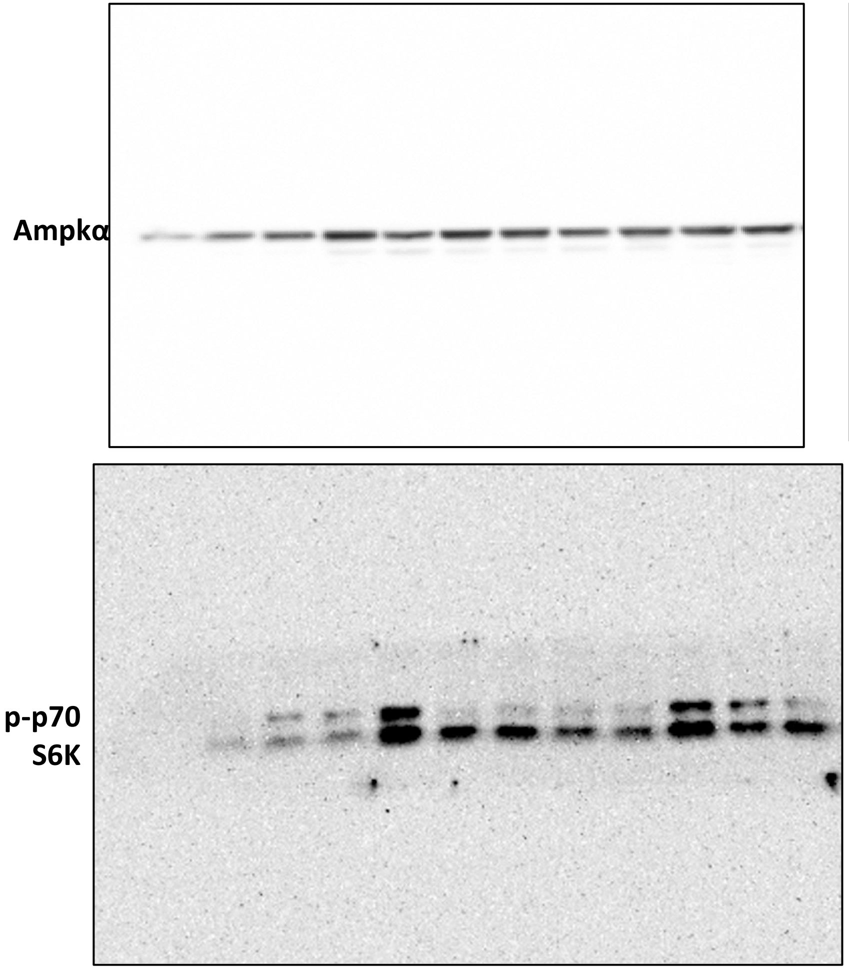

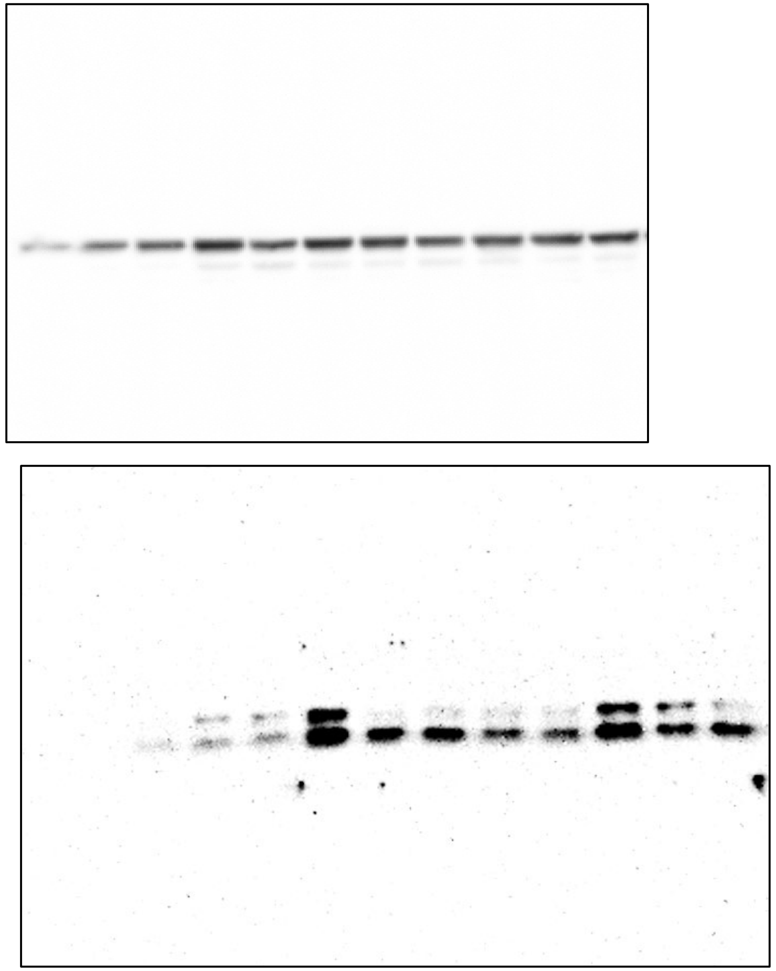

Figure 5B

mTorc1 and B-Actin membranes were cut prior to antibody hybridization

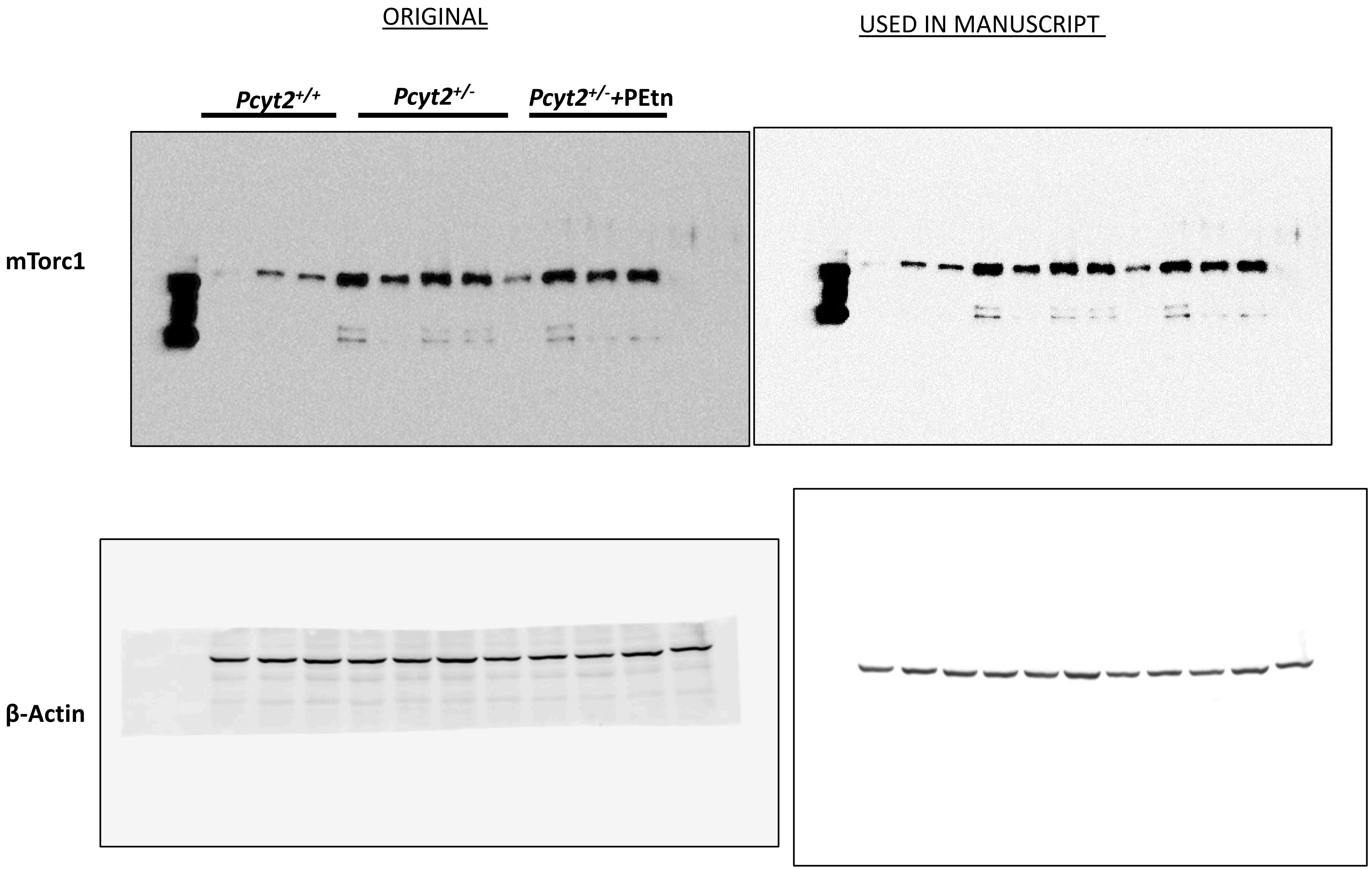

Figure 6C

ORIGINAL

USED IN MANUSCRIPT

Traf6

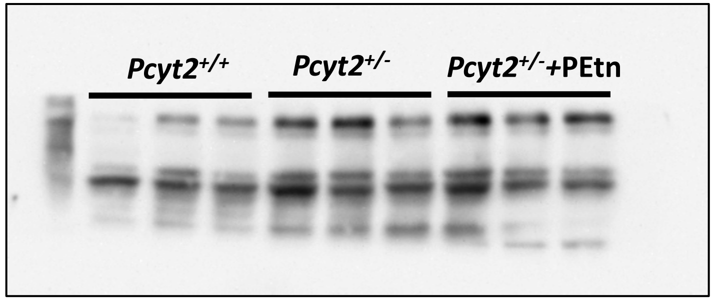

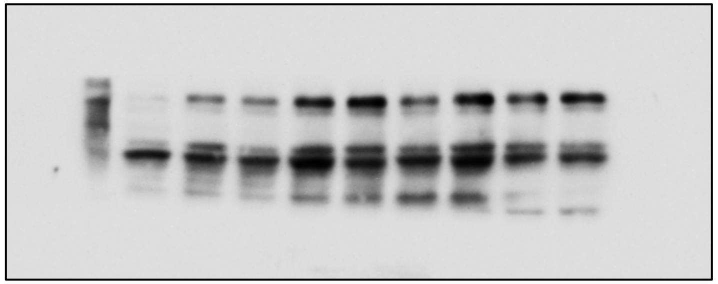

Traf6, Nfκb membranes were cut prior to antibody hybridization

Nfκb

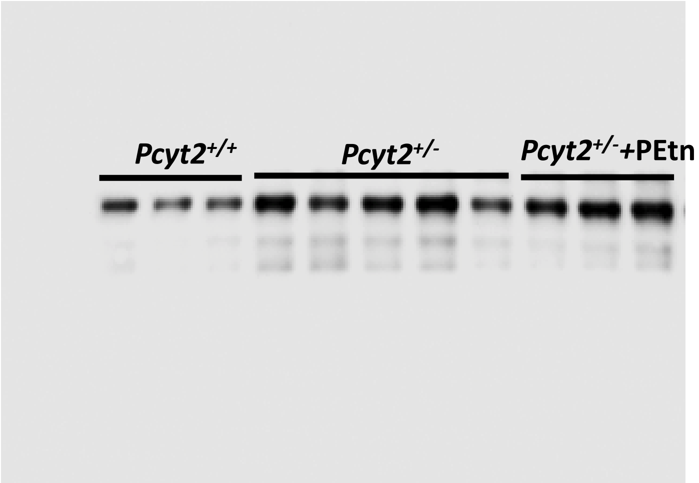

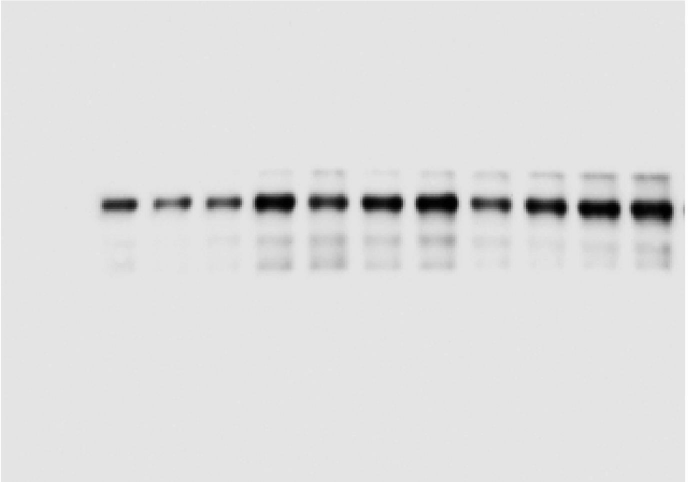

Nfκb, nuclear

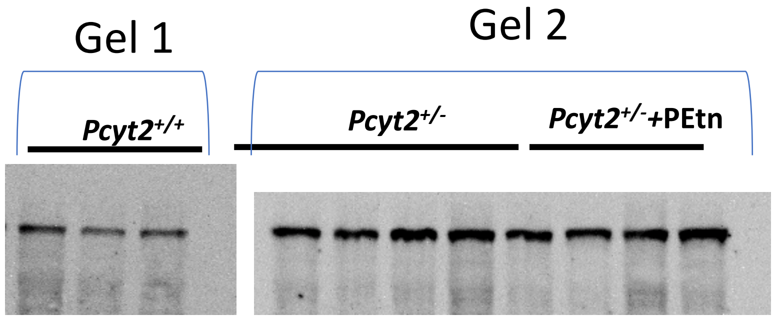

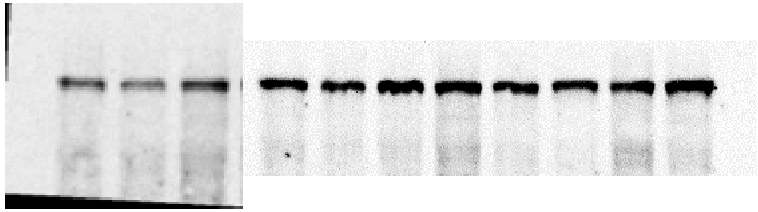

WB run on two separate mini-gels 1 and 2 but simultaneously; data are not used for quantifications

Figure 6C

Socs3, Stat3, and Stat3 nuclear membranes were cut prior to antibody hybridization

ORIGINAL

USED IN MANUSCRIPT

Socs3

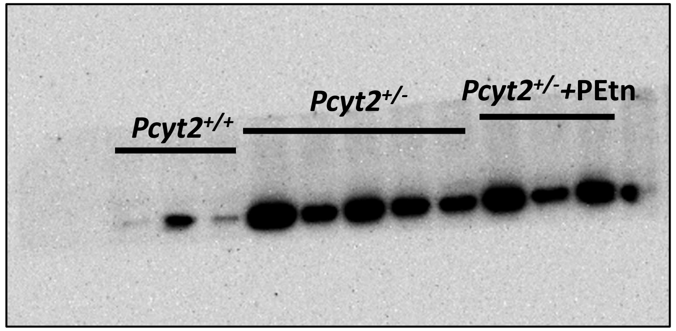

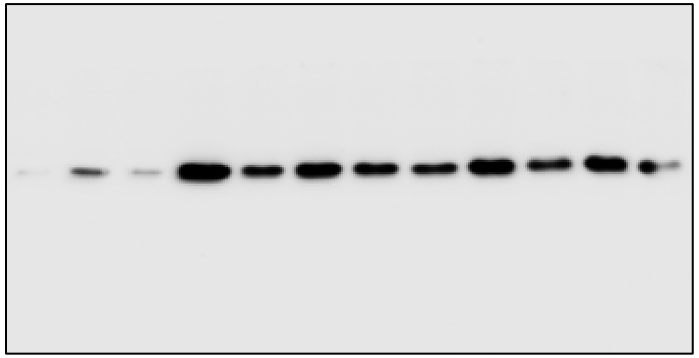

Stat3

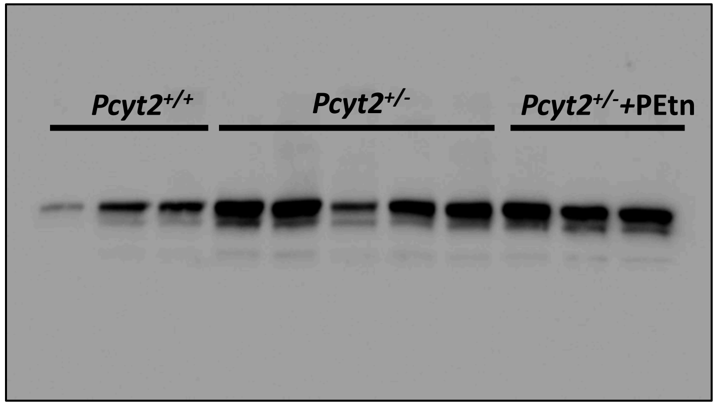

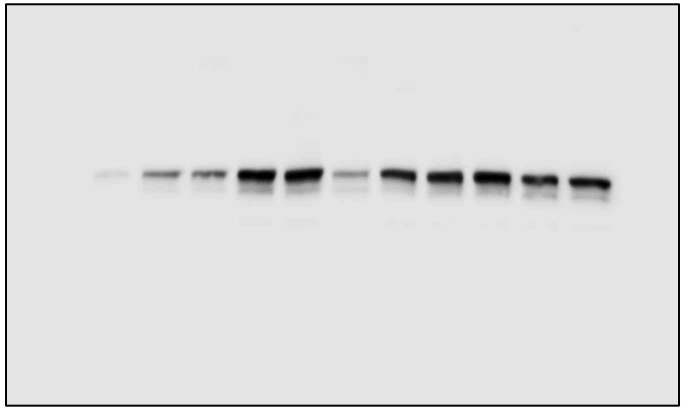

Stat3, nuclear

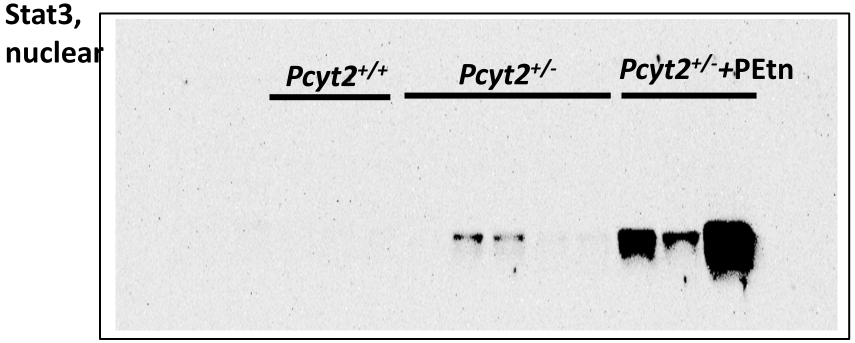

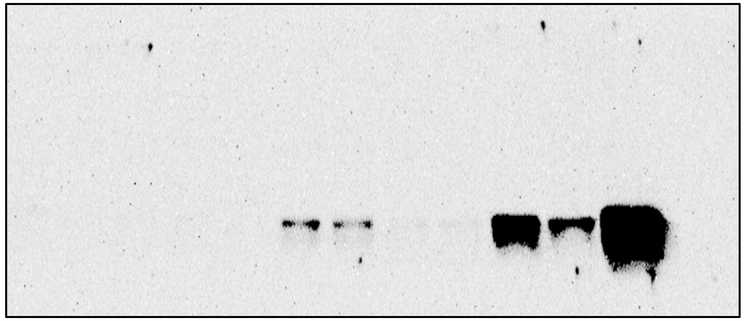

Figure 6C

β-Tubulin membrane was cut prior to antibody hybridization

ORIGINAL

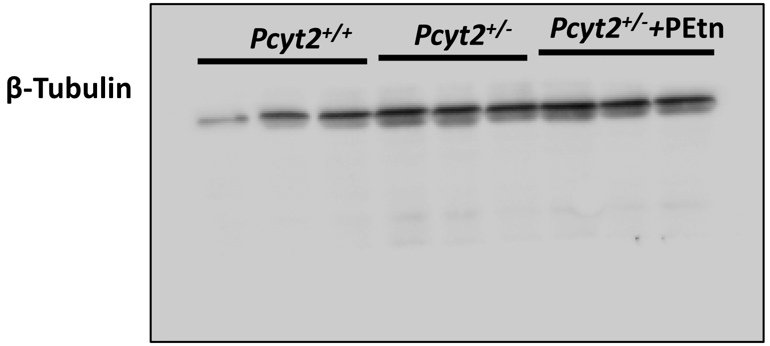

USED IN MANUSCRIPT

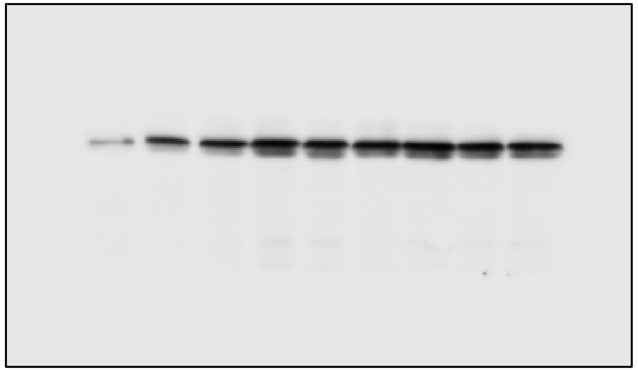

Keap1, Nrf2 and Nrf2 nuclear membrane were cut prior to antibody hybridization

Figure 6D

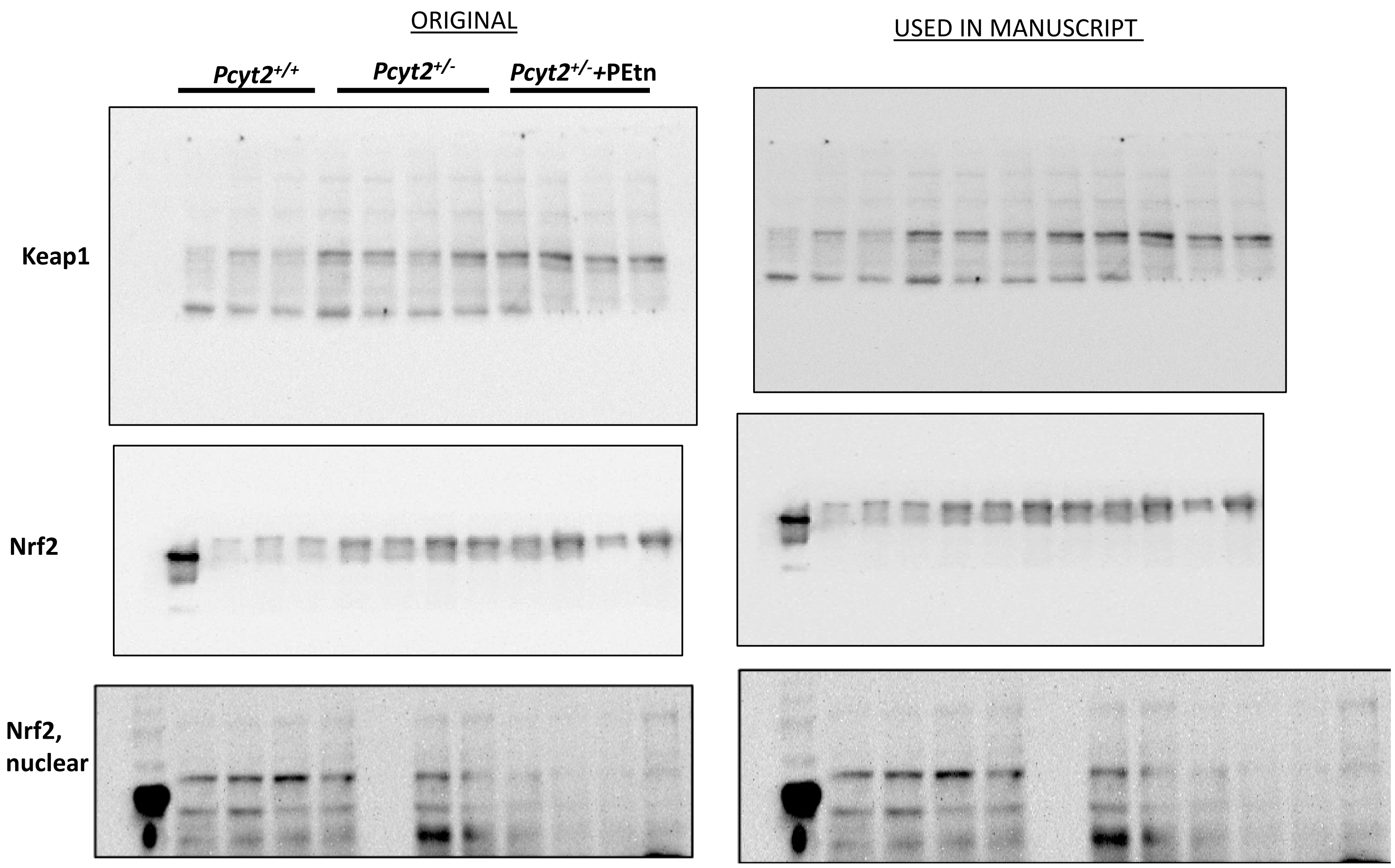

Figure 6D

p-Eif2α, Eif2α and β-Actin membrane were cut prior to antibody hybridization

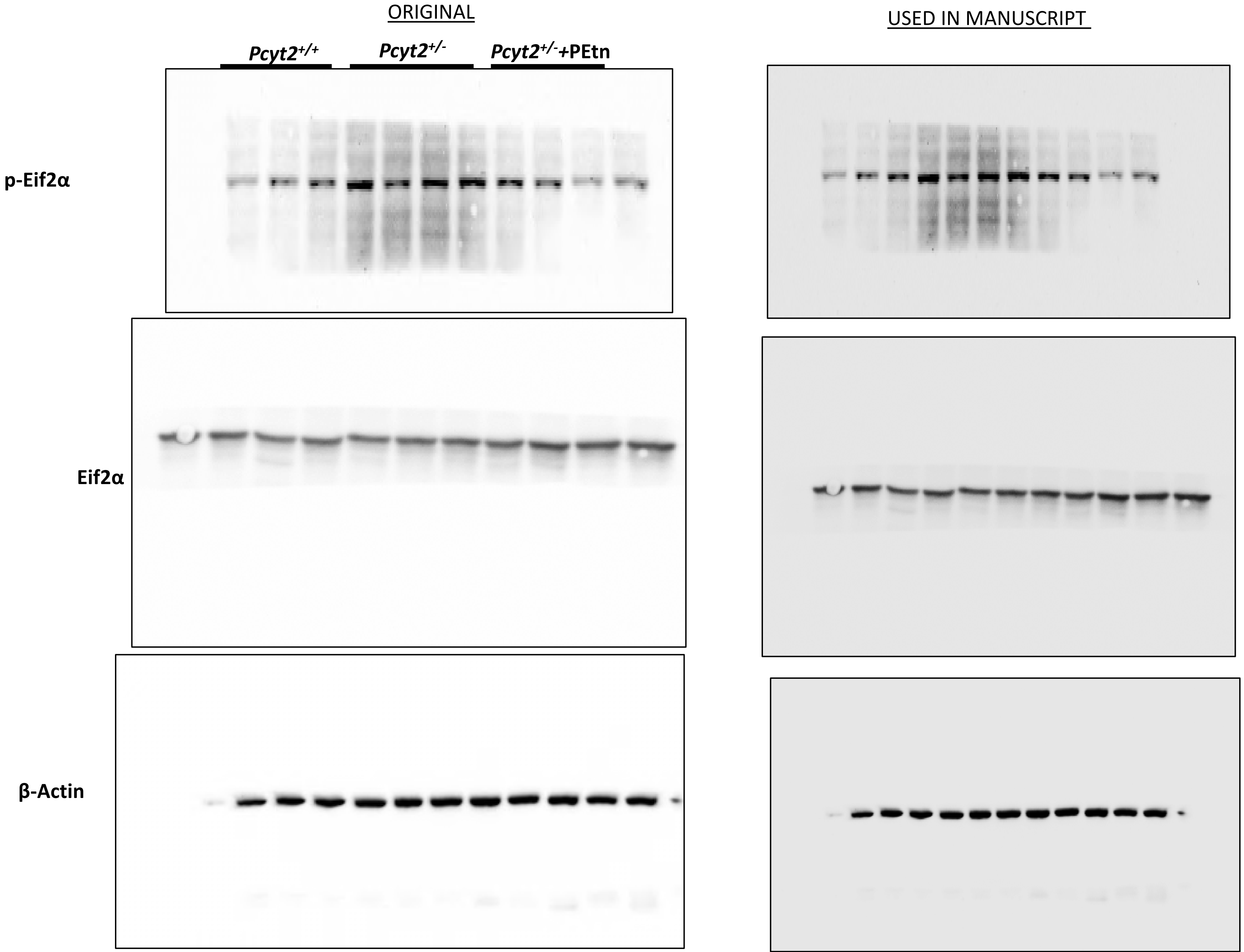

Figure 6E

p-Erk1/2, Erk1/2 and p-Jnk1/2 membranes were cut prior to antibody hybridization

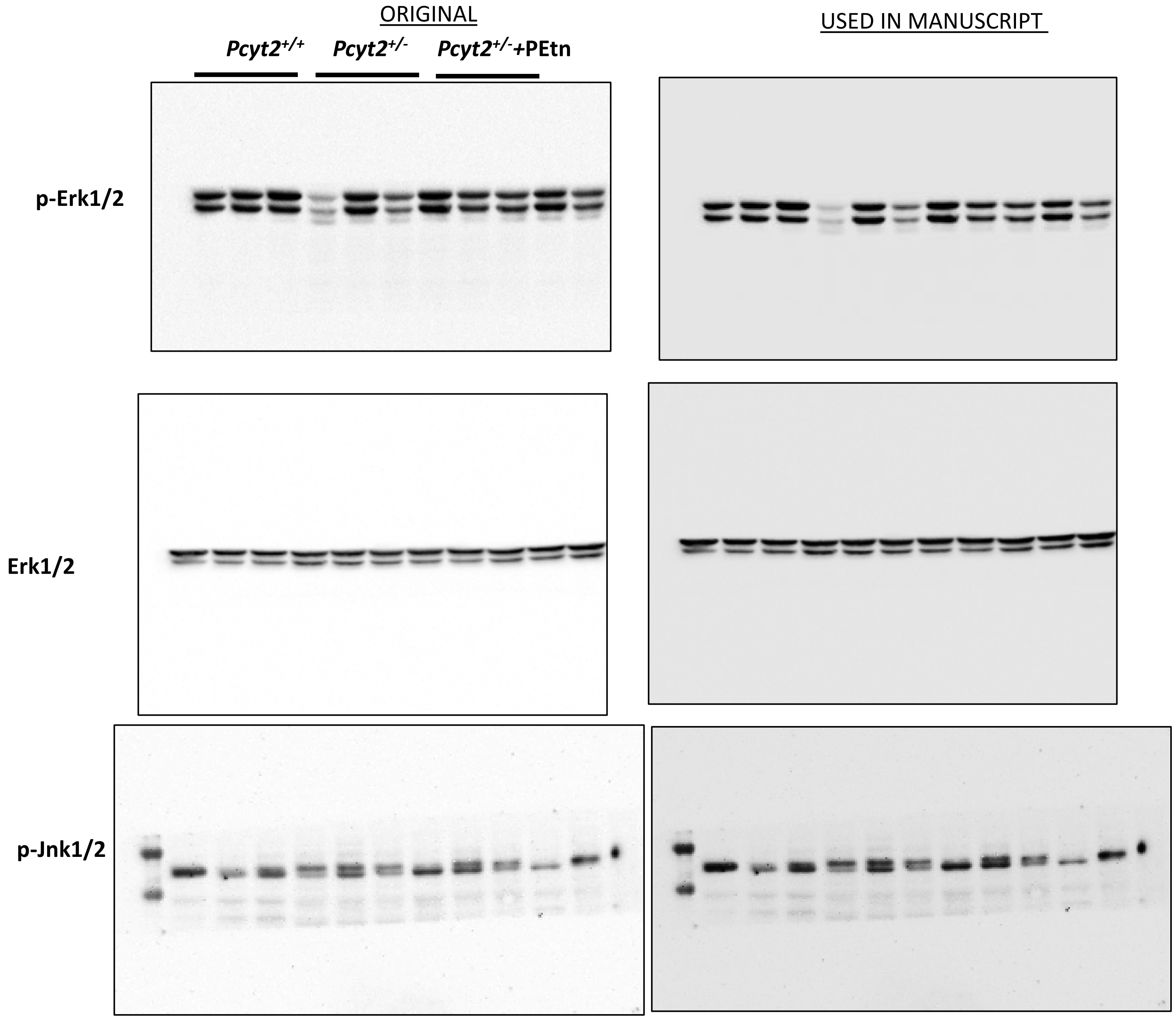

Jnk1/2, p-p38 and p38 membranes were cut prior to antibody hybridization

Figure 6E

ORIGINAL

USED IN MANUSCRIPT

$Pcyt2^{+/+}$ ***$Pcyt2^{+/-}$*** ***$Pcyt2^{+/-}$*+PEtn**

Jnk1/2

p-p38

p38

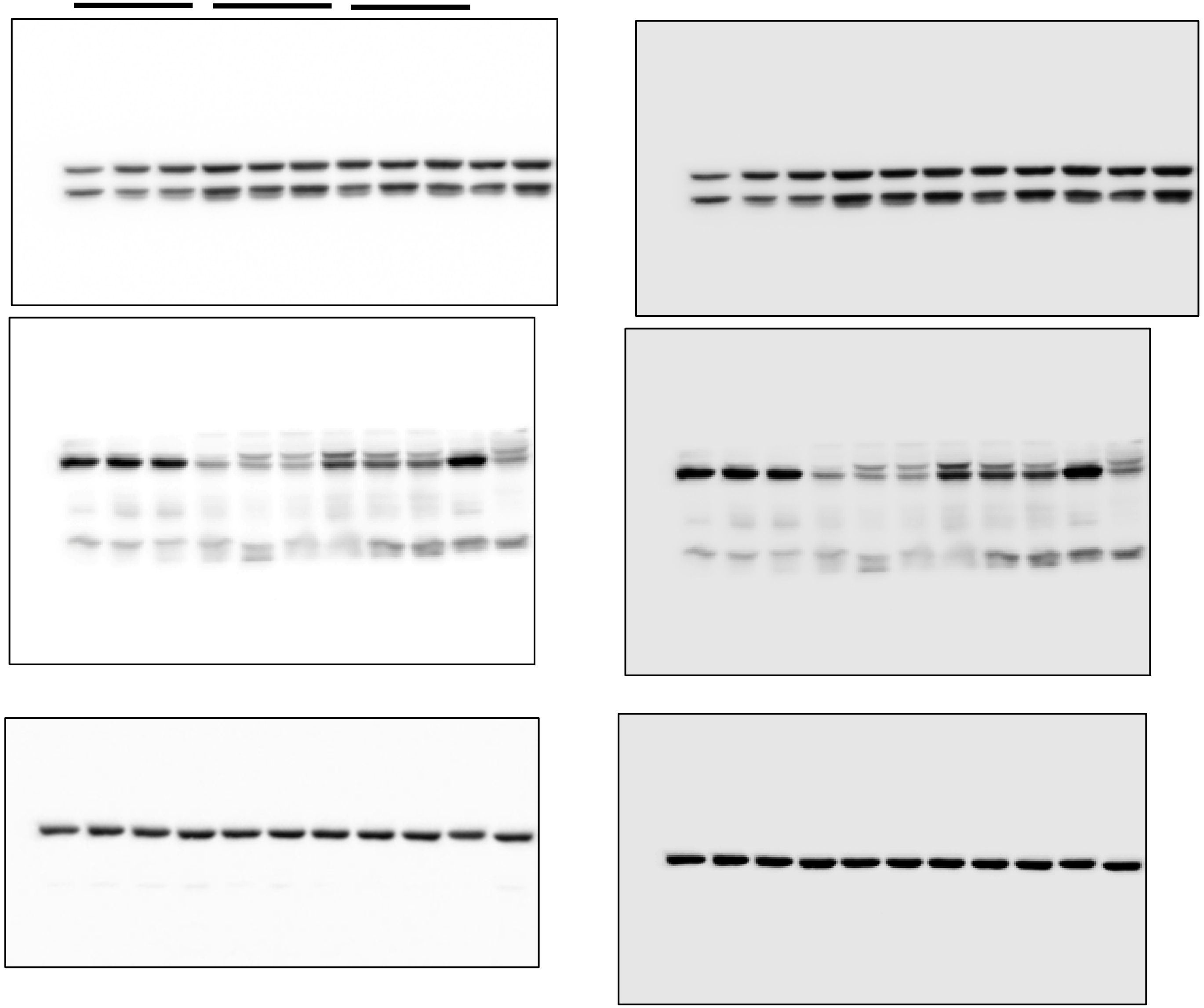

Supplement: Supplementary file 1 — Supplementary Information 1. [file 41598_2022_5140_MOESM1_ESM.pdf]
