# Supplementary material for: Pcyt2 deficiency causes age-dependant development of nonalcoholic steatohepatitis and insulin resistance that could be attenuated with phosphonoethylamine
Source: Sci Rep. 2022 Jan 20;12:1048. doi: 10.1038/s41598-022-05140-y (PMC8776951; doi:10.1038/s41598-022-05140-y)
Supplement: Supplementary file 2 — Supplementary Information 2. [file 41598_2022_5140_MOESM2_ESM.pdf]

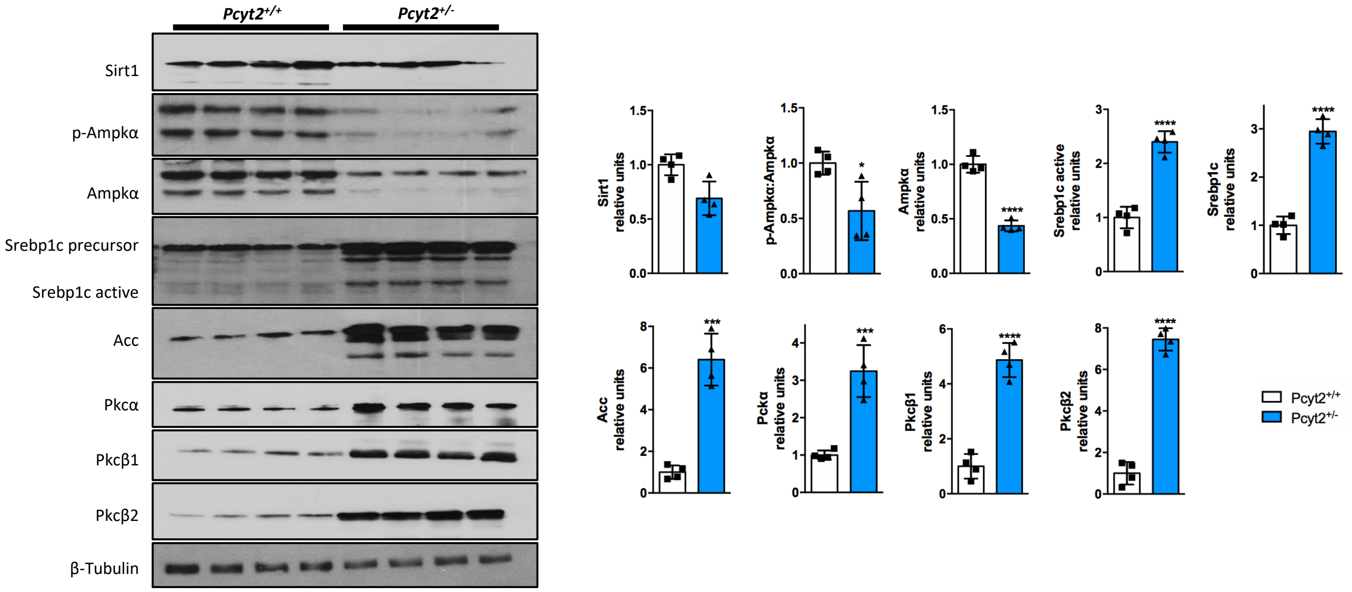

Supplementary Figure 1: **Mitochondria regulators are inhibited and lipid/lipogenic pathways are dramatically upregulated in fasted 6-mo *Pcyt2*$^{+/-}$.** Immunoblot analysis of lipid pathways in fasted *Pcyt2*$^{+/+}$ and *Pcyt2*$^{+/-}$ (n=4). Data are presented as mean ± SD. **p*<0.05, ***p*<0.01, ****p*<0.001, *****p*<0.0001.

Supplementary table 1. Primer Sequences and melting temperatures (Tm) for polymerase chain reaction (PCR) using mouse hepatic RNA.

Gene	Primer Forward 5'-3'	Primer Reverse 5'-3'	Tm (ºC)
TGH	GCTGGGTCCAGGACAACATT	GCGCAGGCAATGAACCATAA	59
LPL	GCTCGCACGAGCGCTCCATT	CCTCGGGCAGGGTGAAGGGAA	59
ATGL	CAACGCCACTCACATCTACGG	GGACACCTCAATAATGTTGGCAC	57
PPARα	GCTCACAGAATTTGCCAAGG	GTCATCCAGTTCTAAGGCATTG	57
PPARγ	CAGAAGTGCCTTGCTGTGGGG	CTTGGCTTTGGTCAGCGGG	57
CTL-1	GAACGCTCTGCGAGTGGCTGC	CGGCTTTAGCTCTCGGGCGT	49
PCYT1	ATGCACAGAGTTCAGCTAAAG	GGGCTTACTAAAGTCAACTTCAA	50
PSS1	CTGTTGTGCAATGGTGGTGG	GGCTGGCTTGGAACACAAAG	59.5
PSS2	GAGTGGCTGTCCCTGAAGAC	TCGTAGATCTCACGCATGGC	59
PSD	GTTTGCTGTCACGTGCCTGTG	CAGTGCAAGCCACATACGGG	59.5
PEMT	TGTTTGTGCTGTCCAGCTTC	TTCCAAAGATCCTTCATGGC	52
BHMT	TGGATTGGAACCCCGAGTTG	GGTGTGCATGTCCAAACCAC	59
TNFα	ACAGAAAGCATGATCCGCGA	GCTACAGGCTTGTCACTCGAA	56
TNFR	AAAGGGCACCTTTACGGCTT	GTGACATTTGCAAGCGGAGG	56
IL-10	TCTTGCACTACCAAAGCCACA	TTGGCAACCCAAGTTAACCCT	57
IL-6	GCCTTCTTGGGACTGATGCT	TGGAAATTGGGGTAGGAAGGAC	56
TGFβ1	AGGAGACGGAATACAGGGCT	CCACGTAGTAGACGATGGGC	57
TGFβ3	ATGACCCACGTCCCCTATCA	ACTCTGCCCGGAACAGATTG	57
IFNY	CGGCACAGTCATTGAAAGCC	TGCATCCTTTTTCGCCTTGC	57
GAPDH	ACCACAGTCCATGCCATCAC	TCCACCACCCTGTTGCTGTA	58

Abbreviations: ACC-acetyl-CoA carboxylase, Akt-protein kinase B, AMPK-AMP-activated protein kinase, ANGPTL4-angiopoietin-like 4, ANOVA-analysis of variance, ATGL-adipose triglyceride lipase, ATP-adenosine triphosphate, AUC-area under the curve, BCA-bicinchoninic acid, BMI-body mass index, BSA-bovine serum albumin, CD68- cluster of differentiation 68, CDP-cytidine diphosphate, CMP-cytidine monophosphate, COX-2-cyclooxygenase-2, CTL-1-choline transporter-like protein 1, CTP-cytidine triphosphate, DAG-diacylglycerol, EGR1-early growth response protein 1, EIF2α- Eukaryotic Initiation Factor 2 alpha, EK-ethanolamine kinase, EPT-CDP-ethanolamine:1,2-diacylglycerol ethanolamine-phosphotransferase, ERK-Extracellular signal-regulated kinase, ET/Pcyt2-CTP-phosphoethanolamine cytidylyltransferase, ETKO- CTP-phosphoethanolamine cytidylyltransferase knock out, Etn-ethanolamine, FA-fatty acid, FBS-fetal bovine serum, FOXO1- Forkhead Box O1, G6Pase-glucose 6-phosphate, G6PDH-glucose 6-phosphate dehydrogenase, GAPDH- glyceraldehyde 3-phosphate dehydrogenase, GK-glucokinase, GTT-glucose tolerance test, H&E-hematoxylin and eosin, HK-hexokinase, INFg-interferon gamma, IR-insulin receptor, IRS1-insulin receptor substrate 1, JNK- c-Jun N-terminal kinases, Keap1-kelch-like ECH-associated protein 1, L-Pk-liver pyruvate kinase, LSC- iquid scintillation counting, LXR-liver X receptor, MAM-ER mitochondria associated membranes, MAPK- Mitogen-activated protein kinase, mRNA-messenger ribonucleic acid, mTORC1/2-mammalian target of rapamycin complex 1/2, NAD-nicotinamide adenine dinucleotide, NADPH/ $NADP^+$-nicotinamide adenine dinucleotide phosphate, NAFLD-non-alcoholic fatty liver disease, NASH-nonalcoholic steatohepatitis, NF-kB-nuclear factor kappa-light-chain-enhancer of activated B cells, NIH- National Institutes of Health, Nrf2-nuclear factor erythroid 2-related factor 2, PAS-periodic acid-Schiff reagent, PC-phosphatidylcholine, PDK-phosphoinositide-dependent kinase-1, PE-phosphatidylethanolamine, PEMT-PE N-methyltransferase, PEPCK-phosphoenolpyruvate carboxykinase, PEtn-phosphoethanolamine, PGC1α- Peroxisome proliferator activated receptor gamma coactivator 1 alpha, Pi-inorganic phosphate, PI3K-phosphoinositide 3-kinase, PKA- protein kinase A, PKC-protein kinase C, PPAR-peroxisome proliferator-activated receptor, PS-phosphatidylserine, PSD-phosphatidylserine decarboxylase,

Supplementary Table 2 for Fig 2A. **Downregulated pathways in 2-mo *Pcyt2*$^{+/-}$ liver**

Figure 2A-a: MGI Mammalian Phenotype level 4, 2019

Term Overlap P-value Adjusted P-value Old P-value Old Adjusted P-value Odds Ratio Combined Score Genes

1. MP:0002628 hepatic steatosis 19/206 5.464855551992962E-5 0.06394902276836688 0 0 3.0408255347593585 29.84444911985981 ANK1;ELOVL5;NOS3;PEX11A;MAT1A;GNMT;HADHB;LGALS3;POR;PGRMC1;ACADL;ACX1;ACADM;ALDOB;APOB;PPARGC1A;PNPLA2;SIDT2;SLC25A13
2. MP:0005309 increased circulating ammonia level 5/14 5.9793382672619803E-5 0.06394902276836688 0 0 16.422018348623855 159.69781517242902 OAT;CPS1;ARG1;ASL;ASS1
3. MP:0011501 increased glomerular capsule space 5/20 3.929038056401208E-4 0.23151917642846842 0 0 9.850152905198776 77.2443646666549 INVS;GLIS3;SCTR;GNE;UOX
4. MP:0009355 increased liver triglyceride level 10/84 4.3294843651887503E-4 0.23151917642846842 0 0 4.011785282971724 31.070843428097586 GYS2;FADS2;LGALS3;PGRMC1;PANK1;PEX11A;LPIN2;SIDT2;SLC25A13;PNPLA2
5. MP:0009815 decreased prostaglandin level 4/13 6.584952774437638E-4 0.28170427969044215 0 0 13.117557251908398 96.09336361432368 FADS2;NOS3;LPAR3;FADS1
6. MP:0002118 abnormal lipid homeostasis13/141 7.970780246633689E-4 0.284158315792491 0 0 3.0206196787925697 21.550786252056398 ARSA;PCYT2;CYP4A14;ADIPOR2;CYP7A1;HADHB;ACADL;EHHADH;ANGPTL4;SLC37A4;PPARGC1A;MFSD2A;PNPLA2
7. MP:0005584 abnormal enzyme/coenzyme activity 14/171 0.001624151990121633 0.4962944438385961 0 0 2.6522095492025874 17.03453047198098 GKD;PANK1;GPT2;KMO;KLF15;ASS1;SLK;ACADL;CES1E;TMBIM1;ME1;ASL;LPIN2;PNPLA2
8. MP:0000598 abnormal liver morphology 17/233 0.001924728975720663 0.5146244098833123 0 0 2.344561266874351 14.660471529834393 INVS;ONECUT2;ARG1;EPO;UNC93B1;NOS3;ASPSCR1;MAT1A;CYP7A1;CRKL;GNMT; LGALS3;ACOX1;CTH;ALDOB;MFSD2A;SIDT2
9. MP:0005311 abnormal circulating amino acid level 6/43 0.0026998176910497155 0.6416566712394823 0 0 4.793841314515127 28.353514961655737 OAT;ARG1;CTH;ASL;KLF15;ASS1
10. MP:0000609 abnormal liver physiology 10/110 0.003436208878611819 0.7223244186275055 0 0 2.9647149460708784 16.819973708379674 AS3MT;EPO;PANK1;ELOVL5;ACOX1;NOS3;MAT1A;PPARGC1A;CYP7A1;RAD18

Figure 2b: GO Biological Process 2018

1. fatty acid metabolic process (GO:0006631) 15/106 2.017310155131874E-6 0.0029175606462129217 0 0 4.927138079311993 64.6132350140058 CPT1A;ABCD3;ACSM3;GPX4;ELOVL4;ACSL1;PLA2G1B;ADIPOR2;ACOX1;FASN;PCCB;ACOT1;ACADM;PPARGC1A;LPIN2
2. organonitrogen compound biosynthetic process (GO:1901566) 20/181 2.3576247646165025E-6 0.0029175606462129217 0 0 3.7286521058719466 48.31533669439376 OAT;GOT1;ELOVL4;ELOVL5;ARG1;GSTO1;GPT2;ASNS;FURIN;GPHN;ASS1;ALDH3A2;GLUD1;CPS1;VAPA;VAPB;ASL;CEPT1;LPIN2;MFSD2A

3. fatty acid oxidation (GO:0019395) 10/50 4.397605834839108E-6 0.003628024813742264 0 0 7.434899845916795 91.70540258718638 HADHB;ALDH3A2;ABCD3;ACADL;ACOX1;EHHADH;ACADM;ACAD11;ADIPOR2;PPARGC1A
4. fatty acid beta-oxidation (GO:0006635) 9/50 3.2408985307256655E-5 0.019556789706896935 0 0 6.51782363977486 67.37523081898888 HADHB;ABCD3;ACADL;ACOX1;EHHADH;ACADM;ACAD11;DECR2;CRAT
5. alpha-linolenic acid metabolic process (GO:0036109) 5/13 3.950866607453926E-5 0.019556789706896935 0 0 18.47572629969419 187.3252137256421 FADS2;ELOVL5;ACSL1;ACOX1;FADS1
6. regulation of primary metabolic process (GO:0080090) 15/139 5.5823126258719955E-5 0.023027039581721982 0 0 3.6096849328791825 35.35080804066797 CPT1A;PSMD12;ACSL1;PSMD13;PEX11A;CYP7A1;GLIPR1;PSMB4;ACOX1;CARM1;ME1;HMGCS2;ACADM;ANGPTL4;FADS1
7. regulation of lipid metabolic process (GO:0019216) 12/100 1.1019524030597333E-4 0.038961888536754856 0 0 4.057819305887312 36.97994960002394 CPT1A;GLIPR1;ACSL1;ACOX1;PEX11A;CARM1;ME1;HMGCS2;ACADM;ANGPTL4;CYP7A1;FADS1
8. fatty acid catabolic process (GO:0009062) 9/65 2.6656374259770733E-4 0.07207968712770826 0 0 4.7682692307692305 39.24236539435219 HADHB;ALDH3A2;ABCD3;ACADL;EHHADH;PCCB;ACADM;ACAD11;LPIN2
9. carnitine metabolic process (GO:0009437) 4/11 3.203541650120367E-4 0.07207968712770826 0 0 16.867175572519084 135.71470152266448 CPT1A;ACADL;ACADM;CRAT
10. urea cycle (GO:0000050) 4/11 3.203541650120367E-4 0.07207968712770826 0 0 16.867175572519084 135.71470152266448 CPS1;ARG1;ASL;ASS1
11. arginine metabolic process (GO:0006525) 4/11 3.203541650120367E-4 0.07207968712770826 0 0 16.867175572519084 135.71470152266448 CPS1;NOS3;ARG1;ASS1
12. phosphatidylethanolamine metabolic process (GO:0046337) 5/20 3.929038056401208E-4 0.07861361573734385 0 0 9.850152905198776 77.2443646666549 PCYT2;ETNK2;HRASLS5;CEPT1;LPIN2
13. long-chain fatty acid metabolic process (GO:0001676) 8/55 4.129200018527152E-4 0.07861361573734385 0 0 5.044677582769553 39.30942261235577 FADS2;CPT1A;ACADL;ACSL1;ELOVL5;ACOX1;ACOT1;FADS1
14. mitochondrial transport (GO:0006839) 13/135 5.277643310456708E-4 0.09330119423843108 0 0 3.170164441963153 23.924789492261766 PNPT1;BAD;TIMM9;BNIP3;AIP;ATP5C1;ATP5G3;ATP5L;PINK1;GRPEL1;SLC25A20;SLC25A22;SLC25A13

ELOVL5;ACOX1;ACOT1

Supplementary Table 3 for Figure 2B. **Upregulated pathways in 2-mo *Pcyt2*$^{+/-}$ liver**

Elsevier Pathway Collection

	Term	Overlap	P-value	Adjusted P-value	Old P-value	Old Adjusted P-value	Odds Ratio	Combined Score	Genes
1.	**AngiopoietinR -> STAT Signaling**	3/8	6.695238747960458E-4	0.30440483811407787	0	0	24.976119402985073	182.54905138092414	**ANGPT4;ANGPT2;LCK**
2.	**AngiopoietinR -> FOXO Signaling**	3/9	9.86725569251468E-4	0.30440483811407787	0	0	20.812366737739872	144.04485859125822	**ANGPT4;ANGPT2;FOXO1**
3.	GUCY2C in Intestinal Ion and Fluid Homeostasis	3/12	0.002451382720992579	0.504167712950807	0	0	13.872778962331202	83.39070376195338	SLC9A3;GUCA2B;GUCA2A
4.	Genes with Mutations Associated with Urolithiasis	2/6	0.007828126089712499	0.8517865161452929	0	0	20.770212765957446	100.73619909675153	CASR;SLC34A1
5.	**IGF1R -> CEBPA/FOXO1A Signaling**	2/9	0.017926636569026717	0.8517865161452929	0	0	11.866869300911855	47.72223035652416	**IGF2;FOXO1**
6.	Dioxin Role in Endometriosis	2/11	0.0265489417774117	0.8517865161452929	0	0	9.228841607565013	33.489301013599345	**ARNT;**CYP1B1
7.	Glioblastoma, Proneural Subtype	2/11	0.0265489417774117	0.8517865161452929	0	0	9.228841607565013	33.489301013599345	SOX2;TCF4
8.	Hematopoietic Cell Lineage: B-cell (mouse)	4/49	0.028152708219656288	0.8517865161452929	0	0	3.700474833808167	13.211108582205329	ANGPT2;EBF1;ID3;DNTT
**9.**	**Endocannabinoids Role in Sleep Regulation**	2/12	0.031368464329064434	0.8517865161452929	0	0	8.305531914893617	28.753354580170335	**NAPEPLD;DAGLB**
10.	Dioxin Induced Chloracne (Hypothesis)	2/12	0.031368464329064434	0.8517865161452929	0	0	8.305531914893617	28.753354580170335	EPGN;ARNT

BioPlanet 2019

	Term	Overlap	P-value	Adjusted P-value	Old P-value	Old Adjusted P-value	Odds Ratio	Combined Score	Genes
1.	Proepithelin conversion to epithelin **and wound repair contro**l	2/8	0.014162203067316765	0.9300147537850102	0	0	13.845390070921987	58.94229858120675	CELA3B;CELA1
2.	**Binding of RNA by insulin-like growth factor 2 mRNA binding proteins** (IGF2BPs/IMPs/VICKZs)	2/8	0.014162203067316765	0.9300147537850102	0	0	13.845390070921987	58.94229858120675	IGF2;ACTB
3.	**Activation of pro-caspase 8**	2/9	0.017926636569026717	0.9300147537850102	0	0	11.866869300911855	47.72223035652416	**CASP8;TNFSF10**
4.	**Transport of inorganic cations/anions and amino acids/oligopeptides**	6/95	0.02500765895155889	0.9300147537850102	0	0	2.812219703910884	10.37307807197569	**SLC9A3;SLC6A19;SLC7A8;SLC34A1;SLC7A11;SLC12A6**
5.	**Glutathione biosynthesis and recycling**	2/11	0.0265489417774117	0.9300147537850102	0	0	9.228841607565013	33.489301013599345	GCLC;CNDP2
6.	**G-protein activation**	3/28	0.02763591893096248	0.9300147537850102	0	0	4.990106609808103	17.90769090377881	**GNAZ;GNB4;GNG12**
7.	**Homologous recombination**	3/29	0.030298923376566384	0.9300147537850102	0	0	4.797933409873709	16.776660748174137	POLD3;TOP3B;XRCC3

8. **Myometrial relaxation and contraction pathways** 8/155 0.03098226588517045
0.9300147537850102 0 0 2.2731644381890685 7.897746830699842
GUCY1A3;RGS2;PKIA;GNB4;GUCA2B;GNG12;ACTB;GUCA2A
9. **SODD/TNFR1 signaling pathway** 2/12 0.031368464329064434 0.9300147537850102 0
0 8.305531914893617 28.753354580170335 CASP8;MADD
10. **Amino acid transport across the plasma membrane** 3/31 0.0360143368120434
0.9300147537850102 0 0 4.454766981419433 14.806924553684793
SLC6A19;SLC7A8;SLC7A11

Reactome 2016

1. **TRAIL signaling** Homo sapiens R-HSA-75158 2/7 0.010789024695074069
0.9999952869426106 0 0 16.61531914893617 75.25453371818327
CASP8;TNFSF10
2. **Insulin-like Growth Factor-2 mRNA Binding Proteins** (IGF2BPs/IMPs/VICKZs) bind RNA Homo sapiens R-HSA-428359 2/8 0.014162203067316765 0.9999952869426106 0 0
13.845390070921987 58.94229858120675 IGF2;ACTB
3. **Thromboxane signaling** through TP receptor Homo sapiens R-HSA-428930 3/23
0.016287439904619384 0.9999952869426106 0 0 6.239232409381663
25.689172355795996 **TBXA2R;GNB4;GNG12**
4. **CASP8 activity i**s inhibited Homo sapiens R-HSA-5218900 2/11 0.0265489417774117
0.9999952869426106 0 0 9.228841607565013 33.489301013599345
CASP8;TNFSF10
5. **Regulation by c-FLIP** Homo sapiens R-HSA-3371378 2/11 0.0265489417774117
0.9999952869426106 0 0 9.228841607565013 33.489301013599345
CASP8;TNFSF10
6. **Dimerization of procaspase-8** Homo sapiens R-HSA-69416 2/11 0.0265489417774117
0.9999952869426106 0 0 9.228841607565013 33.489301013599345
CASP8;TNFSF10
7. **G-protein activation** Homo sapiens R-HSA-202040 3/28 0.02763591893096248
0.9999952869426106 0 0 4.990106609808103 17.90769090377881
GNAZ;GNB4;GNG12
8. **Transport of inorganic cations/anions and amino acids/oligopeptide**s Homo sapiens R-HSA-425393
6/99 0.029844089887825203 0.9999952869426106 0 0 2.69071023120587
9.449151304645508 **SLC9A3;SLC6A19;SLC7A8;SLC34A1;SLC7A11;SLC12A6**
9. **mRNA 3'-end processing** Homo sapiens R-HSA-72187 4/51 0.03201949066091937
0.9999952869426106 0 0 3.5426441171122023 12.191692586029836
FYTTD1;FIP1L1;CPSF3;SRSF11
10. Post-Elongation **Processing of Intron-Containing pre-mRNA** Homo sapiens R-HSA-112296 4/51
0.03201949066091937 0.9999952869426106 0 0 3.5426441171122023
12.191692586029836 FYTTD1;FIP1L1;CPSF3;SRSF11,CASP8;TNFSF10

GO Biological Processes 2018

1. negative regulation of blood vessel endothelial cell migration (GO:0043537) 5/31
7.333995806897917E-4 0.9359433242991512 0 0 8.030802174271125
57.96488353811351 **ANGPT4;ANGPT2;TBXA2R;MMRN2;MEOX2**
2. negative regulation of cell migration involved in sprouting angiogenesis (GO:0090051) 3/14
0.003916065051671796 0.9359433242991512 0 0 11.34929249854623
62.905359703868335 TBXA2R;MMRN2; MEOX2

3. membrane lipid catabolic process (GO:0046466) 2/8 0.014162203067316765 0.9359433242991512 0 0 13.845390070921987 58.94229858120675 PPT1;CYP1B1
4. **negative regulation of protein polymerization** (GO:0032272) 3/22 0.014410698981294951 0.9359433242991512 0 0 6.567949725058916 27.846690544326467 DYRK1A;HIP1R;PFN2
5. **negative regulation of sprouting angiogenesis** (GO:1903671) 3/22 0.014410698981294951 0.9359433242991512 0 0 6.567949725058916 27.846690544326467 TBXA2R;MMRN2;MEOX2
6. regulation of **epidermal growth factor**-activated receptor activity (GO:0007176) 3/24 0.01829466228131285 0.9359433242991512 0 0 5.9418215047212914 23.77409499159111 EPGN;NCK2;EREG
7. RNA catabolic process (GO:0006401) 4/43 0.01829529208018193 0.9359433242991512 0 0 4.271093578785886 17.089121702331116 RNASEH2B;DIS3L;RNASE6;HSPA1A
8. positive regulation of nuclear division (GO:0051785) 3/25 0.020432949995376072 0.9359433242991512 0 0 5.671447955030045 22.065372194915977 EPGN;IGF2;EREG
9. positive regulation of epidermal growth factor-activated receptor activity (GO:0045741) 2/10 0.022062059633939514 0.9359433242991512 0 0 10.382978723404255 39.59960002889824 EPGN;EREG
10. negative regulation of cardiac muscle hypertrophy (GO:0010614) 2/11 0.0265489417774117 0.9359433242991512 0 0 9.228841607565013 33.489301013599345 **RGS2;FOXO1**

GO Human phenotype

1. Narrow forehead (HP:0000341) 5/65 0.018581530379705388 0.811679484707992 0 0 3.4739471805852964 13.845719353687516 CSPP1;NPHP1;DYRK1A;TCF4;SLC12A6
2. **Hypoalphalipoproteinemia** (HP:0003233) 2/10 0.022062059633939514 0.811679484707992 0 0 10.382978723404255 39.59960002889824 **ALMS1;PPP1R3A**
3. Asthma (HP:0002099) 4/51 0.03201949066091937 0.811679484707992 0 0 3.5426441171122023 12.191692586029836 CASP8;CDSN;ALMS1;LIFR
4. **Rhabdomyosarcoma** (HP:0002859) 2/13 0.03650249782103007 0.811679484707992 0 0 7.550096711798839 24.99364828626234 **PMS2;FOXO1**
5. **Insulin-resistant diabetes mellitus** (HP:0000831) 2/13 0.03650249782103007 0.811679484707992 0 0 7.550096711798839 24.99364828626234 **ALMS1;PPP1R3A**
6. Polydipsia (HP:0001959) 2/14 0.04193358005675239 0.811679484707992 0 0 6.920567375886525 21.94974443882456 CASR;NPHP1
7. Abnormal drinking behavior (HP:0030082) 2/14 0.04193358005675239 0.811679484707992 0 0 6.920567375886525 21.94974443882456 CASR;NPHP1
8. **Complement deficiency** (HP:0004431) 2/15 0.04764489483849146 0.811679484707992 0 0 6.387888707037643 19.444604146539334 CFH;C8A
9. **Maternal diabetes** (HP:0009800) 2/15 0.04764489483849146 0.811679484707992 0 0 6.387888707037643 19.444604146539334 **SOX2;PPP1R3A**
10. **Hyperuricemia** (HP:0002149) 2/16 0.05362025129539057 0.811679484707992 0 0 5.931306990881459 17.353986796469474 **ALMS1;PPP1R3A**

Supplementary Table 4 for Fig. 3A Downregulated pathways in 6-mo *Pcyt2*$^{+/-}$ liver

Fig. 3A-a KEGG pathway Mouse

	Term	Overlap	P-value	Adjusted P-value	Old P-value	Old Adjusted P-value	Odds Ratio	Combined Score	Genes
1.	Insulin signaling pathway	17/139	3.3542736653252626E-27	5.36683786452042E-25	0	0	125.65685543964233	7659.986902198042	INS1;PRKCI;MAP2K1;PKLR;BRAF;PIK3R2;SORBS1;CBL;GCK;PTPRF;HK2;ACACA;PIK3CA;SOS1;RAF1;FBP1;PCK2
2.	Chronic myeloid leukemia	8/76	1.6651543929129943E-12	1.3321235143303954E-10	0	0	75.49525616698293	2047.5146387729324	MAP2K1;PIK3CA;BRAF;PIK3R2;SOS1;RAF1;CBL;BCL2L1
3.	FoxO signaling pathway	9/132	3.2306236031537076E-12	1.523046855804862E-10	0	0	48.385365853658534	1280.1967477703968	INS1;MAP2K1;PIK3CA;BRAF;PIK3R2;SOS1;RAF1;IGF1R;PCK2
4.	ErbB signaling pathway	8/84	3.8076171395121555E-12	1.523046855804862E-10	0	0	67.52122241086587	1775.4042067411829	MAP2K1;PIK3CA;BRAF;PIK3R2;SOS1;RAF1;CBL;NCK1
5.	Prostate cancer	8/97	1.2397993514637874E-11	3.4869852094777943E-10	0	0	57.62087712939471	1447.0611181639738	INS1;MAP2K1;PIK3CA;BRAF;PIK3R2;SOS1;RAF1;IGF1R
6.	mTOR signaling pathway	9/154	1.3076194535541728E-11	3.4869852094777943E-10	0	0	40.99862068965517	1027.4347719136917	INS1;MAP2K1;PIK3CA;GRB10;BRAF;PIK3R2;SOS1;RAF1;IGF1R
7.	Prolactin signaling pathway	7/72	8.15064064653917E-11	1.86300357635181E-9	0	0	66.95769230769231	1555.4499238974392	INS1;MAP2K1;PIK3CA;PIK3R2;SOS1;RAF1;GCK
8.	AMPK signaling pathway	8/126	1.0351024557091974E-10	1.9441581916696194E-9	0	0	43.396391470749045	997.7416474812381	INS1;PIK3CA;PPARG;PIK3R2;FBP1;ACACA;IGF1R;PCK2
9.	Glioma	7/75	1.0935889828141608E-10	1.9441581916696194E-9	0	0	63.994025735294116	1467.7916758179063	MAP2K1;PIK3CA;BRAF;PIK3R2;SOS1;RAF1;IGF1R
10.	Proteoglycans in cancer	9/203	1.5584661687681132E-10	2.493545870028981E-9	0	0	30.567525773195875	690.2804159933993	MAP2K1;PIK3CA;FRS2;BRAF;PIK3R2;SOS1;RAF1;CBL;IGF1R

Fig. 3A-b GO Biological process

	Term	Overlap	P-value	Adjusted P-value	Old P-value	Old Adjusted P-value	Odds Ratio	Combined Score	Genes
1.	cellular response to insulin stimulus (GO:0032869)	9/110	6.091506918849882E-13	3.9533879903335734E-10	0	0	58.99009900990099	1659.1974499768214	PRKCI;PKLR;PPARG;PIK3R2;SORBS1;SOS1;GCK;IGF1R;PCK2

2. response to insulin (GO:0032868) 7/71 7.368534951486453E-11 2.3910895917573538E-8 0 0 68.00732421875 1586.693647227742 PRKCI;PKLR;PPARG;PIK3R2;SORBS1;GCK;PCK2
3. cellular response to peptide hormone stimulus (GO:0071375) 7/110 1.6693108435580462E-9 3.6112757915639067E-7 0 0 42.17415048543689 852.375638700225 PRKCI;PKLR;PPARG;PIK3R2;SORBS1;GCK;PCK2
4. cellular glucose homeostasis (GO:0001678) 5/33 4.9264155952384554E-9 7.993109303274394E-7 0 0 104.69012605042016 2002.581216558548 PIK3R2;HK2;GCK;IGF1R;PCK2
5. transmembrane receptor protein tyrosine kinase signaling pathway (GO:0007169) 9/396 5.352640065119736E-8 6.947726804525419E-6 0 0 15.173643410852714 254.0536899204188 PIK3CA;FRS2;PIK3R2;SORBS1;SOS1;CBL;FRS3;IGF1R;NCK1
6. insulin receptor signaling pathway (GO:0008286) 5/67 1.9104003837882453E-7 2.066416415130952E-5 0 0 47.19876660341556 730.2018668253547 PIK3CA;PIK3R2;SORBS1;SOS1;IGF1R
7. pyruvate metabolic process (GO:0006090) 4/46 1.8986985018884242E-6 1.760364753893696E-4 0 0 54.201360544217685 714.067255553722 PKLR;GCK;HK2;PCK2
8. glycolytic process (GO:0006096) 3/23 1.1816441035959648E-5 5.842794088959005E-4 0 0 83.0875 942.7123277486376 PKLR;HK2;GCK
9. glucose homeostasis (GO:0042593) 4/74 1.287349249979027E-5 5.842794088959005E-4 0 0 32.475102040816324 365.6806971932668 PPARG;PIK3R2;GCK;HK2
10. axon guidance (GO:0007411) 5/158 1.3383789991443453E-5 5.842794088959005E-4 0 0 19.038831218762013 213.64360263511566 DOK2;PIK3CA;GRB10;FRS2;SOS1

Supplementary Table 5 for Fig. 3B **Upregulated pathways in 6-mo *Pcyt2*$^{+/-}$ liver**

Wiki Pathway 2019 Mouse

	Term	Overlap	P-value	Adjusted P-value	Old P-value	Old Adjusted P-value	Odds Ratio	Combined Score	Genes
1.	Insulin Signaling WP65	16/159	7.480853932910914E-25	5.685448989012295E-23	0	0	110.88671328671329	6160.009786612667	PTPN1;PRKCI;JUN;SHC1;PDPK1;SLC2A1;PIK3R2;PIK3R1;FOS;AKT2;RPS6KA1;EIF4EBP1;AKT1;MAPK1;GRB2;RAF1
2.	EGFR1 Signaling Pathway WP572	14/178	3.724366304165368E-20	1.4152591955828397E-18	0	0	76.82926829268293	3437.096004828217	CEBPB;PRKCI;JUN;SHC1;ARAF;PIK3R2;PIK3R1;FOS;RPS6KA1;AKT1;MAPK1;GRB2;KRAS;RAF1
3.	Focal Adhesion-PI3K-Akt-mTOR-signaling pathway WP2841	15/324	4.11453478524301E-18	8.633713839088465E-17	0	0	45.43458159963014	1818.8374386403746	SREBF1;PDPK1;SLC2A1;IRS2;PIK3R2;PIK3R1;VEGFA;AKT2;AKT3;EIF4EBP1;AKT1;MAPK1;GRB2;KRAS;RAF1
4.	Focal Adhesion WP85	13/185	4.544059915309719E-18	8.633713839088465E-17	0	0	65.03943377148634	2597.200899846062	JUN;SHC1;PDPK1;ARAF;PIK3R2;PIK3R1;VEGFA;AKT2;AKT3;AKT1;MAPK1;GRB2;RAF1
5.	IL-6 signaling Pathway WP387	11/99	1.3368163335801963E-17	2.031960827041898E-16	0	0	99.38	3861.2762999138718	CEBPB;JUN;SHC1;EIF4EBP1;AKT1;MAPK1;PIK3R2;GRB2;PIK3R1;FOS;RAF1
6.	IL-5 Signaling Pathway WP151	10/69	2.860656750433226E-17	3.623498550548752E-16	0	0	129.7588005215124	4942.888408930943	JUN;SHC1;RPS6KA1;AKT1;MAPK1;PIK3R2;GRB2;KRAS;PIK3R1;RAF1
7.	Integrin-mediated Cell Adhesion WP6	10/100	1.4039043292691922E-15	1.5242389860636944E-14	0	0	84.93162393162393	2904.620706154356	SHC1;PDPK1;AKT2;AKT3;ARAF;AKT1;MAPK1;PIK3R2;GRB2;RAF1
8.	ESC Pluripotency Pathways WP339	10/118	7.737110119024626E-15	7.350254613073394E-14	0	0	70.71225071225071	2297.6353532769176	JUN;AKT2;AKT3;ARAF;AKT1;MAPK1;PIK3R2;GRB2;FOS;RAF1
9.	IL-2 Signaling Pathway WP450	9/76	8.772632206983754E-15	7.408000530341836E-14	0	0	98.99004975124379	3204.024749007802	SHC1;AKT1;MAPK1;IRS2;PIK3R2;GRB2;KRAS;PIK3R1;RAF1
10.	Novel Jun-Dmp1 Pathway WP3654	7/26	2.1117806814715364E-14	1.6049533179183675E-13	0	0	253.3847549909256	7978.746344732662	JUN;RRAS;ARAF;MAPK1;KRAS;FOS;RAF1

Jensen Diseases

	Term	Overlap	P-value	Adjusted P-value	Old P-value	Old Adjusted P-value	Odds Ratio	Combined Score	Genes
1.	Hyperglycemia	11/108	3.621127000780859E-17	5.540324311194714E-15	0	0	90.11835051546392	3411.6249295109155	PTPN1;SREBF1;JUN;G6PC;LEP;SLC2A1;AKT1;IRS2;PPARG;RETN;VEGFA
2.	Hyperinsulinism	7/54	5.48763317803716E-12	4.1980393811984273E-10	0	0	102.28833455612619	2652.1855444627995	SREBF1;G6PC;SLC2A1;AKT1;IRS2;PPARG;RETN
3.	Fatty liver disease	7/85	1.4704016405222077E-10	7.49904836666326E-9	0	0	61.5393457117595	1393.2701928314445	SREBF1;ACOX1;LEP;AKT1;IRS2;PPARG;RETN

4. Arthritis 8/186 1.1652877018418393E-9 4.4572254595450357E-8 0 0 31.759229534510432 653.2968102506449 JUN;LEP;AKT1;MAPK1;PPARG;RETN;FOS;VEGFA
5. Cancer 9/300 2.2503138803435725E-9 6.885960473851332E-8 0 0 22.534936998854526 448.72008525522403 PRKCI;JUN;AKT2;ARAF;AKT1;RRAS2;KRAS;FOS;RAF1
6. Lipodystrophy 4/28 1.7546733069703368E-7 4.474416932774359E-6 0 0 103.85416666666667 1615.5359919124223 SREBF1;LEP;PPARG;RETN
7. Neutropenia 4/72 8.334015310359583E-6 1.778082598445152E-4 0 0 36.5735294117647 427.7334679739925 G6PC;ERCC1;KRAS;VEGFA
8. Diabetes mellitus 4/74 9.297163913438704E-6 1.778082598445152E-4 0 0 35.525 411.5855862039443 AKT2;LEP;PPARG;RETN
9. Noonan syndrome 2/5 3.1392977135267624E-5 5.336806112995496E-4 0 0 391.3921568627451 4058.3164477642435 KRAS;RAF1
10. Lung disease 4/119 6.057300576276312E-5 9.148899989723336E-4 0 0 21.575 209.52909068321378 JUN;AKT1;PPARG;VEGFA
